# Providing Measurement, Evaluation, Accountability, and Leadership Support (MEALS) for Non-communicable Diseases Prevention in Ghana: Project Implementation Protocol

**DOI:** 10.3389/fnut.2021.644320

**Published:** 2021-08-18

**Authors:** Amos Laar, Bridget Kelly, Michelle Holdsworth, Wilhemina Quarpong, Richmond Aryeetey, Gideon Senyo Amevinya, Akua Tandoh, Charles Agyemang, Francis Zotor, Matilda E. Laar, Kobby Mensah, Dennis Laryea, Gershim Asiki, Rebecca Pradeilles, Daniel Sellen, Mary R. L'Abbe, Stefanie Vandevijvere

**Affiliations:** ^1^Department of Population, Family and Reproductive Health, School of Public Health, University of Ghana, Accra, Ghana; ^2^Early Start, School of Health and Society, University of Wollongong, Wollongong, NSW, Australia; ^3^UMR MoISA (Montpellier Interdisciplinary Centre on Sustainable Agri-Food Systems), (Univ Montpellier, CIRAD, CIHEAM-IAMM, INRAE, Institut Agro, IRD), Montpellier, France; ^4^Department of Public and Occupational Health, Amsterdam University Medical Centre, University of Amsterdam, Amsterdam, Netherlands; ^5^Department of Family and Community Health, University of Health and Allied Sciences, Ho, Ghana; ^6^Department Family and Consumer Sciences, School of Agriculture, University of Ghana, Accra, Ghana; ^7^Department of Marketing and Entrepreneurship, University of Ghana Business School, University of Ghana, Accra, Ghana; ^8^Non-communicable Disease Programme, Ghana Health Service, Accra, Ghana; ^9^African Population and Health Research Center, Nairobi, Kenya; ^10^School of Sport, Exercise and Health Sciences, Loughborough University, Loughborough, United Kingdom; ^11^Department of Nutritional Sciences, University of Toronto, Toronto, ON, Canada; ^12^Sciensano, Service of Lifestyle and Chronic Diseases, Brussels, Belgium

**Keywords:** food policy, non-communicable diseases, food marketing, food provision, food environments, community readiness, Ghana, protocol

## Abstract

**Background:** This study describes the rationale, adaptation, and final protocol of a project developed to address the increase in obesity and nutrition-related non-communicable diseases (NR-NCDs) in Ghana. Code-named the Measurement, Evaluation, Accountability, and Leadership Support for NCDs (MEALS4NCDs) project, it aims to measure and support public sector actions that create healthy food marketing, retail, and provisioning environments for Ghanaian children using adapted methods from the International Network for Food and Obesity/NCDs Research Monitoring and Action Support (INFORMAS).

**Methods:** The protocol for this observational study draws substantially from the INFORMAS' Food Promotion and Food Provision Modules. However, to appraise the readiness of local communities to implement interventions with strong potential to improve food environments of Ghanaian children, the MEALS4NCDs protocol has innovatively integrated a local community participatory approach based on the community readiness model (CRM) into the INFORMAS approaches. The setting is Ghana, and the participants include health and nutrition policy-makers, nutrition and food service providers, consumers, school authorities, and pupils of Ghanaian basic schools.

**Results:** The study establishes a standardized approach to providing implementation science evidence for the prevention of non-communicable diseases (NCDs) in Ghana. It demonstrates feasibility and the innovative application of the INFORMAS expanded food promotion and food provision modules, together with the integration of the CRM in a lower-middle income setting.

**Conclusion:** The research will facilitate the understanding of the processes through which the INFORMAS approach is contextualized to a lower-middle income African context. The protocol could be adapted for similar country settings to monitor relevant aspects of food environments of children.

## Introduction

Non-communicable diseases represent the leading cause of death globally; they were responsible for 70% of the 56 million deaths in the world in 2015 (1). In some African countries, non-communicable diseases (NCDs) are linked with over 50% of all reported adult deaths (1); the estimate is over 40% in Ghana (2). Although relatively low when one-time morbidity and mortality rates are compared, Africa is experiencing an alarming surge in obesity (3–7) and other nutrition-related non-communicable diseases (NR-NCDs) (8–11). This rise has been partly attributed to modifiable environmental factors such as diet and nutrition-related exposures. For instance, there is a rapid change toward increased consumption of obesogenic, energy-dense nutrient-poor foods, (6, 12) – consistent with the so-called “nutrition transition” (dietary shifts from traditional minimally processed diets toward “modern” diets, which are usually high in fats, salt, and sugars, and low in important nutrients). Abrahams et al. (13) have reported that several African countries are at different stages of nutrition transition. Ghana is considered to be at an advanced stage given that it is experiencing rapid urbanization, and increasing overweight/obesity and diet-related NCDs (14, 15). Overall, in Ghana, the prevalence of overweight/obesity among women of fertility age has increased from 10 in 1993 to 40% in 2014 (14, 16). Recently, Aryeetey et al. (17) estimated the prevalence of childhood overweight and obesity in Ghana to be 17%, from a sample of 3,089 children aged 9–15 years old. The studies of Agyemang et al. (18) and Ofori-Asenso et al. (7) show that rates are higher in urban than rural areas in Ghana. Regarding dietary intakes, household data from the 2014 nationally representative Demographic and Health Survey (DHS) (14) indicate that Ghanaian households frequently consume foods with high content of added sugar (e.g., sugar-sweetened non-alcoholic beverages – SSBs), and sodium [e.g., bouillon cubes (70% sodium), salted dried fish (36%), foods processed with salt (84%)], and foods high in calories, but infrequently consume fruits or vegetables (14, 19).

The recent outdoor food advertising assessment we conducted in urban Ghana identified SSBs as the most widespread food/beverage sold or advertised (20–22). However, findings from the Healthy Food Environment Policy Index (Food-EPI) exercise of Ghana that assessed the extent of implementation of food environment policies showed that there is limited government action to regulate the marketing and sale of SSBs and other unhealthy foods (23). Similar revelations have been made in Kenya (20, 24). These studies amplify existing poverty data on food environment research and the need for action in the African region. It has long been recognized that food environments of people are a critical determinant of what they eat. Food environments are conceptualized as the collective physical, economic, policy, and socio-cultural surroundings, opportunities, and conditions that influence food consumption patterns of people (25–27). Unhealthy food environments are not only linked to suboptimal dietary behavior, but also with adverse health outcomes, such as obesity and NR-NCDs.

In response, global and regional efforts, such as the development of the WHO Afro Nutrient Profiling System (28), and local efforts are beginning to take hold. Globally, the International Network for Food and Obesity/Non-communicable Diseases Research, Monitoring and Action Support (INFORMAS) was set up to monitor and benchmark food environments and policies internationally. Among others, INFORMAS seeks to increase the accountability of governments and the food industry for action to reduce diet-related NCDs and their associated sequelae (27). Locally, the government of Ghana published national NCDs prevention policies in 2012 and 2020, which recognizes the need for interventions (such as regulating the advertising of unhealthy foods and non-alcoholic beverages particularly to children) to promote healthy diets as crucial (2, 29). However, given its traditional focus on addressing communicable diseases, and maternal and child health and undernutrition, existing initiatives by the health system to address NR-NCDs are nascent, limited in scope, scale, and success (23, 30). Existing policies have mainly been informed by evidence from high-income countries (HICs) and have paid little attention to local evidence on the food environment. Thus, there is an unmet need for context-appropriate evidence, which both local and regional policy-makers can use to create healthy food environments and promote nutrient-rich, healthy diets that have the potential to prevent NR-NCDs, as well as other forms of malnutrition – particularly among children.

The primary target population of the project, children aged 17 years or younger, was informed by three considerations. First, the global, regional, and local rise in overweight and obesity among children and adolescents; second, the project team response to calls articulated in global public health resolutions, guidelines, and frameworks to tackle food marketing that targets children (see World Health Assembly Resolution 63.14 and subsequent mandating framework for implementation); the third consideration is a request from Ghanaian stakeholders such as policy-makers to address the gaps identified by the previous study. The proposed study is relevant to the local context, and there is evidence of policy-maker interest. The recent efforts at benchmarking food environment policies in Ghana (31) identified gaps, as well as interventions to improve the Ghanaian food environments. The top four recommendations were “Food promotion and Food provision”-related. The recommendations included requests for “*the Government to pass a legislation to regulate the marketing (promotion, sponsorship, advertisement) and sale of food and drink with added sugars, and other nutrients of concern (saturated fatty acids/trans fats, salt) in all media, in the school environment, and other child-laden settings. The Government should institute measures to remedy the deficiencies associated with the School Feeding Programme”* (31).

In addition, these actions are aligned with the World Health Assembly (WHA) Resolution WHA63.14 (32). “In May 2010, the World Health Assembly (WHA), through resolution WHA63.14, endorsed a set of recommendations on the marketing of foods and non-alcoholic beverages to children. The main purpose of the recommendations was to guide efforts by Member States in designing new policies, or strengthening existing policies, on food marketing communications to children in order to reduce the impact of marketing foods high in saturated fats, trans-fatty acids, free sugars, or salt. Following this, a framework has been developed in response to the mandate of resolution WHA63.14 and is aimed at policy-makers wanting to apply the recommendations in their individual territories” (33). The project we describe pays heed.

Regarding school feeding, the Ghana government has, since 2005, been implementing the school feeding programme, aimed at providing a warm and nutritionally adequate locally produced meal every school day to school children in public schools in selected disadvantaged districts across the country (34). In 2019, the programme reached over 54.6% of school-enrolled pupils across Ghana – providing one hot meal a day to over 2.6 million students in 8,683 schools. However, we found evidence of challenges in various aspects of the programme. Fernandes et al. (35) had previously reported that the nutritional quality of the foods provided through the school feeding programme is, however, questionable due to low meal subsidy. Another study has argued that, aside from low meal subsidy, the capacity of some of the caterers recruited may affect the nutritional quality of the meals (15).

It is worth noting that, although not the primary target population of the project, the proposed interventions have positive externalities for adults – given that NCDs are an important public health challenge for both children and adults and the explicit links between child and adult disease risk, public health interventions such as those proposed in this study, ought to adopt a whole-of-life approach. Addressing the problem of obesity in children indirectly addresses the same in adulthood. Childhood overweight and obesity have been linked to severe obesity in adulthood, type 2 diabetes, hypertension, and coronary heart disease (28, 36, 37).

## Aims

This study describes the protocol of a research – the Measurement, Evaluation, Accountability, and Leadership Support for NCDs (MEALS4NCDs) prevention project that aims to measure and support public sector actions that create healthy food marketing, retail, and provision environments for children in Ghana. In its entirety, the project will describe the nature and extent of unhealthy foods and non-alcoholic beverage promotion on television, in stores, and in and around schools; and nutrition standards/guidelines that are in place to implement specific policies or programmes within public sector basic schools in Ghana. First, it will evaluate the nutritional quality of foods and beverages sold or provided in basic schools. Second, the project will assess the capacity and readiness of community stakeholders to accept and implement interventions to improve food environments of children in Ghana. Third, the activities of the project to build capacity on food environment research in the region, and engagement with stakeholders with a potential to influence policy, are described.

### Conceptual Foundations and Justification for the Study

The study was stimulated by data from the recent efforts of the authors at benchmarking food environment policies in sub-Saharan Africa, which began in Ghana (23), as well as the established link between obesogenic food environments and NCDs. Several recent epidemiological studies and meta-analyses associate unhealthy food environments with unhealthy diets, obesity, and NCDs risk across the lifespan. These studies indicate that the consumption of ultra-processed foods (e.g., carbonated drinks, sweet/savory packaged snacks, SSBs, chocolates), is associated with increased risk of obesity and associated NCDs (38–41). The benchmarking of food environment policies in Ghana indicated that a great opportunity exists to effectively respond to NR-NCDs. By interactions with local policy-makers through the Food-EPI process, it was found that tackling unhealthy food promotion and improving food provision would be the most likely actions to address the problem of obesity and NCDs risk factors, especially in children and adolescents (23).

Food provisioning and promotion/marketing have been shown to influence what people choose to eat (whether healthy or unhealthy). Food promotion affects food preferences and purchase behavior, purchase requests (of children), and consumption habits (42–49). Both food promotion and food provision reinforce social norms that unhealthy foods are acceptable and desirable (36). With continued exposure to promotion, the brain is reprogrammed to react to cues about the food (sight, smell, thought, suggestion)–producing cravings to influence preferences, choices, and, ultimately, consumption or overconsumption of unhealthy foods (50, 51). The MEALS4NCDs prevention project draws on the science of food choice, social practices of dietary intake, and counter-marketing approaches (deliberate efforts to market more healthier food products than unhealthy ones) to understand how food provisioning and promotion environments influence food choices, food intake, and thus, health outcomes. It has been shown elsewhere that exposing children to advertisements for nutritious foods positively improved attitudes and beliefs toward them (19, 23). To understand how food provisioning and promotion environments influence food choices, food intake, and thus, health outcomes.

### Project Work Packages and Logic Model

**Figure 1:**
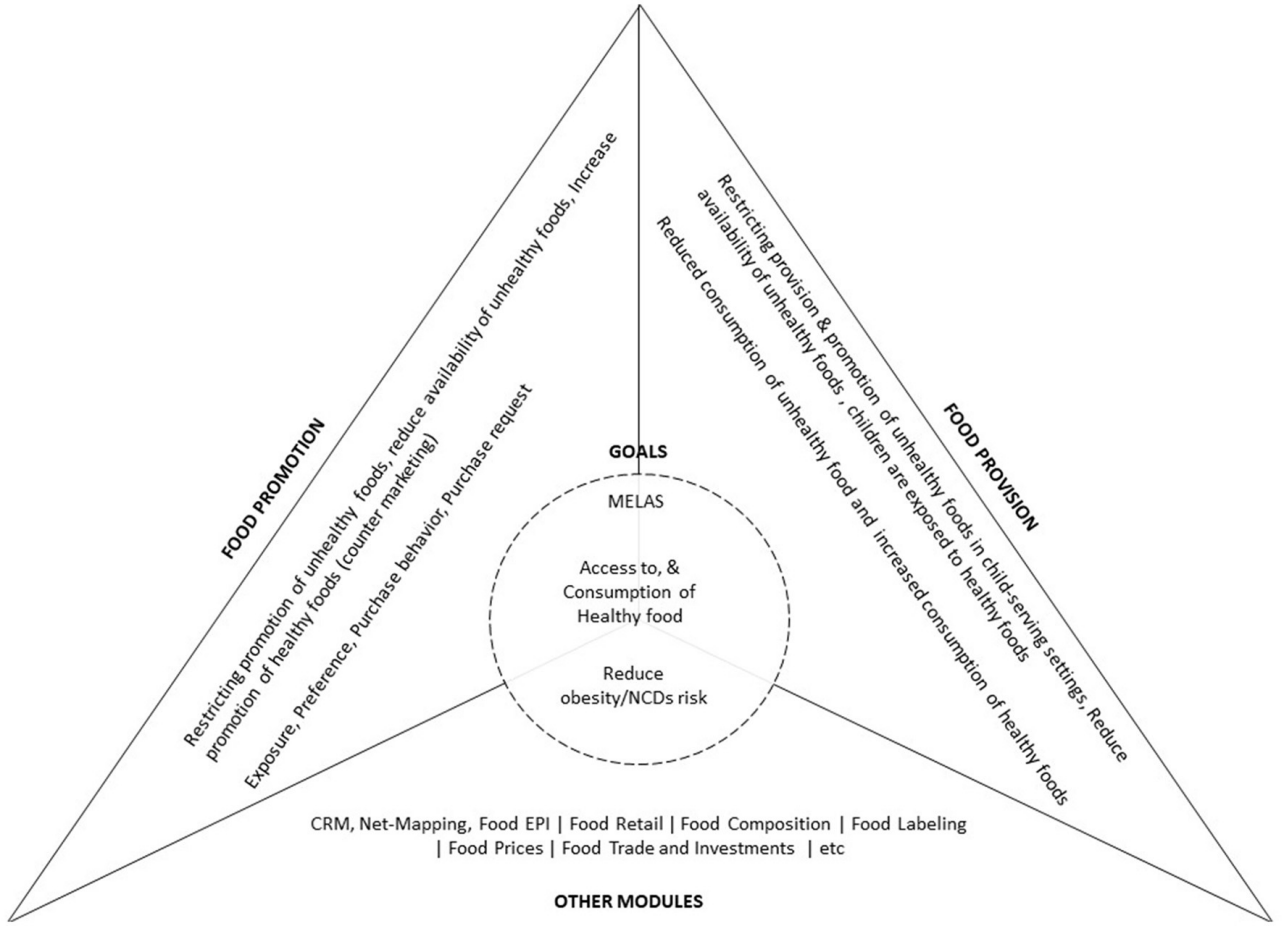
Conceptual framework for the MEALS4NCDs project. The top two triangles (to the left, and right) depict the INFORMAS-related work packages to be deployed in the study, their respective approaches and desired outcomes. The base triangle depicts INFORMAS modules already completed (Food-EPI), non-INFORMAS approaches (CRM, and Net-Mapping), and modules to be implemented in future. Implementation of all work packages contribute to the MEALS4NCDs desired short-term goals (adaptation and deployment of the modules–within 3 years); and long-term goals (institutionalization efforts, and as listed in the circle to be achieved after the 3-year project period).

The project will be delivered through three interlinked work packages (WPs) over a period of three years: WP1-Food Promotion, WP2-Food Provision, and WP3-Community Readiness WP1 and WP2 derive from existing INFORMAS Modules. WP1 Food Promotion assesses the exposure and power of promotion of unhealthy foods and non-alcoholic beverages to different population groups. WP2-Food Provision generates data to answer the question: “What is the nutritional quality of foods and non-alcoholic beverages provided in different settings (e.g., schools, hospitals, workplaces)?” Focusing on public basic schools in Ghana, this project will explore the socio-cultural and political contexts (in terms of policies, guidelines, laws, culture, social environment). It will also study the extent of programme/policy implementation, policy enforcement, compliance with existing guidelines and policies in basic schools, as well as how school pupils interact with their environment (in terms of food marketing, acquisition, provisioning, sale/retailing, and consumption). WP3-Community Readiness, not one of the INFORMAS Modules, will explore the readiness of school/education stakeholders to accept and implement interventions to improve the food environment of children in selected settings in Ghana (see Figure 1). While the time allotted to the direct implementation of the work packages is “3 years,” the proposed institutionalization efforts (mainstreaming a food environment monitoring system into Ghana public health delivery systems–to regularly gather and disseminate information to key factors such as policy-makers, government, programme implementers, civil society, private sector, and community) is envisaged to continue beyond the life of this project.

The logic model derives from a recent realist review focused on facilitating healthy food consumption in lower-middle income African countries (37). Thus, we hypothesize that if governments introduce policy measures that restrict the marketing of unhealthy foods, their production, processing, importation, and marketing will reduce–leading to decreased consumption. Similarly, if relevant food provision policies (52) are implemented in schools and early childhood education services for food service activities (canteens, food at events, promotions, vending machines, etc.) to provide and promote healthy food or to expose children to higher proportion of healthy foods (e.g., fruit/vegetables) compared with unhealthy foods, this exposure will have a positive impact on dietary behaviors.

Recognizing that implementing the three WPs may not be sufficient to improve the food environment, the project seeks to examine other actions – the regulatory, legislative, and fiscal levers of food environment policy (see “Other modules” in Figure 1). For instance, the project will explore opportunities to strengthen the capacity of food environment researchers and public sector actors to plan and implement actions in sub-Saharan Africa–in line with existing global and local calls for action (32, 53–55).

### Project Partners

The project partners comprise four groups of stakeholders. The research team (comprising project investigators and early career researchers – a multidisciplinary team of local, and international experts/health policy-makers from seven institutions located in Ghana, Kenya, Belgium/New Zealand, The Netherlands, Canada, and the UK/France); the project advisory board, and various local implementation partners (civil society organizations, governmental agencies, lawmakers, United Nations agencies). The Advisory Board members and the implementation partners were selected based on their expertise and experience in food environments, based on the potential to offer guidance and insights about local, regional contexts – both in terms of technical inputs, and policy-making influence.

## Methods

**Figure 2:**
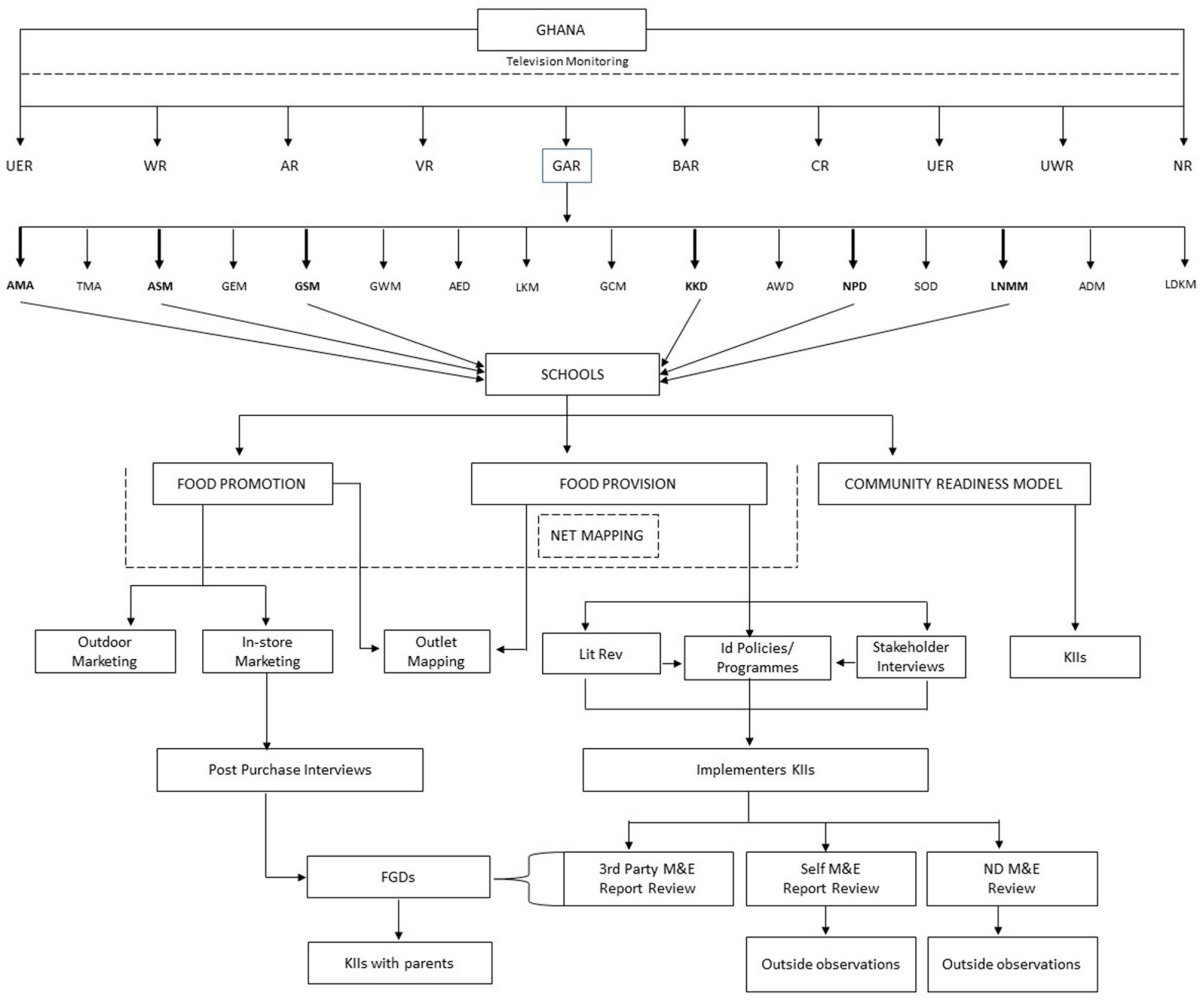
Overall study organization and sampling schema. AED, Ada East District, ADM, Adentan Municipality; AMA, Accra Metropolitan Assembly; AR, Ashanti Region; ASM, Ashaiman Municipality; AWD, Ada West District; BAR, Brong Ahafo region; CR, Central Region; ER, Eastern Region; GCM, Ga Central Municipality; GEM, Ga East Municipality; Ga South Municipality; Ga West Municipality; GAR, Greater Accra Region; KKD, Kpone- Katamanso District; La Dade Kotopon Municipality; La Nkwantanang-Madina Municipality; LKM, Ledzokuku-Krowor Municipality; NPD, Ningo Prampram District; NR, Northern Region; Shai Doku District; TMA, Terna Metropolitan Assembly; UER, Upper East Region; UWR, Upper West Region; VR, Volta Region; WR, Western Region. The figure summarizes the organization of the study including its work packages and data collection approaches. From top: TV monitoring covers all 10 regions of Ghana; the GAR was selected for the field work; 6 districts of the GAR were selected. Two hundred schools were selected from the 6 districts for the study. The 3 WPs (Food promotion, Food provision, and CRM) were implemented in all 6 districts. The Net-Mapping exercise (embedded in CRM) focused on food promotion, and provision. The various approaches associated with these 3 WPs include outdoor marketing, KIIs, exit interviews, FGDs. The figure also depicts the stepwise approach to compiling, describing, and evaluating existing nutrition policies–L'Abbe et al. (56). Step 1–identifies policies through literature search and stakeholder interviews; Step 2–describes characteristics of policies identified in step 1. Step 3–further unpacks specifics/details of standards applicable to the identified policies. Component 2 of the L' Abbe et al.'s framework entails interviews with programme implementers, review of M&E reports where available, and onsite observations).

In the implementation of the project, we will adopt a multi-method cross-sectional study design that applies quantitative and qualitative methods. The various methods to be deployed are described under field procedures (and are summarized pictorially in Figure 2).

### Study Tool Development and Adaptation Processes

The protocol for this project was developed iteratively and collaboratively by project partners. A series of virtual engagements between the project team and leaders of the adapted INFORMAS Modules led to the development of initial drafts of the protocols. Following this, a face-to-face protocol adaptation workshop, facilitated by project co-investigators and Module Leaders was conducted in August 2019 at Aburi, Ghana. Three work teams (each team focusing on one of the work packages) were assigned the task of revising the initial draft protocol. WP1-Food Provision received additional inputs from the INFORMAS School Health Group (part of the wider INFORMAS Network). The CRM component (which is not part of INFORMAS methods) adapted an existing Net-Map interview tool (57) and CRM protocol (58), building on the previous use of CRM in Ghana in deprived communities (59). The various modifications/adaptations made to the original protocols are highlighted in the Supplementary Material (Appendix 16). The final MEALS4NCDs protocol comprised 15 tools. Altogether, the INFORMAS protocols contributed significantly to creating a project protocol that is tailored to the Ghanaian context. Details of the field procedures are outlined below.

### Field Procedures

#### Television (TV) Advertisement Monitoring (Nationwide)

The following sections describe the planned implementation of TV advertisement monitoring.

#### Selecting TV Channels, Duration of Monitoring, and Days of the Week to Record

Sampling of TV programming will be conducted in three stages: 1. Selection of TV channels to monitor; 2. Duration of monitoring (months, days, and hours to be recorded); and 3. Days of the week to record. Ghanaian TV services includes free-to-air (analog and digital services) and paid satellite. By the end of June 2017, there were 51 free-to-air TV channels in Ghana; all channels have some time allocated for programmes for children (60). No data are available on child TV viewing audiences in Ghana. As such, we plan to consult with experts in the Ghana media industry, namely, the media measurement service provider – GeoPoll (60) to ensure that we select the most appropriate channels. Channels that do not broadcast advertisements will be excluded. Also, pay-to-air TV channels will be excluded because of low household subscription (61). At a minimum, the top three free-to-air local/national television channels, in terms of children viewership, will be included in the data collection process.

The data collection period will cover one school term (3 months) and a month of vacation/recess. National holidays and special events will be excluded to ensure that the data represent typical broadcasting. For each advertisement broadcast during the sampled period, details will be collected on brand and products, channel, time shown, nature of the product (food, non-food), and food type. All programming for selected days will be recorded from 6:00 am through to 00:00 am (midnight).

In the final stage of sampling, we will adopt the composite week sampling method to identify the days of the week to be monitored. The studies of Riffe et al. (62) and Laar (63) show that this sampling approach is consistent with the stratified sampling design requirement of INFORMAS of a minimum of 4 days in both the working week and at weekends. Thus, the content of the three TV channels will be recorded as follows: a random start date for TV Channel 1 – Sunday for week 1, Monday will be recorded for week 2; Tuesday will be recorded for week 3, Wednesday for week 4, etc. The other two TV channels will be recorded on the same days as the first to avoid any differences in advertising patterns between weekdays and weekends. In the event that a sampled day is not eligible (e.g., national holiday or special event), the next available date is included. Irrespective of start day/date, efforts will be made to ensure that a minimum number of 4 weekdays and 4 weekend days will be recorded.

#### Recording Data

Data will be collected manually by recording live television programmes as per existing protocols (44, 64). All programming will be recorded onto external hard drives. The recorded data will be coded using a coding sheet (Appendix 1 in Supplementary Material).

### Assessments at the Sub-national Levels

#### Sampling Study Districts and Child-Serving Institutions (Public Sector Basic Schools)

A multistage sampling approach will be employed to select the geographic region of the study, districts, and the main primary sampling units (PSUs) – public basic schools. The first stage of the sampling process will entail purposive selection of a geographic region. Of 10 regions in total, Greater Accra, which hosts the national capital, was/and remains the most urbanized and most marketed to the region of Ghana, will be purposively chosen. The region is sub-divided into 16 administrative districts categorized as Metropolitan, Municipal, and Districts Assemblies (MMDAs). A representative sample of six districts will be selected using both probabilistic and non-probabilistic sampling approaches, as detailed below.

To select the six administrative districts, the 16 districts will be grouped into three strata/sub-administrative units of districts, municipalities, and metropolises. This administrative categorization is a marker of urbanization status and gives an idea of district-level socio-economic deprivation. Of the 16, two are metropolises with a comparable poverty head count (the proportion of population living below the national poverty line). The most urbanized will be selected. Nine are municipalities (three will be randomly selected after further stratification by poverty headcount); and five are districts (two will be selected using the same criteria as in the selection of municipalities).

With an overall sample of 200 schools, the required number of basic schools in each of the six districts will be selected using a probability proportional to size, with size of the school defined as the number of pupils in the school. Schools will be stratified by location (rural or urban), level (primary, or junior high), and sorted within each stratum based on roll size data from the Ghana Education Service. Following this, systematic random sampling will be performed in their selection. According to the records of the Education Management Information System (EMIS) of the Ghana Education Service, the Greater Accra region had a total of 862 public primary schools and 812 public junior high schools in 2018 (65). Sampling 200 schools will give us 80% statistical power to compare a hypothesized outdoor unhealthy food marketing rate of 47% with the recently observed rate of 57% (21). In calculating the sample size for this aspect of the study, we hypothesized that the rate of outdoor marketing of unhealthy food will be 47% in comparison with a previously observed rate of 57% (21). Whereas, the study by Amevinya et al. (21) was conducted in a highly urbanized university setting, this study would involve six districts (which include urban, peri-urban, and rural areas). Rates of advertisements are usually lower in less urban settings.

#### Mapping and Assessing Outdoor Advertising in and Around Selected Schools

All outdoor adverts in stipulated areas surrounding all the sampled schools will be observed and recorded manually, using a camera and geo-positioning systems-enabled tablets. The stipulated area will be defined using a walkable road network distance from the entrance of the school. A walkable distance is herein defined as limited by 250 m road network boundaries, within which pupils are able to purchase from food vendors during short breaks. To implement this, research staff (working in pairs) will walk and observe the area within the established perimeter, around the sampled schools, to identify and record coordinates as well as take pictures of all advertisements (food and non-food adverts). All captured food advertisements will be coded using a predesigned coding sheet (see Appendix 2 in Supplementary Material). In cases where multiple advertisements are included per picture, the type of advertisement, type of food, as well as the brand will be the focus in the photographed image. Since advertisements in outlet windows count as outdoor advertising, we will capture all such advertisements as associated with both mobile/ephemeral and non-mobile outlets.

#### Mapping Food Provision and Food Retail Outlets Within Stipulated School Zones

At the same time as the outdoor advertising assessment, we will conduct a census of all food retail outlets (a place/structure used primarily for the preparation and sale of food and non-alcoholic beverages mainly for consumption off the premises) and food provision outlets (a place/structure used for the preparation, sale, and serving of ready-to-eat food to customers) within the immediate school compound using a road network distance of 250 m from the school entrance. This mapping exercise will geo-locate all kinds of outlets categorized as “supermarkets; school canteens; shops; kiosks; vegetable/fruit/food stands/table top outlets; local vendors; restaurants; chop bars; cold stores; open markets; bakery; drinking bars/pubs” – as in Green et al. (66). All such outlets may be operated as completely self-service; both self- and assisted-service and full-assisted service. In addition, the name of the outlet (if available) will be recorded, and a picture will be taken. The unique foods available will be assessed and categorized using the NOVA food classification system (67) or the core, non-core, miscellaneous approach by INFORMAS (64). The experience implementing similar exercises reveal a phenomenon of shared outlets/vending spaces and the tendency for outlets to appear and disappear at certain times of the day (22). Therefore, efforts will be made to map fixed, mobile, and non-mobile yet ephemeral outlets (see Appendix 3 in Supplementary Material).

#### Conducting In-store/Supermarket Assessment and Post-purchase Exit Interview

The in-store assessment of promotional strategies will cover all eligible supermarkets in the selected districts. In this study, a supermarket is defined as a self-service food retail outlet with accessible aisles to customers and has at least a staffed checkout aisle or a cash register. The research team will visit the supermarket and record all promotional strategies, e.g., audiovisuals (music, loudspeakers, screens displaying promotional characters), product placement, shelf heights, premium offers (the assessment tool is included as Appendix 4 in Supplementary Material).

Supermarket/in-store promotional assessment will be implemented simultaneously with the administration of a short exit survey (see Appendix 5 in Supplementary Material), which will be administered to eligible adult customers exiting from shopping from the identified vendor. Shoppers will be eligible if they are parents/guardians with a child(ren) aged 6–17 years and have bought at least a food/beverage product from the supermarket. A consecutive sample of 10 interviews per supermarket is expected. The short survey will assess overall shopping experience in the store, what was purchased, as well as promotional activities they encountered at the supermarket. In addition, relevant questions from the Green and Glanz (68) perceived nutrition environment measures survey will be fielded. As part of the consent process (for all aspects of the study), interviews will only be taken after valid informed consent has been received from the vendor at the food outlet. The researchers will need to gain additional consent *via* a photo release form if they cannot obtain a photograph without the vendor appearing in the photo. Similarly, post purchase/exit interviews of supermarket users required *a priori* consent by eligible adults.

#### Food Provision Policy, Programme, and Practice Assessment and Monitoring

Per existing INFORMAS protocols, the exercise will aim to, first, describe the nutrition standards or guidelines that are in place to implement specific policies or programmes within public basic schools in Ghana. Second, it will evaluate the nutritional quality of the foods and beverages sold by vendors (e.g., in school cafeterias and canteens) or provided (e.g., foods provided by school feeding programmes) in these settings. In addition, the study will explore key considerations in making decisions around what foods are provided in such settings by engaging relevant stakeholders. We will adopt the L'Abbe et al. (56) two-component framework.

Briefly, Component I will involve compilation, description, and evaluation of any existing nutrition policies/programmes using the expanded INFORMAS step-wise approach. This component will involve three key steps (steps are depicted pictorially in Figure 2).

Step 1 will identify food provision policies and programmes through a literature search and stakeholder interviews. Initial policy/programme identification will rely on the recent Food-EPI exercise (23), complemented with interviews with relevant stakeholders – using Appendix 6 in Supplementary Material.

Step 2 will describe the characteristics of the food provision policies and programmes identified in step 1. The core characteristics to be identified will include the jurisdiction of the policy/programme, availability of guidelines for the policy/programme, geographical coverage of the policy/programme, school sector coverage of the policy/programme, and type of nutrition standards/guidelines associated with the policies/programmes. This will be implemented using a checklist of ideal characteristics of a school feeding policy/programme [see (56)].

Step 3 is dedicated to unpacking details of standards/guidelines applied to foods/beverages that are provided and/or sold in the identified policies/programmes and described in steps 1 and 2. For instance, step 3 will determine the basis of any applied standard (i.e., whether the standard is applied per serving or per 100 g of food). With the help of a policy-rating tool (see Appendix 7 in Supplementary Material), we will describe the existing policies/programmes and their associated standards/guidelines, and evaluate them against ideal benchmarks for school food policies/programmes.

Component II entails monitoring the implementation of food provision policies/programmes and will involve three key steps as detailed in L'Abbe et al. (56). It will be implemented using four separate study tools (see Appendices 8, 9, 10, and 11 in Supplementary Material). The purpose of this component is to evaluate the nutritional quality of the foods/beverages sold or provided to children in these settings, relative to the nutritional standards/guidelines in any identified nutrition policy/programme. As many schools in Ghana run a system that includes both foods sold on the school compound and food provided by school canteens/commercial food service canteens, we propose to conduct the evaluation of foods sold separately from foods provided to children. Here, nutritional quality refers to compliance with nutritional standards/guidelines applied in the rated “strong” policies/programmes. Based on anecdotes and the collective lived experiences–at the time of the study, we hypothesized that in many of the schools, nutritional quality assessment will not be conducted as part of the monitoring of the school food/nutrition programme, as such we will conduct interviews with school heads, administrators, and/or food service providers such as head teachers, caterers, cooks and food service vendors. Second, in the event that there are no nutrition standards/guidelines to guide the implementation of school food/nutrition programme, we will conduct the assessment of the nutritional quality of the provided and/or sold foods relative to the standards applied in the Core/Non-Core food classification by INFORMAS (64), the NOVA classification (67), or a locally generated classification (69).

#### Focus Groups Discussions (FGD) With School Children and Interviews With Their Caregivers

In total, 18 focus groups discussions will be conducted (three per district) with children (12–17 years). Each will comprise a maximum variation sample of 8–12 school children, selected with consideration given to gender, age, location, level of education (primary or junior high), and socio-economic status). The focus groups will explore the nutritional knowledge and perspective of children on what a healthy diet is, their typical eating habits (what they eat on a regular day–weekends/weekdays), changes in diet over time, awareness of school nutrition policies/programmes, their perspectives on promoting healthy food (counter marketing), views on food advertisements, in general, media influences on food choices, general recall of features of advertised product, and brand recall (see Appendix 12 in Supplementary Material). The parents/caregivers of children who participate in the FGDs will be interviewed. The interviews will cover parental appreciation of school food environment of children, TV influences on nutrition and health of children, etc. (Appendix 13 in Supplementary Material).

#### Implementation of Community Readiness Mapping (CRM)

Community readiness mapping will be implemented to gauge the readiness of and capacity of the studied community/stakeholders, as well as barriers and facilitators to implement food environment improvement interventions, which is not included in the INFORMAS protocol. The CRM serves as a theoretical framework for understanding and improving community readiness for interventions (70). Limited research has been conducted in African countries using the CRM. For example, it has been applied in Mali (71), South Africa (72), and, recently, in Ghana (59). In this study, the CRM will aim to answer the question of how ready the community is to accept and implement interventions to improve the food environments of children in the Greater Accra region of Ghana. The assessment will focus primarily on aspects of the food environment related to food marketing and the availability of unhealthy foods in and around basic schools. Here, food environments of unhealthy children refer to “food environments that promote the consumption of unhealthy food items, such as processed meats; sugar and sweet spreads; cakes and sweets; sodas and sweetened beverages; savory snacks; fried foods; and condiments high in sugar or salt.” It can also include food that is not safe to eat, because it is unhygienic or contaminated. “The community” is operationally defined both over geographic space (geographic community) and according to stakeholders of interest (interest community). Geographically, the focus will be the six sub-communities of districts of the Greater Accra region of Ghana. The interest community will comprise individuals/key informants from both within and outside of the geographic community, adjudged to wield influence and/or play a leadership role in promoting healthy food environments. It is expected that the selected key informants will be able to provide in-depth viewpoints on existing actions to improve food environments of children and discuss their barriers and facilitators.

Individual community readiness mapping informants will be selected purposively to represent a wide range of sectors/programmes (regulatory authorities, schools, commerce, health (e.g., MoH, GHS), religious institutions, school feeding programmes, children/youth clubs, urban planners) as well as policy areas in question – TV marketing, outdoor marketing, food provision, etc. The actual list will be compiled with inputs from the study team and local people interviewed using Appendix 14 in Supplementary Material. To ensure that a maximum variation sample of key informants participate in the exercise, the CRM exercise will begin with stakeholder mapping using the IFPRI Net-Map methodology (73). Two to three key informants will be identified per sector. We will be measuring the overall readiness of the community. At a minimum, 6–12 interviews will be conducted (actual number will be determined following the Net-Mapping process). By adapting an existing Net-Map interview tool (57) (see Appendix 15 in Supplementary Material), participants will be asked to indicate, “Who are the stakeholders that influence children/food environment and their dietary behavior, at the National level?” This question will then be followed by another question, “how do the identified actors interact with each other?” The list that will be generated will be placed on a large sheet of drawing paper and with the help of participants, and links will be drawn using the markers. Appropriate map generation tools (such as large drawing paper and differently colored markers) for drawing the map will be provided for the process. Once the links have been established, determination of their relative influence in the food environment will be made by asking, “how influential each actor is in promoting school food environment.”

### Quality Assurance

Data quality assurance measures will be instituted pre-, during, and post data collection – including the identification of qualified field staff, rigorous training of field staff, and pretesting of study tools.

For instance, during the pre-test, two principal researchers and all research assistants would code an identical area around a site independently. Their respective coding would then be compared, and inter-rater reliability (IRR) would be computed using the formula:

$$\begin{array}{l} \text{IRR}=\;\frac{\text{Number of agreements}}{\text{Number of agreements }+\text{ Number of disagreements}}\;\times \;100 \end{array}$$

The INFORMAS protocols we have adapted recommend a minimum of 80% inter-coder reliability. In the event that this minimum level is not achieved, coding discrepancies would be discussed, and further training would be given to research assistants, if required.

Similarly, for TV monitoring data, the raw data (recorded videos) will be coded by trained members of the team with the help of the study-specific coding sheet, as per the INFORMAS protocol (64). Inter-coder reliability will be computed as follows: a random sample of 10% of all coded images will be selected and coded by an independent researcher. Individual scores for each attribute (all categorical variables) will be compared using Cohen's kappa statistic (κ).

Lessons from the pretest will inform modifications and finalization of the tools. There will be no pilot study, as lessons from recently implemented studies (21, 22) provide sufficient guidance on sample size calculations, community entry, and field etiquette.

### Data Analyses

Different approaches will be deployed to analyse data from this study. We describe below how the pooled quantitative, qualitative, policy assessment, and GIS data will be analyzed. Table 1 shows a summary of various indicators that will be generated as part of this project.

**Table 1:** Study outcome indicators by tool.

**Data collection instrument**	**Study outcomes/indicators**
Appendix 1. TV monitoring advert coding sheet	▪ Mean rate or frequency of advertisements per channel per hour ▪ Mean rate or frequency of food vs. non-food advertisements per channel per hour ▪ Mean rate or frequency of unhealthy vs. healthy food ads (core vs. noncore) advertisements per channel per hour ▪ Mean rate or frequency of unhealthy (non-core) food groups ▪ Mean rate of (healthy vs. unhealthy / core vs. non-core) food advertisements per channel per hour with promotional persuasive promotional techniques per unit ▪ Ratio of healthy to unhealthy advertisements ▪ Number of healthy vs. unhealthy advertisements ▪ Proportion of food advertisements by major food categories ▪ Mean rate of promotional characters food vs. non-food per channel per hour ▪ Mean rate of promotional characters (food vs. non-food; unhealthy vs. healthy food) ▪ Mean rate of premiums (food vs. non-food; unhealthy vs. healthy food) ▪ Proportion of food advertisements by major food categories
Appendix 2. Outdoor advertising assessment tool	▪ Rate of total food and non-core (and/or unhealthy) food advertisements per 100 m^2^ ▪ Rate of total food and non-core (and/or unhealthy) food advertising by size of advertisement per 100 m^2^ ▪ Rates of advertising per 100 m^2^ in high and low density population areas ▪ Mean rate of (healthy vs. unhealthy/core vs. non-core) food advertisements with promotional persuasive promotional techniques ▪ Mean rate of (healthy vs. unhealthy/core vs. non-core) food advertisements with premium offers ▪ Proportion of food advertisements by major food categories
Appendix 3. Tool for mapping food provision and retail outlets	▪ Geo-location of all food outlets within the immediate school compound and within road network buffers of 250 m from the main entrance of all selected schools ▪ Census of all food provision and food retail outlets within the immediate school compound and within road network buffers of 250 m from the main entrance of all selected schools.
Appendix 4. In-store marketing assessment tool	▪ In-store marketing/promotional strategies taking place in Supermarkets ▪ Total healthy food shelf space covered ▪ Total unhealthy food shelf space covered ▪ Relative availability (cumulative shelf space covered) of healthy vs. unhealthy foods ▪ Total healthy food varieties recorded ▪ Total unhealthy food varieties recorded ▪ Ratio of total availability (variety) of healthy foods vs. total availability of unhealthy foods in-store. ▪ Ratios of availability of a selection of healthy food groups vs. availability of a selection of unhealthy food groups in-store
Appendix 5. Exit interview or post-purchase survey	▪ Overall consumers' instore shopping experience
Appendix 6. Tool for identifying food provisioning policies and programmes	▪ Identify existing nutrition policies/programmes ▪ Describe the characteristics of any identified nutrition policies/programmes
Appendix 7. Policy rating tool	▪ Obtain an objective assessment of whether or not food provision meet set existing policies/programmes standards/guidelines ▪ Evaluate existing nutrition policies/programmes against ideal benchmarks for school food policies/programmes.
Appendix 8. IDI guide for school heads and administrators	▪ Monitor implementation of food provision policies/programmes in schools
Appendix 9. IDI guide for caterers	▪ Monitor implementation of food provision policies/programmes in schools ▪ Evaluate the nutritional quality of the foods/beverages provided to children in these settings, relative to the nutritional standards/guidelines ▪ Observe compliance with nutritional standards/guidelines
Appendix 10. M & E review tool	▪ Monitoring implementation of food provision policies/programmes
Appendix 11. Onsite observations and compliance assessment tool	▪ Monitor implementation of food provision policies/programmes in schools ▪ Evaluate the nutritional quality of the foods/beverages provided to children in these settings, relative to the nutritional standards/guidelines ▪ Observe compliance with nutritional standards/guidelines
Appendix 12. Qualitative focus group discussion guide	▪ Children's nutrition knowledge and perspective on what is a healthy diet ▪ Children's typical eating habits ▪ Children's awareness of school nutrition policies/programmes, and satisfaction with school food provided and/or sold ▪ Children's perspectives on promoting healthy food (counter marketing) ▪ Children's views on food adverts and it influences on food choices
Appendix 13. Qualitative KII guide for parents	▪ Parents' appreciation of their children's school food environment, TV influences on their children nutrition and health
Appendix 14. CRM Key Informant tool	▪ Community readiness to accept and implement interventions to improve food environment in the Greater Accra region of Ghana ▪ Community leaders' in-depth viewpoints on existing actions and how to improve children's food environments ▪ Barriers and facilitators in improving children's food environments
Appendix 15. Net-Mapping tool	▪ Map of key actors closely engaged with or have influence in nutrition, health, and food environment (particularly for children) in Ghana ▪ Identified actors interact with each other

#### Analysis of Quantitative Data

Advertising, publicity, and sales promotion of food to children *via* television, and outdoor and indoor advertising within school zones will be analyzed strictly as per the protocols of INFORMAS (44). For each school, two kinds of data will be generated: one related to the outlets observed, and the other to the food items provided or sold. The density of the outlets will be calculated per 100 m^2^ to enable standardized comparisons within the 250-m road network distance.

For in-store marketing assessment, we will present frequencies of all promotional strategies taking place in these outlets/stores such as audiovisuals (music, loudspeakers, screens displaying promotional characters), spatial distribution, and product placement. Pictures of unique foods will be assessed and categorized using the NOVA system (67) and/or the core/non-core, food-based approach developed by INFORMAS (64). The frequency tabulation of post-shopping exit interview data details the items purchased; overall shopping experience in the store, whether they did prior planning for the shopping at home or whether they bought any food or drink not previously contemplated; and the promotions they remember from the store. At the school level, frequencies of healthy food/beverage availability will be generated and compared by school level (primary or junior high school) and district (district, municipality, metro). Ratios of healthy food outlets to total food outlets will be calculated for each provision outlet based on the total counts of each. Furthermore, bivariate and multivariable analyses will be performed using appropriate approaches.

#### Analysis of GIS Data

The ArcGIS desktop software will be used to analyse spatial data and generate maps. The availability of various categories of products/advertisements will be compared cartographically. Thus, the total number of food outlets and advertisement within 250 and 100-m distances around rounds in each school will be mapped. The maps will be produced to help understand how the food environment varies spatially in the study locations. Also, to be mapped for each district and for pooled data, will be the descriptive data of frequency of food/beverage advertisements, size of food advertisement, setting of advertisements, type of advertisement, product type, and location of advertisements or food provided/sold within the schools. The geographically weighted regression (GWR) technique will be used to model the local relationships between the predictor factors (e.g., rurality/urbanity, demographic factors; physical environment characteristics, type of school, roll size (number of student in the school), district type, poverty incidence, etc.) and the outcomes of interest–intensity of outlets/advertisements within a 500-m radius.

#### Analysis of Qualitative Data

Audio recordings of FGDs, KIIs, as well as IDIs will be transcribed verbatim. Transcriptions will be augmented with field notes of the researchers. The data resulting from the transcriptions will be evaluated and coded using the constant comparative method of theme generation. Qualitative software (e.g., Nvivo SQ) will be used to assist with data storage and management, such as development of data files, codes, codebooks, themes, and categories. The CRM-specific transcripts will be scored by two independent scorers within the research team using nine anchored rating statements for each dimension (74). Data reporting will be undertaken according to the consolidated criteria for reporting qualitative research (COREQ) (75). To maintain confidentiality, all names will be reported as pseudonyms, and identifying details will be removed.

Data from the Net-Map (including the photographs, notes, and transcripts), on the list of actors (using actor identification codes), links between actors, and relative influence will be entered into Microsoft Excel (as two separate sheets: 1. List of individual actors and 2. List of links connecting all actors). Actor identification codes and actor categories (the broad sectors that actors belong to) will be recoded for consistency across link list and actor list. The two excel sheets will be imported into Gephi, social network analysis software. All analyses (statistics, filtering, network image generation, and visualization) and image capturing will be conducted in the overview and preview features of Gephi. Overall, link-specific network statistics (weighted average degrees, network diameter, graph density, modularity, and connectedness components) will be computed to determine network characteristics. Network visualization images will be weighted by actor nodes using relative influence. The Yifan Hu algorithm (76) will be used to visualize network images.

Overall, data reporting will be undertaken according to the Strengthening the Reporting of Observational Studies in Epidemiology (STROBE) statement: guidelines for reporting observational studies (77).

### Stakeholder Validation, Dissemination, and Knowledge Translation

The results from this study will be shared during a validation workshop to open to all the MEALS4NCD stakeholders. We will adopt the “constructive confrontation design” (78). It is envisaged that the process will lead to an enhanced collaborative conversation on the initial findings, critique of, and possible re-interpretation of the results. Also, draft policy briefs will be tabled for inputs during the validation workshop. We believe that the views of multiple stakeholders will strengthen the policy briefs.

The proposed strategies for dissemination include standard dissemination activities such as publishing peer-reviewed manuscripts (anticipate *n* = 10 in reputable open-access journals) and presentation at local or global scientific conferences (*n* = 6). We will also create an open-access project website, generate and disseminate project reports, produce policy briefs, and conduct consultative meetings/dissemination workshops. Additional efforts to disseminate study data more widely and to support its ultimate application may entail packaging of the research findings into user-friendly formats, e.g., press releases and research briefs, guided by the research findings. We will also run “building project findings into policy and practice consultative meetings” (*n* = 4). This will entail several consultative meetings with diverse but relevant stakeholders, namely, government ministries, departments, international organizations such as the WHO, FAO, and UNICEF, as well as civil society groups. Among others, these meetings will deliberate on the strategies for feeding the findings of the project into policies, practice, and scale up. We will produce policy briefs (*n* = 2 covering food promotion and provision) to facilitate a dialogue with these stakeholders.

## Anticipated Results and Discussion

We have described the rationale and protocol of the MEALS4NCDs project, which aims to measure and support public sector actions that create healthy food marketing, retail, and provisioning environments for Ghanaian children. The study builds on the recent efforts of the authors at introducing the INFORMAS approaches to Africa (23). The first to innovatively integrate local community participatory approaches such as the community readiness model (CRM) and Net-Mapping into the INFORMAS approaches in sub-Saharan Africa, this study will facilitate the appreciation of the processes through which the INFORMAS approaches are actualized in Africa.

The overall hypothesis of the MEALS4NCDs project is that providing measurement, evaluation, accountability, and leadership support to governments and other stakeholders will facilitate the introduction of comprehensive and strong policy measures (52) that serve to regulate the promotion of unhealthy foods or the implementation of clear, consistent food environment policies in schools and other child-serving settings. By engaging stakeholders and generating evidence that reiterates, illustrates, and confirms the association between implementation of policies and improvement of the food environment, citizens, national policy-makers, government officials, civil society, and industry stakeholders may find utility to the data and act in partnership.

A measure of the longer-term success of the project would be incorporation into national policy and practice, recommended interventions, and priority actions. Locally, the ultimate goal of the project is the establishment and institutionalization of a food environment monitoring system into the Ghana public health delivery systems in ways that regularly gather and disseminate information to key actors (policy-makers, government, programme implementers, civil society, private sector, community) for action. Regionally, we will leverage additional funding to facilitate the seamless implementation and contextualization of other INFORMAS modules, such as the Food Composition, Food Prices, and Food Labeling. In this regard, although challenges in leveraging the needed resources is a potential limitation, the planned capacity building initiatives during this phase of the project may sustain the project vision well-beyond the life of the current funding. The capacity building initiatives embedded in the project, e.g., the formation of Africa Food Environment Research Network (FERN) will instigate interest and mobilize the critical mass of experts to support this regional scaling up efforts. Globally, the project is specifically tailored to addressing the goal of the International Development Research Centre (IDRC, Canada) of improving food environment and, therefore, health, and contributing to the ambitions of other international organizations, e.g., the WHO Global Action Plan 2013–2023 on NCDs, the WHO NCDs Progress Monitoring of Member Countries, and the United Nations Sustainable Development Goals (SDGs). With respect to impacting current and future food environment policies, the current cross-sectional data may appear as a limitation; however, an effort to have repeated surveys will generate relevant data policy-influencing data.

## Data Availability Statement

The original contributions presented in the study are included in the article/Supplementary Material, further inquiries can be directed to the corresponding author.

## Ethics Statement

The studies involving human participants were reviewed and approved by Ethics Review Committee of the Humanities, University of Ghana (Approval # ECH 152-18-19), and the Ghana Health Service Ethical Review Committee (Approval # GHS-ERC 005-06-19). Written informed consent to participate in this study was provided by the participants' legal guardian/next of kin.

## Author Contributions

AL, MH, RA, CA, FZ, ML, KM, DL, GA, DS, and SV secured funding. AL, MH, RA, AT, CA, FZ, ML, KM, DL, GA, DS, SV, ML'A, BK, and RP contributed to research design. AL, WQ, AT, GSA, and ML were responsible for data collection. AL, SV, RA, and GSA performed statistical analysis. AL drafted the manuscript. All authors provided critical inputs for the initial draft, read, and approved the final manuscript.

## Conflict of Interest

The authors declare that the research was conducted in the absence of any commercial or financial relationships that could be construed as a potential conflict of interest.

## Publisher's Note

All claims expressed in this article are solely those of the authors and do not necessarily represent those of their affiliated organizations, or those of the publisher, the editors and the reviewers. Any product that may be evaluated in this article, or claim that may be made by its manufacturer, is not guaranteed or endorsed by the publisher.

## Supplementary Material

Appendix 1 TV monitoring advert coding sheet.

Appendix 2 Outdoor advertising assessment tool.

Appendix 3 Tool for mapping food provision and retail outlets.

Appendix 4 In-store marketing assessment tool.

Appendix 5 Exit interview or post-purchase survey.

Appendix 6 Tool for identifying food provisioning policies and programmes.

Appendix 7 Policy-rating tool.

Appendix 8 IDI guide for school heads and administrators.

Appendix 9 IDI guide for caterers.

Appendix 10 M & E review tool.

Appendix 11 Onsite observations and compliance assessment tool.

Appendix 12 Qualitative focus group discussion guide.

Appendix 13 Qualitative KII guide for parents.

Appendix 14 CRM Key Informant tool.

Appendix 15 Net-Mapping tool.

Appendix 16 Study tool development and adaptation to Ghana context.

## Abbreviations

CRM: Community readiness model
EMIS: Education management information system
Food EPI: Healthy Food Environment Policy Index
GES: Ghana Education Service
GHS: Ghana Health Service
GSFP: Ghana School Feeding Programme
HIC: High-income country
IDIs: Qualitative in-depth interviews
IDRC: The International Development Research Centre
INFORMAS: International Network for Food and Obesity/non-communicable diseases Research Monitoring and Action Support
IRB: Institutional Review Board
JHS: Junior high school
LMICs: Low- and middle-income countries
MEALS4NCDs: Measurement, Evaluation, Accountability, and Leadership Support for NCDs prevention
MOH: Ministry of Health (Ghana)
NCDs: Non-communicable diseases
NR-NCDs: Nutrition-related NCDs
PS: Primary schools
PSBS: Public sector basic schools
PSUs: Primary sampling units
RCO: Regional Coordinating Offices of the Ghana School Feeding Programme
SHS: Senior high school
WHA: World Health Assembly
WHO: World Health Organization
WP: Work package.

## References

[1] World Health Organization. Noncommunicable Diseases Progress Monitor. Geneva: World Health Organization; Licence: CC BY-NC-SA 3.0 IGO (2017).

[2] Ministry of Health. National Policy for the prevention and Control of NCDs in Ghana 2012. Accra: Ministry of Health (2012).

[3] Abarca-Gómez L Abdeen ZA Hamid ZA Abu-Rmeileh NM Acosta-Cazares B Acuin C. Worldwide trends in body-mass index, underweight, overweight, and obesity from 1975 to 2016: a pooled analysis of 2416 population-based measurement studies in 128· 9 million children, adolescents, and adults. Lancet. (2017) 390:2627–42. 10.1016/S0140-6736(17)32129-3 29029897 PMC5735219

[4] Agyemang C Boatemaa S Agyemang Frempong G de-Graft Aikins A. Obesity in sub-Saharan Africa. In: Ahima RS, editor. Metabolic Syndrome: A Comprehensive Textbook. Cham: Springer (2016). p. 41–53. 10.1007/978-3-319-11251-0_5

[5] Amugsi DA Dimbuene ZT Mberu B Muthuri S Ezeh AC. Prevalence and time trends in overweight and obesity among urban women: an analysis of demographic and health surveys data from 24 African countries, 1991–2014. BMJ Open. (2017) 7:e017344. 10.1136/bmjopen-2017-017344 29079606 PMC5665233

[6] Steyn NP McHiza ZJ. Obesity and the nutrition transition in Sub-Saharan Africa. Ann N Y Acad Sci. (2014) 1311:88–101. 10.1111/nyas.12433 24725148

[7] Ofori-Asenso R Agyeman AA Laar A Boateng D. Overweight and obesity epidemic in Ghana—a systematic review and meta-analysis. BMC Public Health. (2016) 16:1239. 10.1186/s12889-016-3901-4 27938360 PMC5148846

[8] N. C. D. Risk Factor Collaboration - Africa Working Group. Trends in obesity and diabetes across Africa from 1980 to 2014: an analysis of pooled population-based studies. Int J Epidemiol. (2017) 46:1421–32. 10.1093/ije/dyx078 28582528 PMC5837192

[9] Ofori-Asenso R Agyeman AA Laar A. Metabolic syndrome in apparently “Healthy” Ghanaian adults: a systematic review and meta-analysis. Int J Chronic Dis. (2017) 2017:2562374. 10.1155/2017/2562374 29130065 PMC5654269

[10] Price AJ Crampin AC Amberbir A Kayuni-Chihana N Musicha C Tafatatha T. Prevalence of obesity, hypertension, and diabetes, and cascade of care in sub-Saharan Africa: a cross-sectional, population-based study in rural and urban Malawi. Lancet Diabetes Endocrinol. (2018) 6:208–22. 10.1016/S2213-8587(17)30432-1 29371076 PMC5835666

[11] World Health Organization. Global status report on noncommunicable diseases 2014: attaining the nine global noncommunicable diseases targets; a shared responsability. Geneva: World Health Organization (2014).

[12] Amuna P Zotor FB. Epidemiological and nutrition transition in developing countries: impact on human health and development: the epidemiological and nutrition transition in developing countries: evolving trends and their impact in public health and human development. Proc Nutr Soc. (2008) 67:82–90. 10.1017/S0029665108006058 18234135

[13] Abrahams Z McHiza Z Steyn NP. Diet and mortality rates in Sub-Saharan Africa: stages in the nutrition transition. BMC Public Health. (2011) 11:801. 10.1186/1471-2458-11-801 21995618 PMC3209469

[14] Ghana Statistical Service (GSS) GHSG and ICF International. Ghana Demographic and Health Survey. (2014). Rockville, MD: GSS. GHS, and ICF International (2015).

[15] Mensah AO. Contract management, monitoring and evaluation of Ghana school feeding programme at Atwima Kwanwoma District. Int J Sci Technol Res. (2016) 5:268–78.

[16] Ghana Statistical Service - GSS and Macro International. Ghana Demographic and Health Survey 1993. Calverton, MD: GSS and Macro International (1994).

[17] Aryeetey R Lartey A Marquis GS Nti H Colecraft E Brown P. Prevalence and predictors of overweight and obesity among school-aged children in urban Ghana. BMC Obes. (2017) 4:38. 10.1186/s40608-017-0174-0 29214030 PMC5715494

[18] Agyemang C Owusu-Dabo E de Jonge A Martins D Ogedegbe G Stronks K. Overweight and obesity among Ghanaian residents in The Netherlands: how do they weigh against their urban and rural counterparts in Ghana? Public Health Nutr. (2009) 12:909–16. 10.1017/S1368980008003510 18761759

[19] Dixon H Scully M Kelly B Donovan R Chapman K Wakefield M. Counter-advertising may reduce parent's susceptibility to front-of-package promotions on unhealthy foods. J Nutr Educ Behav. (2014) 46:467–74. 10.1016/j.jneb.2014.05.008 25034347

[20] Green MA Pradeilles R Laar A Osei-Kwasi H Bricas N Coleman N. Investigating foods and beverages sold and advertised in deprived urban neighbourhoods in Ghana and Kenya: a cross-sectional study. BMJ open. (2020) 10:e035680. 32595155 10.1136/bmjopen-2019-035680 PMC7322322

[21] Amevinya GS Quarpong W Laar A. Commercial food advertising on the campus of Ghana's largest University. World Nutr. (2020) 11:57–73. 10.26596/wn.202011257-73

[22] Konlan MB Laar AK. Nutrition standards and nature of foods sold at University of Ghana Canteens. In: World Public Health Nutrition Congress 2020 31 March to 2 April 2020. Brisbane: Brisbane Convention and Exhibition Centre (2019).

[23] Laar A Barnes A Aryeetey R Tandoh A Bash K Mensah K. Implementation of healthy food environment policies to prevent nutrition-related non-communicable diseases in Ghana: National experts' assessment of government action. Food Policy. (2020) 93:101907. 32565610 10.1016/j.foodpol.2020.101907 PMC7299075

[24] Asiki G Wanjohi MN Barnes A Bash K Muthuri S Amugsi D. Benchmarking food environment policies for the prevention of diet-related non-communicable diseases in Kenya: National expert panel's assessment and priority recommendations. Plos one. (2020) 15:e0236699. 32760079 10.1371/journal.pone.0236699 PMC7410300

[25] Glanz K Sallis JF Saelens BE Frank LD. Healthy nutrition environments: concepts and measures. Am J Health Promot. (2005) 19:330–3. 10.4278/0890-1171-19.5.330 15895534

[26] Story M Kaphingst KM Robinson-O'Brien R Glanz K. Creating healthy food and eating environments: policy and environmental approaches. Annu Rev Public Health. (2008) 29:253–72. 10.1146/annurev.publhealth.29.020907.090926 18031223

[27] Swinburn B Sacks G Vandevijvere S Kumanyika S Lobstein T Neal B. INFORMAS (International Network for Food and Obesity/Non-Communicable Diseases Research, Monitoring and Action Support): overview and key principles. Obesity Rev. (2013) 14:1–12. 10.1111/obr.12087 24074206

[28] Ferraro KF Thorpe RJ Jr Wilkinson JA. The life course of severe obesity: does childhood overweight matter? J Gerontol B Psychol Sci Soc Sci. (2003) 58:S110–9. 10.1093/geronb/58.2.S110 12646600 PMC3358723

[29] Ministry of Health. Policy for the Prevention and Control of Non-Communicable Diseases in Ghana 2020. Accra: Ministry of Health (2020).

[30] Laar AK Adler AJ Kotoh AM Legido-Quigley H Lange IL Perel P. Health system challenges to hypertension and related non-communicable diseases prevention and treatment: perspectives from Ghanaian stakeholders. BMC Health Serv Res. (2019) 19:693. 10.1186/s12913-019-4571-6 31615529 PMC6792211

[31] Laar A Barnes A Aryeetey R Tandoh A Bash K Mensah K. Implementation of healthy food environment policies to prevent nutrition-related non-communicable diseases in Ghana: National experts' assessment of government action. Food Policy 2020:101907. 10.1016/j.foodpol.2020.101907 32565610 PMC7299075

[32] World Health Organization. Marketing of food and non-alcoholic beverages to children. Sixty-Third World Health Assembly (No. Resolution WHA63. 14). Geneva: World Health Organization (2010).

[33] World Health Organization. A framework for implementing the set of recommendations on the marketing of foods and non-alcoholic beverages to children. Geneva: World Health Organization (2012).

[34] Government of Ghana. National School Feeding Policy. Accra: Ministry of Gender, Children and Social Protection (2015).

[35] Fernandes M Folson G Aurino E Gelli A. A free lunch or a walk back home? The school food environment and dietary behaviours among children and adolescents in Ghana. Food Secur. (2017) 9:1073–90. 10.1007/s12571-017-0712-0 32983282 PMC7473073

[36] Park MH Falconer C Viner RM Kinra S. The impact of childhood obesity on morbidity and mortality in adulthood: a systematic review. Obes Rev. (2012) 13:985–1000. 10.1111/j.1467-789X.2012.01015.x 22731928

[37] Reilly JJ Kelly J. Long-term impact of overweight and obesity in childhood and adolescence on morbidity and premature mortality in adulthood: systematic review. Int J Obes. (2011) 35:891–8. 10.1038/ijo.2010.222 20975725

[38] Kroll F Swart EC Annan RA Thow AM Neves D Apprey C. Mapping obesogenic food environments in South Africa and Ghana: correlations and contradictions. Sustainability. (2019) 11:3924. 10.3390/su11143924

[39] Hruby A Manson JE Qi L Malik VS Rimm EB Sun Q. Determinants and consequences of obesity. Am J Public Health. (2016) 106:1656–62. 10.2105/AJPH.2016.303326 27459460 PMC4981805

[40] Malik VS Willett WC Hu FB. Global obesity: trends, risk factors and policy implications. Nat Rev Endocrinol. (2013) 9:13. 10.1038/nrendo.2012.199 23165161

[41] Mozaffarian D Hao T Rimm EB Willett WC Hu FB. Changes in diet and lifestyle and long-term weight gain in women and men. New Eng J Med. (2011) 364:2392–404. 10.1056/NEJMoa1014296 21696306 PMC3151731

[42] Reeve B. Self regulation of food advertising to children: an effective tool for improving the food marketing environment. Monash UL Rev. (2016) 42:419.

[43] Cairns G Angus K Hastings G Caraher M. Systematic reviews of the evidence on the nature, extent and effects of food marketing to children. A retrospective summary. Appetite. (2013) 62:209–15. 10.1016/j.appet.2012.04.017 22561190

[44] Kelly B King L Baur L Rayner M Lobstein T Monteiro C. Monitoring food and non-alcoholic beverage promotions to children. Obes Rev. (2013) 14:59–69. 10.1111/obr.12076 24074211

[45] Cairns G Angus K Hastings G Organization WH. The Extent, Nature and Effects of Food Promotion to Children: A Review of the Evidence to December 2008. Geneva: World Health Organization (2009).

[46] Hastings G Stead M McDermott L Forsyth A MacKintosh AM Rayner M. Review of Research on the Effects of Food Promotion to Children. London: Food Standards Agency (2003).

[47] Hawkes C. Marketing Food to Children: The Global Regulatory Environment. Geneva: World Health Organization (2004).

[48] Hawkes C. Regulating and litigating in the public interest: regulating food marketing to young people worldwide: trends and policy drivers. Am J Public Health. (2007) 97:1962–73. 10.2105/AJPH.2006.101162 17901436 PMC2040356

[49] Hastings G McDermott L Angus K Stead M Thomson S. The Extent, Nature and Effects of Food Promotion to Children: A Review of the Evidence. Geneva: World Health Organization (2006). p. 20.

[50] Kessler DA. The End of Overeating: Taking Control of the Insatiable American Appetite. Emmaus , PA: Rodale (2010).

[51] Gibson EL. Emotional influences on food choice: sensory, physiological and psychological pathways. Physiol Behav. (2006) 89:53–61. 10.1016/j.physbeh.2006.01.024 16545403

[52] Swinburn B Vandevijvere S Kraak V Sacks G Snowdon W Hawkes C. Monitoring and benchmarking government policies and actions to improve the healthiness of food environments: a proposed Government Healthy Food Environment Policy Index. Obes Rev. (2013) 14:24–37. 10.1111/obr.12073 24074208

[53] UN Resolution A/RES/66/2. Political declaration of the High-level Meeting of the General Assembly on the prevention and control of non-communicable diseases. In: Sixty-sixth United Nations General Assembly. New York, NY: United Nations (2011). Available online at: http://undocs.org/en/A/66/L.1 (accessed September 18, 2020).

[54] UN Resolution A/RES/73/2. Political declaration of the third high-level meeting of the General Assembly on the prevention and control of noncommunicable diseases. in Seventy-third United Nations General Assembly. New York, NY: United Nations (2018). Available online at: http://www.un.org/en/ga/search/view_doc.asp?symbol=A/RES/73/2 (accessed September 15, 2020).

[55] UNICEF. The State of the World's Children 2019. Children. Food and Nutrition: Growing well in a changing world. New York, NY: UNICEF (2019).

[56] L'Abbe M Schermel A Minaker L Kelly B Lee A Vandevijvere S. Monitoring foods and beverages provided and sold in public sector settings. Obes Rev. (2013) 14:96–107. 10.1111/obr.12079 24074214

[57] Aberman N-L Birner R Haglund E Ngigi M Ali S Okoba B. Understanding the Policy Landscape for Climate Change Adaptation: A Cross-Country Comparison Using the Net-Map Method. Washington, DC: IFPRI (2015). 10.2139/ssrn.2564536

[58] Oetting E Plested B Edwards R Thurman P Kelly K Beauvais F. Community readiness for community change. In: Stanley L, editor. Tri-Ethnic Center Community Readiness Handbook. 2nd ed. Fort Collins, CO: Colorado State University (2014).

[59] Pradeilles R Marr C Laar A Holdsworth M Zotor F Tandoh A. How ready are communities to implement actions to improve diets of adolescent girls and women in urban Ghana?BMC Public Health. (2019) 19:646. 10.1186/s12889-019-6989-5 31138180 PMC6537223

[60] GeoPoll. Ghana Media Measurement Report: Top TV, Radio. And Print Outlets. (2017). Available online at: https://www.geopoll.com/blog/ghana-media-measurement-report-top-tv-radio-print-outlets-2017/#:%7Bsim%20%7D:textprotect%20elax%20=%7B%7DTOP%20TV%20STATIONS%20IN%20GHANA,lead%20in%20viewers%20each%20quarter (accessed September 14, 2020).

[61] Watson A. Number of pay TV households in Ghana from 2014 to 2023. Available online at: https://www.statista.com/statistics/756362/ghana-number-pay-tv-households/ (accessed September 14, 2020).

[62] Riffe D Lacy S Fico FG. Analyzing Media Messages: Using Quantitative Content Analysis in Research. London: Lawrence Erlbaum Associates (1998).

[63] Laar AK. Family planning, abortion, and HIV in Ghanaian print media: a 15-month content analysis of a national Ghanaian newspaper. Afr J Reprod Health. (2010) 14:80–6. 21812201

[64] Kelly B. INFORMAS Protocol: Food Promotion Module: Food Marketing - Television Protocol. Auckland: The University of Auckland.

[65] Ghana Education Service. Enrolment data. Ghana Education Service- Education Management Information System (2018).

[66] Green M Pradeilles R Osei-Kwasi H Bricas N Aryeetey R Griffiths P. Characterising the food environments of deprived neighbourhoods in three African cities. In: Proceedings of the 8th Africa Nutrition Conference. Addis Ababa (2018).

[67] Monteiro CA Cannon G Moubarac J-C Levy RB Louzada MLC Jaime PC. The UN Decade of Nutrition, the NOVA food classification and the trouble with ultra-processing. Public Health Nutr. (2018) 21:5–17. 10.1017/S1368980017000234 28322183 PMC10261019

[68] Green S H Glanz K. Development of the perceived nutrition environment measures survey. Am J Prev Med. (2015) 49:50–61. 10.1016/j.amepre.2015.02.004 26094227

[69] Holdsworth M Pradeilles R Tandoh A Green M Wanjohi M Zotor F. Unhealthy eating practices of city-dwelling Africans in deprived neighbourhoods: evidence for policy action from Ghana and Kenya. Glob Food Secur. (2020) 26:100452. 10.1016/j.gfs.2020.100452 33324537 PMC7726234

[70] Edwards RW Jumper-Thurman P Plested BA Oetting ER Swanson L. Community readiness: research to practice. J Commun Psychol. (2000) 28:291–307. 10.1002/(SICI)1520-6629(200005)28:3<291::AID-JCOP5>3.0.CO;2-9

[71] Borresen EC Stone C Boré A Cissoko A Maiga A Koita OA. Assessing Community Readiness to Reduce Childhood Diarrheal Disease and Improve Food Security in Dioro, Mali. Int J Environ Res Public Health. (2016) 13:571. 10.3390/ijerph13060571 27338428 PMC4924028

[72] Pradeilles R Rousham EK Norris SA Kesten JM Griffiths PL. Community readiness for adolescents' overweight and obesity prevention is low in urban South Africa: a case study. BMC Public Health. (2016) 16:763. 10.1186/s12889-016-3451-9 27515802 PMC4982405

[73] Schiffer E Waale D. Tracing power and influence in networks: Net-Map as a tool for research and strategic network planning. IFPRI discussion papers 772. International Food Policy Research Institute (IFPRI) (2008).

[74] Plested BA Edwards RW Jumper-Thurman P. Community Readiness: A Handbook for Successful Change: Tri-Ethnic Center for Prevention Research. Fort Collins, CO: Colorado State University (2006).

[75] Tong A Sainsbury P Craig J. Consolidated criteria for reporting qualitative research (COREQ): a 32-item checklist for interviews and focus groups. Int J Qual Health Care. (2007) 19:349–57. 10.1093/intqhc/mzm042 17872937

[76] Hu Y. Efficient, high-quality force-directed graph drawing. Mathemat J. (2005) 10:37–71.

[77] Von Elm E Altman DG Egger M Pocock SJ Gøtzsche PC Vandenbroucke JP. The strengthening the reporting of observational studies in epidemiology (STROBE) statement: guidelines for reporting observational studies. Ann Int Med. (2007) 147:573–7. 10.7326/0003-4819-147-8-200710160-00010 17938396

[78] Wertheim-Heck SC Raneri JE. A cross-disciplinary mixed-method approach to understand how food retail environment transformations influence food choice and intake among the urban poor: experiences from Vietnam. Appetite. (2019) 142:104370. 10.1016/j.appet.2019.104370 31310835 PMC6739597

