# Supplementary material for: Providing Measurement, Evaluation, Accountability, and Leadership Support (MEALS) for Non-communicable Diseases Prevention in Ghana: Project Implementation Protocol
Source: Front Nutr. 2021 Aug 18;8:644320. doi: 10.3389/fnut.2021.644320 (PMC8416277; doi:10.3389/fnut.2021.644320)
Supplement: Appendix 1 — TV monitoring advert coding sheet. [file Table_1.DOCX]

**TELEVISION MONITORING**

**PROJECT TITLE: Measuring the Healthiness of Ghanaian Children's Food Environments to Prevent Obesity and Non-Communicable Diseases**

Name of interviewer:

Date of interview |__||__|/|__||__|/|__||__||__||__|

| **Spreadsheet variable** | **Description of data field** |
| --- | --- |
| **Variable 1:** | Country name |
| **Variable 2:** | Data collection area (country) |
| **Variable 3:** | Data collection year (yyyy) |
| **Variable 4:** | Channel name (e.g. GTV) |
| **Variable 5:** | Channel audience share in peak viewing times (percentage) |
| **Variable 6:** | Date of recording (dd-mm-yyyy) |
| **Variable 7:** | Day of the week (Monday/Tuesday/Wednesday/Thursday/Friday/Saturday/Sunday) |
| **Variable 8:** | Program name in which the advertisement is shown (Text format) |
| **Variable 9:** | Program category |
|  | **1** = News, commentary, political programs  **2** = Sport (a specific program or a sport event)  **3** = Soap opera  **4** = Series (not specifically for children)  **5** = Movie (not specifically for children)  **6** = Documentary  **7** = Reality show  **8** = Talk show  **9 =** Miscellaneous entertainment: e.g. variety, contests  **10** = Children: cartoon, movies, series or other show for children  **11** = Music or music video  **12** = Religious  **13** = Health related  **14** = Other |
| **Variable 10:** | Starting time of programme |
| **Variable 11:** | Ending time of programme |
| **Variable 12:** | The time slot of Advertisement (Codes: 1-16 using Table 1) |
| **Variable 13:** | Start time of advertisement |
| **Variable 14:** | End time of advertisement |
| **Variable 15a:** | Moment of advertisement |
|  | 1 = As a pause of a specific programme  2 = Between two programmes  3 = During programme |
| **Variable 15b:** | If during a programme |
|  | 3a = News/Ad bar |
| **Variable 16:** | Advertisement type |
|  | 1 = food or drink product- food company/brand  2 = food or drink product- promoted in advertisement by non-food brand/company/retailer /service/event  3 = food or drink company or brand (no retailer) without food or drink product  4 = food or drink retailer (supermarket or convenience store) with food or drink product  5 = food or drink retailer (supermarket or convenience store) without food or drink product  6 = food or drink retailer (restaurant or takeaway or fast food) with food or drink product  7 = food or drink retailer (restaurant or takeaway or fast food) without food or drink product  8 = non-food or drink product  IF ADVERTISEMENT IS CODED AS 8 – A NON-FOOD PRODUCT THEN GO TO NEXT ADVERTISEMENT  IF ADVERTISEMENT CODED 1-7, THEN THE FOLLOWING CODES ARE TO BE COMPLETED TO DESCRIBE THE FOOD RELATED ADVERTISEMENT IN MORE DETAIL |
| **Variable 17:** | Company name; (e.g. Nestle, Unilever, ) |
| **Variable 18:** | Name of product advertised |
| **Variable 19a:** | Description of product advertised  Note: Describe the product advertised in a way to aid collecting of nutrition information. (e.g. “KFC meal containing a burger, medium fries and medium soft drink” rather than just “KFC meal”; “chocolate coated, cream-filled biscuit/cookie” rather than just “biscuit/cookie).  If no foods or drinks were advertised, describe what the food company advertisement was for (e.g. “competition to win a family holiday, purchase any marked packet and visit website: www... to enter competition” or “Company character KFC playing with children in an open playground”). |
| **Variable 19b:** | Food product category (See Table 2: Core/Non-core) (1-37) |
| **Variable 20:** | Permission to market product |
|  | 0= Not permitted to be marketed to children by WHO  1= Permitted to be marketed to children by WHO  2 = Advert approved to be marketed by FDA  3= Advert not approved to be marketed by FDA  4= Non-Specified (in case not sufficient information available to classify)  5= Not applicable |
| **Variable 21:** | Power of advertising |
|  | 0 = No strategies used  1= Strategy used |
| **Variable 22:** | Advertising strategy used |
|  | 1 = Cartoon/Company owned character e.g. M&Ms  2 = Licenced character e.g. Dora the explorer  3 = Amateur sportsperson e.g. person playing a sport  4 = Celebrity (non-sports) e.g. Funny Face  5 = Movie tie-in e.g. Shrek  6 = Famous sportsperson/team e.g. Asamoah Gyan/Ghana Black Star  7 = Non-sports/historical events/festivals e.g. Christmas Day  8 = ‘For kids’ e.g. image of a child, ‘for school lunch boxes’  9 = Awards e.g. Best Food Award 2019, Number one best-selling’  10 = Sports event |
| **Variable 23:** | Power of advertising description |
|  | Example, if you have coded ‘4’ and ‘1’ for the presence of strategies to increase the power of advertising, please describe all strategies in the advertisement, e.g. naming the celebrity (non-sports) ‘Funny face’. |
| **Variable 24:** | Premium offers |
|  | 0 = No premium offered  1= Premium offered |
| **Variable 25:** | Premium offers type |
|  | 1= Game and app downloads  2= Contests  3= Pay 2 take 3 or other  4= 20% extra or other  5= Limited edition  6= Social charity  7= Gift or collectable  8= Price discount  9= Loyalty programs |

**Table 1: The timeslots, per 60-minute period**

| ***Time***  ***(24-hour format)*** | ***Code Timeslot*** |
| --- | --- |
| 6:00-6:59 | 1 |
| 7:00-7:59: | 2 |
| 8:00-8:59 | 3 |
| 9:00-9:59 | 4 |
| 10:00-10:59 | 5 |
| 11:00-11:59 | 6 |
| 12:00-12:59 | 7 |
| 13:00-13:59 | 8 |
| 14:00-14:59 | 9 |
| 15:00-15:59 | 10 |
| 16:00-16:59 | 11 |
| 17:00-17:59 | 12 |
| 18:00-18:59 | 13 |
| 19:00-19:59 | 14 |
| 20:00-20:59 | 15 |
| 21:00-21:59 | 16 |
| 22:00-22:59 | 17 |
| 23:00-24:00 | 18 |
