# Supplementary material for: Providing Measurement, Evaluation, Accountability, and Leadership Support (MEALS) for Non-communicable Diseases Prevention in Ghana: Project Implementation Protocol
Source: Front Nutr. 2021 Aug 18;8:644320. doi: 10.3389/fnut.2021.644320 (PMC8416277; doi:10.3389/fnut.2021.644320)
Supplement: Appendix 2 — Outdoor advertising assessment tool. [file Table_2.DOCX]

**OUTDOOR ADVERTISING ASSESSMENT**

**PROJECT TITLE: Measuring the Healthiness of Ghanaian Children's Food Environments to Prevent Obesity and Non-Communicable Diseases**

Name of Data collector:

Date of Data collection: |__||__|/|__||__|/|__||__||__||__|

|  | **Variable** | **Response** |
| --- | --- | --- |
| **Description of study site** | | |
| **Variable 1:** | District | 1. Accra Metropolitan 2. Ningo Prampram District 3. Kpone Katamanso District 4. La Nkwantanang Madina 5. Ga South Municipal 6. Ashaiman Municipal |
| **Variable 2:** | Name of school | **……………………** |
| **Variable 3:** | Type of school | 1. Primary only 2. Junior High school only 3. Both Primary and JHS |
| **Advertising Data** | | |
| **Variable 4:** | Advertisement ID | ……………………………… |
| **Variable 5:** | Advertisement category | 1. Food Product/brand 2. Non food product/band |
| **Variable 6:** | Photo of food advertisement |  |
| **Variable 7:** | Size of advertisement | 1. Small (>A4 but <1.3m x 1.9m) 2. Medium (>1.3m x 1.9m but <2.0m x 2.5m) 3. Large (> 2m x 2.5m) |
| **Variable 8:** | Setting of advertisement | 1. Food shop 2. Road 3. Building 4. Bus shelter 5. Mobile cart/stall or vending machine 6. Other Specify |
| **Variable 09:** | Type of advertisement | 1. Billboard 2. Poster 3. Banner 4. Free-standing sign 5. Painted building / wall 6. Digital Signs / LED 7. Merchandising 8. Other Specify |
| **Variable 10:** | Number of food product type in the advertisement | 1. Only company/brand mentioned 2. Single food product type 3. Two food product types 4. Three food product types 5. Other Specify |
| **Variable 11:** | Number of same food product advertisement at the same location? | ……………………………. |
| **FOR EACH FOOD PRODUCT TYPE IN THE ADVERTISEMENT** | | |
| **Variable 12:** | Brand name1 | ……………………………. |
| **Variable 13:** | Product name1 | ……………………………. |
| **Variable 14:** | Product/Advertisement description1 |  |
| **Variable 15:** | Promotional characters1 | 1. No character present 2. Cartoon/Company owned charactere.g. M&Ms 3. Licensed character e.g. Dora the explorer 4. Amateur sportsperson e.g. Person playing a sport 5. Celebrity (non-sports) e.g. Shatta Wale 6. Celebrity (Famous sportsperson/team) e.g. Asamoah Gyan 7. Non-sports/historical events/festivals e.g. Christmas 8. For kids e.g. image of a youth, ‘great for school lunches’, 9. Awards e.g. Best Food Award 2014, ‘award winning’, ‘number one best-selling’ 10. Sports event 11. Other specify…………………………………………… |
| **Variable 16** | Premium offers1 | 1. No Premium offer 2. Game and app downloads 3. Contests 4. Pay 2 take 3 or other 5. 20% extra or other 6. Limited edition 7. Social charity 8. Gift or collectable 9. Price discount 10. Loyalty programs 11. Other specify…………………………………………… |
| **Variable 12a:** | Brand name2 |  |
| **Variable 13a:** | Product name2 |  |
| **Variable 15a:** | Promotional characters2 |  |
| **Variable 19a:** | Premium offers2 |  |
| **Variable 17:** | GPS Coordinate |  |
