# Supplementary material for: Providing Measurement, Evaluation, Accountability, and Leadership Support (MEALS) for Non-communicable Diseases Prevention in Ghana: Project Implementation Protocol
Source: Front Nutr. 2021 Aug 18;8:644320. doi: 10.3389/fnut.2021.644320 (PMC8416277; doi:10.3389/fnut.2021.644320)
Supplement: Appendix 3 — Tool for mapping food provision and retail outlets. [file Table_3.DOCX]

**FOOD PROVISION AND RETAIL OUTLETS**

**PROJECT TITLE: Measuring the Healthiness of Ghanaian Children's Food Environments to Prevent Obesity and Non-Communicable Diseases**

Name of Data collector:

Date of Data collection: |__||__|/|__||__|/|__||__||__||__|

|  | **Variables** | **Response** |
| --- | --- | --- |
| Q1 | Food outlet ID |  |
| Q2 | District | 1. Accra Metropolitan 2. Ningo Prampram District 3. Kpone Katamanso District 4. La Nkwantanang Madina 5. Ga South Municipal 6. Ashaiman Municipal |
| Q3 | Name of school | ……………… |
| Q4 | Type of school | 1. Primary school only 2. Junior High School only 3. Both Primary and JHS school |
| Q5 | Photo of food outlet | ………… |
| Q6 | Type of food outlet | 1. Supermarket 2. Convenient/Provision shop 3. School Canteen 4. Kiosk 5. Food stall/stand 6. Table top 7. Restaurant 8. Chop bar 9. Cold store 10. Open market 11. Bakery 12. Fruit/vegetable stand 13. Other |
| Q7 | Food outlet location | 1. Inside of school premises 2. Outside of school premises |
| Q8 | Food outlet status | 1. Formal retailer –Licensed to provide/sell food outlet 2. Informal retailer – self organized unlicensed food provision business |
| Q9 | Type of Business | 1. Individual 2. Partnership 3. Chain / Franchising |
| Q10 | Food outlet operator’s gender | 1. Male 2. Female |
| Q11 | Duration of operation of food outlet | ……… years |
| Q12 | Type of services | 1. Self service 2. Assisted service 3. Both self and assisted service |
| Q13 | Food on sale  [Multiple choice] | 1. Sugary foods (eg. Ice-cream, cake, candy) except SSB 2. Sugar, Sugar Sweetened Beverage (eg. Fanta, Sprite, Coca cola) 3. Fresh fruits and unsweetened canned fruits (eg. Mango, Orange, Blue Skies) 4. Canned fruits with added sugar (eg. Don Simon Fruit juice) 5. Fresh vegetables and unsalted canned vegetables (eg. Cucumber, carrot, onion) 6. Canned vegetables with added salt 7. Snacks (eg. Savoury crackers, Crisps, sweetened popcorn, salted popcorn, cassava crips, plantain crips, chips) 8. Salted foods (eg. salted nuts and salty snacks) 9. Whole grain bread or cereal with no added sugar 10. Refined grains and refined grains products (eg. White bread, white rice) 11. Fresh Fish, Meat, egg 12. Processed Fish, Meat, Chicken products 13. Cooked/Mixed Dishes (eg. Waakye, jollof rice, red red, Apapransa, kpokpoi, fried rice,anku) 14. other |
| Q14 | Days of operation  [Multiple choice] | 1. Monday 2. Tuesday 3. Wednesday 4. Thursday 5. Friday 6. Saturday 7. Sunday |
| Q15 | Hours of operation | Opening time:  Closing time: |
| Q16 | Sitting/Convenience area for immediate consumption of bought item? | 1. Yes 2. No |
| Q17 | GPS coordinate of Food outlet | …….…… |
