# Supplementary material for: Providing Measurement, Evaluation, Accountability, and Leadership Support (MEALS) for Non-communicable Diseases Prevention in Ghana: Project Implementation Protocol
Source: Front Nutr. 2021 Aug 18;8:644320. doi: 10.3389/fnut.2021.644320 (PMC8416277; doi:10.3389/fnut.2021.644320)
Supplement: Appendix 4 — In-store marketing assessment tool. [file Table_4.DOCX]

IN-STORE MARKETING ASSESSMENT

PROJECT TITLE: Measuring the Healthiness of Ghanaian Children's Food Environments to Prevent Obesity and Non-Communicable Diseases

**Instructions**

Remember to enter the supermarket from the side marked as "Entrance" on either the left or right side and go around the perimeter of the store. Fill in the instrument below about the perimeter of the store starting from the "Exit/entrance" area of the store and pay attention to the details of audiovisual advertisements that you hear and observe.

| **Variable** | **Choices** |
| --- | --- |
| **1.General information:** | |
| Name of data collector | ....................... |
| Date of data collection | --/ --/ -----/ |
| **2. Information about the store** | |
| Supermarket Name | ..................... |
| Location of supermarket (Community Name) | ...................... |
| District |  |

| **3.Description of specific areas**  **3.1 Exit/ entrance area**  **3.1.1** For the following variety of products found at the exit /entrance area, record the number of shelves occupied by these food categories in relation to the shelves level. The Level reference are: Level 1: 0-100 cm, Level 2: 100-150 cm, Level 3: 150-190 cm, Level 4: 190 cm or more. Recognize the levels in relation to your height and observe in the direction of the floor to the ceiling. Take shelf 1 as the closest to the entrance. | | | | |
| --- | --- | --- | --- | --- |
| Food categories | Level/ Number of shelves | | | |
|  | Level 1 | Level 2 | Level 3 | Level 4 |
| Whole grain cereals (eg. Whole grain bread, whole grain breakfast cereal, local brown rice) |  |  |  |  |
| Refined grains and refined grain products (eg. White bread, white rice, oat, noodles, cornflakes,) |  |  |  |  |
| Legumes, oilseeds and nuts (eg. Peanut, tigernut, cashew nut, Soybean) |  |  |  |  |
| Fresh meat, fish, chicken |  |  |  |  |
| Processed Meat,Fish and Poultry (eg. Sausage) |  |  |  |  |
| Milk product (e.g. Cheese) |  |  |  |  |
| Fresh milk, yoghurt, sugar free milk |  |  |  |  |
| Eggs |  |  |  |  |
| Fresh fruits (eg. Orange, tangerine, watermelon, mango, pawpaw, pineapple, banana) |  |  |  |  |
| Fresh fruit juice/unsweetened canned Fruits (eg. Coconut juice, blue skies) |  |  |  |  |
| Canned fruits juice with added sugar (eg. Don Simon Fruit juice) |  |  |  |  |
| Fresh Vegetables and unsalted canned vegetables (eg. Cucumber, carrot, onion, spinach, lettuce, cabbage) |  |  |  |  |
| Canned vegetables with added salt |  |  |  |  |
| Fats/oil product (eg. margarine, butter) |  |  |  |  |
| Water |  |  |  |  |
| Alcoholic beverages (eg. Beer, wine, spirit) |  |  |  |  |
| Sweetened coffee/tea |  |  |  |  |
| Regular soft drinks (eg. Soda drink, Coca cola, Fanta) |  |  |  |  |
| Other sugar sweetened beverages (eg. brukina drink, sobolo) |  |  |  |  |
| Milk drink with sugar |  |  |  |  |
| Energy/Sports drink (eg. Lucozade energy drink, rush energy drink) |  |  |  |  |
| Cake, biscuit, cookies (eg. Sweet pie, doughnut, pancake) |  |  |  |  |
| Sweet foods (added sugars) eg. Toffee, chocolate, sugar, sweets, jam |  |  |  |  |
| Ready to eat cereals |  |  |  |  |
| Salted snack (eg. Fried plantain, fried sweet potato, chips, crisps salted popcorn) |  |  |  |  |
| Unprocessed staples (eg. Yam, Plantain) |  |  |  |  |
|  |  |  |  |  |

| **3.1.2 Types of strategies found in the exit /entrance area:** | | |  |
| --- | --- | --- | --- |
| Type of strategy | Yes | If yes what food group(s) does this strategy applies to? | No |
| Entertainers/animators |  |  |  |
| Discounts Magazine when entering the store |  |  |  |
| Discount coupons for activities (e.g., cinema) |  |  |  |
| Price reductions |  |  |  |
| Price comparisons with other stores |  |  |  |
| Discount (%, 3x2, among others) |  |  |  |
| Additional gifts (toys, utensils, among others) |  |  |  |
| Promotion on packaging (characters, cartoons, celebrities, athletes, events, among others...) |  |  |  |
| Promoters (with tasting) |  |  |  |
| Promoters (without tasting) |  |  |  |
| Special exhibitions on the shelf |  |  |  |
| Block display |  |  |  |
| Others, specify |  |  |  |

| **3.2 Check–outs Area** | |
| --- | --- |
| 3.2.1 Number of cash registers in-store | ------------- |
| 3.2.2 Is there a section of islands? | 1. Yes 2. No |
| 3.2.2.1 How many islands are there in the check-outs area? |  |
| 3.2.3 Is there a ‘dump’ section? | 1. Yes 2. No |
| 3.2.3.1 How many ‘dump sections’ are there in the check-outs area? |  |
| 3.2.4 Is there a section of rapid cash register (i.e. less than 10 items)? | 1. Yes 2. No |

| **3.3 Record the types of strategies found in the check-outs area** | | |  |
| --- | --- | --- | --- |
| Type of strategy | Yes | If yes what food groups does this strategy applies to | No |
| Entertainers/animators |  |  |  |
| Magazine of discounts when entering the store |  |  |  |
| Discount coupons for activities (e.g., cinema) |  |  |  |
| Price reductions |  |  |  |
| Price comparisons with other stores |  |  |  |
| Discount (%, 3x2, among others) |  |  |  |
| Additional gifts (toys, utensils, among others) |  |  |  |
| Promotion on packaging (characters, cartoons, celebrities, athletes, events, among others...) |  |  |  |
| Promoters (with tasting) |  |  |  |
| Promoters (without tasting) |  |  |  |
| Special exhibitions on the shelf |  |  |  |
| Block display |  |  |  |
| Others, specify |  |  |  |

| **3.4 Audiovisual strategies**  So far have you heard any kind of promotion inside or outside the store? Specify | |
| --- | --- |
| 3.4.1 Radio advertisements | Yes, Please specify  No |
| 3.4.2 Music | Yes, Please specify  No |
| 3.4.3 Videos on a screen | Yes, Please specify  No |
| 3.4.4 Other | Yes, Please specify  No |
| **4. Spatial distribution and product placement**  4.1 Mark with an X the departments that are in the supermarket | |
| a) Fresh produce |  |
| b) Ready meals |  |
| c) Babies |  |
| d) Snacks |  |
| e) Meat, fish and seafood |  |
| f) Beers, wines, liqueurs |  |
| g) Frozen products |  |
| h) Sweets |  |
| i) Pharmacy |  |
| j) Fruits and vegetables |  |
| l) Personal hygiene and beauty |  |
| m) Juices and beverages |  |
| n) Toys |  |
| o) Dairy products |  |
| p) Cleaning products |  |
| q) Bread and bakery products |  |
| r) Sausages and cheeses |  |
| s) Regular soft drinks/ drinks with gas |  |
| - 1. Others (describe the name of the departments): | A: _____________________  B: _____________________  C: _____________________ |

**5. Aisle breakdown**

NOTE: The following apply to each aisle of the store displaying food and non-alcoholic beverages.

| **5.1 Aisle**  Note: Aisle 1 is named "1" as stated by the store. If it is not numbered, take aisle 1 by taking the left side in the direction that the boxes are on the back. |
| --- |
| **Photo inventory of products in aisle 1**  5.1.1 Take photos of food and non-alcoholic beverage products found on shelves facing aisle 1 |
| 5.1.1 Take photos of all posters in Aisle 1: |

| **Final Comments** |
| --- |
| Time of finalization: |
| Add a photo(s) that shows what most caught your attention in the store you visit: |
| Before finishing, do you have any comments? |
