# Supplementary material for: Providing Measurement, Evaluation, Accountability, and Leadership Support (MEALS) for Non-communicable Diseases Prevention in Ghana: Project Implementation Protocol
Source: Front Nutr. 2021 Aug 18;8:644320. doi: 10.3389/fnut.2021.644320 (PMC8416277; doi:10.3389/fnut.2021.644320)
Supplement: Appendix 5 — Exit interview or post-purchase survey. [file Table_5.DOCX]

**EXIT INTERVIEW/POST-PURCHASE SURVEY**

**PROJECT TITLE: Measuring the Healthiness of Ghanaian Children's Food Environments to Prevent Obesity and Non-Communicable Diseases**

Instructions for the interviewer

Read the information sheet and informed consent letter to participant. If he/she agrees to participate, administer the questionnaire and do not exceed the set duration (10 minutes).

Inclusion Criteria:
1. A parent/guardian with child (ren) aged 6 -17 years
2. Parent/guardian should buy at least a food/beverage product from supermarket

Name of interviewer:

Date of interview |__||__|/|__||__|/|__||__||__||__|

| **Questions** | **Response** |
| --- | --- |
| 1. Name of Supermarket |  |
| 1. Location of Supermarket (Community name) |  |
| 1. District name | 1. Accra Metropolitan 2. Ningo Prampram District 3. Kpone Katamanso District 4. La Nkwantanang Madina 5. Ga South Municipal 6. Ashaiman Municipal |
| 1. Sex of respondent | 1. Male 2. Female |
| 1. Religion | 1. None 2. Christian 3. Muslim 4. Traditional 5. Other |
| 1. Age [in complete years] | .................... |
| 1. Level of education | 1. No formal education 2. Primary 3. JHS 4. SHS 5. Tertiary 6. Other |
| 1. Number of Children (<18years) in household? |  |
| 1. How often do YOU consume these categories of foods?   **Never**  **Rarely**  **Sometimes**  **Always** | 1. Sugary foods (eg. Ice-cream, cake, candy) except SSB 2. Sugar, Sugar Sweetened Beverage (eg. Fanta, Sprite, Coca cola) 3. Fresh fruits and unsweetened canned fruits (eg. Mango, Orange, Blue Skies) 4. Canned fruits with added sugar (eg. Don Simon Fruit juice) 5. Fresh vegetables and unsalted canned vegetables (eg. Cucumber, carrot, onion) 6. Canned vegetables with added salt 7. Snacks (eg. Savoury crackers, Crisps, sweetened popcorn, salted popcorn, cassava crips, plantain crips, chips) 8. Salted foods (eg. salted nuts and salty snacks) 9. Whole grain bread or cereal with no added sugar 10. Refined grains and refined grains products (eg. White bread, white rice) 11. Fresh Fish, Meat, egg 12. Processed Fish, Meat, Chicken products |
| 1. How often does your HOUSEHOLD consume these category of foods?   **Never**  **Rarely**  **Sometimes**  **Always** | 1. Sugary foods (eg. Ice-cream, cake, candy) except SSB 2. Sugar, Sugar Sweetened Beverage (eg. Fanta, Sprite, Coca cola) 3. Fresh fruits and unsweetened canned fruits (eg. Mango, Orange, Blue Skies) 4. Canned fruits with added sugar (eg. Don Simon Fruit juice) 5. Fresh vegetables and unsalted canned vegetables (eg. Cucumber, carrot, onion) 6. Canned vegetables with added salt 7. Snacks (eg. Savoury crackers, Crisps, sweetened popcorn, salted popcorn, cassava crips, plantain crips, chips) 8. Salted foods (eg. salted nuts and salty snacks) 9. Whole grain bread or cereal with no added sugar 10. Refined grains and refined grains products (eg. White bread, white rice) 11. Fresh Fish, Meat, egg 12. Processed Fish, Meat, Chicken products |
| **Participants Questions** |  |
| 1. What food outlet do you most frequently use for grocery shopping? | 1. Supermarket 2. Mini-Supermarket 3. Convenient/Provision shop 4. Kiosk 5. Food stall/stand 6. Table top 7. Restaurant 8. Fast food 9. Cold stores 10. Open market 11. Bakery 12. Other(s) |
| 1. Why do you mostly use [Ans to Q11] for grocery shopping? | 1. Proximity to school/home 2. On my usual route school/home 3. Types of products 4. Nutritional quality of products 5. Variety of productss 6. Price of products 7. Customer service 8. Other |
| 1. Why did you decide to shop at this supermarket today? [Multiple choice] | 1. Proximity to school/home 2. On my usual route school/home 3. Types of products 4. Nutritional quality of products 5. Variety of productss 6. Price of products 7. Customer service 8. Other |
| 1. How would you rate in general the healthiness (as in nutritional quality) of your shopping basket today? | 1. Very bad 2. Bad 3. Neutral 4. Good 5. Very Good |
| 14a. Can you explain the reason for your rating in [Q14] | ............ |
| 1. What foods and/or non-alcoholic beverage products particularly stood out to you in the shop?   Hint: Mention top three  Enter "nothing" in the space provided if no product stood out | Product 1 :  Why:  Product 2:  Why: |
| 1. Did you buy these items you mentioned | 1. Yes 2. No |
| 17. Why did you buy these products? | 1. I needed it 2. Price promotion was ongoing 3. Product display was ongoing 4. I wanted to try it 5. I always buy it 6. My child(ren) was asking for it 7. Other, specify…. |
| 1. During shopping, did you buy any products that you didn't plan buying? | 1. Yes 2. No 3. Not sure |
| 18a. If yes mention name(s) of products |  |
| 18b. Why did you buy these products | 1. I needed it 2. Price promotion was ongoing 3. Product display was ongoing 4. I wanted to try it 5. I always buy it 6. My peers were buying it 7. My child(ren) was asking for it 8. Other |
| 1. Did you see/hear any advertisements for foods or beverages in this supermarket when shopping? | 1. Yes 2. No 3. Not Sure |
| 1. Did you buy any foods/beverages because it was advertised either in this shop or another store? | 1. Yes 2. No   Not sure |
| 1. Before buying a product, do you read or look at the front/back of package where usually the nutritional information is displayed on the product? | 1. Yes 2. No |
| 21a What information do you look out for | …………………. |
| 1. Please indicate whether you agree or disagree with the following statements relating to this supermarket and your shopping habits at that store.   Options:  Strongly disagree  Somewhat disagree  Neutral  Somewhat agree  Strongly agree | 1. I notice signs that encourage me to purchase healthy foods 2. I often buy food items that are located near the register. 3. I often buy items that are at eye level on the shelves. 4. There are lots of signs and displays encouraging me to buy the unhealthy foods. |
