# Supplementary material for: Providing Measurement, Evaluation, Accountability, and Leadership Support (MEALS) for Non-communicable Diseases Prevention in Ghana: Project Implementation Protocol
Source: Front Nutr. 2021 Aug 18;8:644320. doi: 10.3389/fnut.2021.644320 (PMC8416277; doi:10.3389/fnut.2021.644320)
Supplement: Appendix 6 — Tool for identifying food provisioning policies and programmes. [file Table_6.DOCX]

**IDENTIFYING POLICIES AND PROGRAMMES**

**PROJECT TITLE: Measuring the Healthiness of Ghanaian Children's Food Environments to Prevent Obesity and Non-Communicable Diseases**

**DATA COLLECTION TOOL: School Food and/or Nutrition Policy/Programme Identification Tool**

Participant ID:

Date of interview |__||__|/|__||__|/|__||__||__||__|

Name of interviewer:

**Key Stakeholder Interview Guide**

**Name of Ministry/Department/Agency/Organisation:_____________________________________**

**Person interviewed: ___________________________ Role of the person interviewed: _______________________________**

**Section A – Identification of Food and Nutrition Policies/Programmes in Public Sector Schools**

***GOVERNMENT MINISTRIES, DEPARTMENTS AND AGENCIES – Ministries of Gender, Children and Social Protection, Local Government and Rural Development, Food and Agriculture, Education, Health, Ghana Education Service; Ghana Health Service, Ghana School Feeding Secretariat, Food and Drugs Authority, Ghana Standards Authority***

1. Existing public sector school food and nutrition policies/programmes
   1. *Are there any* ***policies*** *in place governing the nutritional quality of foods* ***provided*** *in public sector schools?* *What are they, at what levels of government are they applicable to (national, regional, district, community) and since when have these existed?*
   2. *Are there any general* ***policies*** *in place governing the nutritional quality of foods* ***sold*** *in public sector? (e.g.; those related to foods sold in school canteens)*

*What are they, at what level are they applicable to (national, regional, district, community), and since when have these existed?*

- 1. *Are there any* ***programmes*** *in place regulating the nutritional quality of foods* ***provided*** *in public sector schools (e.g. school food programmes)? What are they, at what level are they applicable to (national, regional, district, community), at what level of school (primary, secondary, post-secondary; school excursions, class parties, and school sponsored events such as fundraisings) are they applicable to and since when have these existed?*
  2. *Are there any* ***programmes*** *in place regulating the nutritional quality of foods* ***sold*** *in public sector schools (e.g. school food programmes)? (e.g.; those related to foods sold in school canteens)*

*What are they, at what level are they applicable to (national, regional, district, community), at what level of school (primary, secondary, post-secondary; school excursions, class parties, and school sponsored events such as fundraisings) are they applicable to and since when have these existed?*

1. Nutrition standards/guidelines applied to public sector school food and nutrition policies/programmes
   1. *Are there any existing* ***nutritional quality standards/guidelines*** *that could be used/adapted for application to nutritional quality of foods provided or sold by schools (e.g. national nutrition healthy eating guidelines, dietary or food guides, regulations governing nutrition labelling, nutrient content claims, health claims etc.)? Since when have these been in place?*
2. Legislative basis of nutrition policies/programmes/legislation/guidelines – ASK SPECIFIC AGENCIES eg. FDA, GSA

*3.1 Does your Ministry, Department or Agency have a policy/regulatory/legislative authority for regulating the nutritional quality of foods and beverages in public sector schools? If so, at what level (national, regional, district, community)?*

1. Financing of existing public sector school food and nutrition programmes.
   *4.1 Who finances the development and implementation of the identified policies/programmes?*
2. Key considerations made in making decisions around school food and nutrition policies/programmes

*5.1 Are there any key considerations when making decisions around what foods and beverages are provided or sold to children within public sector schools? If yes, what are they?*

1. Partners/organizations involved in the development of public sector school food and nutrition policies/programmes
   *6.1 Which other organisations (including other Government Ministries, Departments, and Agencies, NGOs, Civil Society, Academia) are involved in the development and implementation of the identified policies/programmes?*
2. Barriers and facilitators to implementing public sector school food and nutrition policies/programmes
   1. *What specific role does your Ministry/Department/Agency/Organization play in the development and/or implementation of the identified policies/programmes*
   2. *Are there any barriers to the implementation of the nutrition policies/programmes you are involved in?*
   3. *Are there any facilitators to the implementation of the nutrition policies/programmes you are involved in?*
3. Implementation support for schools participating in public sector school food programmes

*8.1 What resources are available to public sector schools participating in the identified policies/programmes?*

1. Accountability and compliance monitoring of public sector school food and nutrition policies/programmes
   1. *Is compliance with the identified policies/programmes and their associated nutrition standards/guidelines checked? If yes, how, and by whom?*
2. Other related public sector food and nutrition policies/programmes
   1. *Are there any* ***policies*** *governing the provision of* ***nutrition education*** *or the provision of nutrition curricula in public sector schools? What are they, at what level are they applicable to (national, regional, district, community), at what level of school (primary, secondary, tertiary) are they applicable to and since when have these existed?*
   2. *Are there any* ***programmes*** *in place relating to the* *provision of* ***nutrition education*** *in public sector schools (e.g. those for teachers and pupils)? What are they, at what level are they applicable to (national, regional, district, community), at what level of school (primary, secondary, tertiary) are they applicable to and since when have these existed?*
   3. *Are there any* ***food safety*** ***policies*** *addressing the safety of foods provided and/or sold in public sector schools* (e.g. *those regarding safe drinking water, food safety training/testing for staff and food handlers)? What are they, at what level are they applicable to (national, regional, district, community), at what level of school (primary, secondary, tertiary) are they applicable to and since when have these existed?*
   4. *Are there any* ***food safety*** ***programmes*** *in place relating to the* *provision of nutrition education in public sector schools* (e.g. *those regarding safe drinking water, food safety training for staff and food handlers)? What are they, at what level are they applicable to (national, regional, district, community), at what level of school (primary, secondary, tertiary) are they applicable to and since when have these existed?*

**QUESTIONS FOR PARTNERS OF THE GSFP AND OTHER NON-GOVERNMENTAL AND INTERNATIONAL ORGANISATIONS**

***World Food Programme WFP, Partnership for Child Development (PCD), WHO, UNICEF, FAO, HERSHEY GHANA, Cocoa Processing Company***

1. Public sector school food and nutrition **policies**
   *1.1 Are there any existing school nutrition policies which you have supported the development of and/or implementation of, or are aware of? What are they, at what level are they applicable to (national, regional, district, community), at what level of school (primary, secondary, post-secondary; school excursions, class parties, and school sponsored events such as fundraisings) are they applicable to and since when have these existed?*
2. Questions on existing school nutrition **programmes**
   *2.1 Are there any existing school nutrition programmes (e.g. school food programmes) which you have supported the development and/or implementation of, or are aware of? What are they, at what level are they applicable to (national, regional, district, community), at what level of school (primary, secondary, post-secondary; school excursions, class parties, and school sponsored events such as fundraisings) are they applicable to and since when have these existed?*
3. Key considerations and reasons for supporting the development and/or implementation of public sector school food and nutrition policies/programmes
   *3.1 What are your reasons for partnering in or supporting the development and/or implementation of the identified policies/programmes?*

**QUESTIONS FOR THE MINISTRY OF FINANCE AND ECONOMIC PLANNING**

1. Questions on existing policies/programmes related to the nutritional quality of foods sold or provided within publically-funded institutions? e.g.;
   1. *Are there any school food and nutrition* ***policies*** *governing the nutritional quality of foods* ***provided*** *in publically funded schools whose development and implementation the government of Ghana (GOG)? What are they, at what levels of government are they applicable to (national, regional, district, community) and since when have these existed?*
   2. *Are there any school food and nutrition* ***policies*** *governing the nutritional quality of foods* ***sold*** *in publically-funded schools whose development and implementation GOG finances? What are they, at what level are they applicable to (national, regional, district, community), and since when have these existed?*
   3. *Are there any school food and nutrition* ***programmes*** *regulating the nutritional quality of foods* ***provided*** *in publically-funded schools which GOG finances? What are they, at what level are they applicable to (national, regional, district, community), at what level of school (primary, secondary, post-secondary; school excursions, class parties, and school sponsored events such as fundraisings) are they applicable to and since when have these existed?*
   4. *Are there any school food and nutrition* ***programmes*** *regulating the nutritional quality of foods* ***sold*** *in publically-funded schools which GOG finances? What are they, at what level are they applicable to (national, regional, district, community), at what level of school (primary, secondary, post-secondary; school excursions, class parties, and school sponsored events such as fundraisings) are they applicable to and since when have these existed?*
2. Questions on key considerations made in making decisions around which public sector school food and nutrition policies/programmes to oversee the financing of

*2.1 Are they any key considerations made when making decisions around what food and nutrition policies and programmes to oversee the financing of?*
