# Supplementary material for: Providing Measurement, Evaluation, Accountability, and Leadership Support (MEALS) for Non-communicable Diseases Prevention in Ghana: Project Implementation Protocol
Source: Front Nutr. 2021 Aug 18;8:644320. doi: 10.3389/fnut.2021.644320 (PMC8416277; doi:10.3389/fnut.2021.644320)
Supplement: Appendix 7 — Policy-rating tool. [file Table_7.DOCX]

**POLICY RATING TOOL**

**PROJECT TITLE: Measuring the Healthiness of Ghanaian Children's Food Environments to Prevent Obesity and Non-Communicable Diseases**

**DATA COLLECTION TOOL: Food and Nutrition Policy Rating Tool**

Participant ID:

Date of interview |__||__|/|__||__|/|__||__||__||__|

Name of interviewer:

**For rating of existing school nutrition policies, programmes, or guidelines**

**Name of school: _______________________________________________________________________________________**

**Name of policy/programme/guideline: ____________________________________________________________________

 Country/Jurisdiction: ___________________________________________________________________________________ (e.g. Greater Accra, Ghana; Schools in Adenta Municipality)

 School sector: _________________________________________________________________________________________ (e.g. primary 1-6, all grades/classes)**

**Rating Guide**

**Instructions: Obtain a copy of the nutrition policy/programme and rate the details of the policy/programme against the listed items using the rating scale below;**

| **Rating** | | **Explanation** |
| --- | --- | --- |
| **0** | **Not mentioned** | The item is not included in the text of the policy. |
| **1** | **Weak statement** | \| Assign a rating of “1” when the item is mentioned, *but:*   - The policy will be hard to enforce because the statement is *vague*, *unclear*, or *confusing.* - Statements are listed as *goals*, *aspirations*, *suggestions,* or *recommendations.* - There are *loopholes* in the policy that weaken enforcement of the item. - The policy mentions a *future plan to act* without specifying when the plan will be established.   Words often used include*: may, can, could, should, might, encourage, suggest, urge, some, partial, make an effort,* and *try*. \| \| --- \| |
| **2** | **Meets/exceeds expectations** | Assign a rating of “2” when the item is mentioned, and it is clear that the policy makers are committed to making the item happen because:   - The item is described using specific language (e.g., a concept followed by concrete plans or strategies for implementation). - Strong language is used to indicate that action or regulation is required, including: *shall, will, must, have to, insist, require, all, total, comply* and *enforce.* - A district is unable to enforce an item (e.g., teachers’ role modelling healthy behaviours), but the goal is clearly stated (e.g., “shall encourage teachers to role model healthy behaviours”). |

| **Section 1: Nutrition Education/Nutrition Curricula** | | |
| --- | --- | --- |
| **Item no.** | **Item** | **Score** |
| **1.1** | Addresses the need for all participating schools to have a nutrition curriculum that includes relevant information on nutrition education |  |
| **1.2** | Nutrition education teaches skills that are behaviour-focused, interactive and participatory  e.g. score 1 for statements like "All students should have the skills necessary to make nutritious food choices” and 2 for statements like  "Nutrition education will incorporate lessons helping children acquire skills for reading food labels and menu planning."  "Schools will provide nutrition education lessons that cover topics such as reading a Nutrition Facts label." |  |
| **1.3** | Specifies that all primary schools receive sequential and comprehensive nutrition education |  |
| **1.4** | Specifies that all junior high schools receive sequential and comprehensive nutrition education |  |
| **1.5** | Nutrition education is integrated into other subjects beyond health education |  |
| **1.6** | Links nutrition education with the school food environment |  |
| **1.7** | Nutrition education addresses agriculture and the food system |  |
| **1.8** | Addresses the provision of nutrition education resources (such as posters, food pyramids, etc.) |  |
| **1.9** | Addresses organization of periodic nutrition training courses/workshops for teaching staff, and provides opportunities to practice skills |  |
| **Subtotal for Section 1** | **Comprehensiveness Score**: *Count the number of items rated as “1” or “2” and divide this number by 9. Multiply by 100. Do not count an item if the rating is “0.”* |  |
|  | **Strength Score**: *Count the number of items rated as “2” and divide this number by 9. Multiply by 100.* |  |

| **Section 2.: School food and nutrition programmes and school meals** | | |
| --- | --- | --- |
| **Item no.** | **Item** | **Score** |
| **2.1** | Specifies type of participation in the school food programme (rate 2 for mandatory participation) |  |
| **2.2** | Specifies the provision of healthful/nutritious school meals to all primary school students, if applicable |  |
| **2.3** | Specifies the provision of healthful/nutritious school meals to all junior high school students, if applicable |  |
| **2.4** | Food-based and/or nutrient based standards are applied to provided foods in the programme |  |
| **2.5** | Nutrition standards for provided foods are easily accessed (or a link to the standards is provided in the policy) |  |
| **2.6** | Details recommended serving sizes/proportions or quantities for different food groups (FOOD-BASED STANDARDS) OR provides details of nutrient thresholds (or exclusion and inclusion criteria) for specific nutrients (NUTRIENT-BASED STANDARD) OR both, if applicable |  |
| **2.7** | Nutrition standards apply to all provided foods in schools – e.g. foods provided in school canteens, cafeterias |  |
| **2.8** | Includes specific nutrition standards for specific age groups or grades |  |
| **2.9** | Addresses sanctions for non-compliance to nutrition standards |  |
| **2.10** | Addresses actions to be taken for safe, hygienic, and healthy purchasing of food ingredients |  |
| **2.11** | Addresses actions to be taken for safe, hygienic, and healthy food handling and preparation |  |
| **2.12** | Addresses periodic continuing food safety trainings for catering staff, teachers and pupils |  |
| **2.13** | Addresses preparedness for food emergencies (e.g. choking, medical emergencies such as food allergies) |  |
| **2.14** | Addresses provision of free, accessible, clean, and safe drinking water during school meals |  |
| **2.15** | Addresses the amount of ‘’seat time’’ pupils have to eat school meals |  |
| **2.16** | Addresses school meal environment/eating area (example: safety and cleanliness of eating areas) |  |
| **2.17** | Addresses students leaving school during lunch periods |  |
| **2.18** | Specifies strategies to improve participation in school meals  *e.g. score 1 for vague strategies such as "School meals shall be made attractive to students by appealing to their taste preferences." And 2 for specific strategies such as “The programme discourages consumption of competitive foods in place of school meals by limiting competitive food choices during mealtimes in the cafeteria.”* |  |
| **2.19** | Ensures annual nutrition training courses/workshops for catering personnel such as caterers, cooks, food vendors and other canteen staff and provides opportunities to practice skills |  |
| **2.20** | Addresses purchasing local foods for the school meal program |  |
| **2.21** | Nutrition information for provided meals (e.g., calories, saturated fat, sodium, sugar) is available to students and parents. |  |
| **Subtotal for Section 2** | **Comprehensiveness Score**: *Count the number of items rated as “1” or “2” and divide this number by 21. Multiply by 100. Do not count an item if the rating is “0.”* |  |
|  | **Strength Score**: *Count the number of items rated as “2” and divide this number by 21. Multiply by 100.* |  |

| **Section 3: Nutrition standards for sold and other competitive food and beverages** | | |
| --- | --- | --- |
| **Item no.** | **Item** | **Score** |
| **3.1** | Regulates all food and beverages sold to students during school hours – e.g. food and beverages sold in a la carte, vending machines, school stores, within and in the area around school canteens, cafeterias, as applicable |  |
| **3.2** | Regulates all food and beverages sold to students during extended school hours– e.g. foods sold during after school classes, programmes and clubs |  |
| **3.3** | Regulates all food and beverages sold to students after the school day, defined as 30 minutes after the final bell (including after care on school grounds, clubs, afterschool programming) |  |
| **3.4** | Food-based and/or nutrient based standards are applied to sold and competitive foods in the programme |  |
| **3.5** | Nutrition standards for sold food and other competitive food and beverages are easily accessed (or a link to the standards is provided in the policy) |  |
| **3.6** | Assures compliance with nutrition standards applied to all food and beverages sold to students during school hours – e.g. food and beverages sold in a la carte, vending machines, school stores, within and in the area around school canteens, cafeterias, as applicable |  |
| **3.7** | Assures compliance with nutrition standards applied to all foods sold to students during extended school hours – e.g. foods sold during after school classes, programmes and clubs |  |
| **3.8** | Assures compliance with nutrition standards for all BEVERAGES sold to students after the school day, defined as 30 minutes after the final bell (including after care on school grounds, clubs, afterschool programming) |  |
| **3.9** | Regulates food and beverages served at class parties and other school celebrations in primary and junior high schools. |  |
| **3.10** | Regulates food sold for fundraising at all times (not only during the school day). |  |
| **3.11** | Addresses sanctions for non-compliance |  |
| **3.12** | Addresses availability of free, accessible, clean, and safe drinking water throughout the school day |  |
| **3.13** | Addresses foods and beverages containing caffeine at the high school level |  |
| **Subtotal for Section 3** | **Comprehensiveness Score**: *Count the number of items rated as “1” or “2” and divide this number by 13. Multiply by 100. Do not count an item if the rating is “0.”* |  |
|  | **Strength Score**: *Count the number of items rated as “2” and divide this number by 13. Multiply by 100.* |  |

| **Section 4: Wellness promotion and food and beverage marketing** | | |
| --- | --- | --- |
| **Item no.** | **Item** | **Score** |
| **4.1** | Encourages staff to model healthy eating and physical activity behaviours |  |
| **4.2** | Addresses strategies to support employee wellness. |  |
| **4.3** | Addresses using physical activity as a reward |  |
| **4.4** | Addresses physical activity not being used as a punishment |  |
| **4.5** | Addresses physical activity not being withheld as a punishment |  |
| **4.6** | Specifies marketing to promote healthy food and beverage choices |  |
| **4.7** | Addresses marketing on signs, scoreboards, sports equipment |  |
| **4.8** | Addresses marketing in curricula, textbooks, websites used for educational purposes, or other educational materials (both printed and electronic) |  |
| **4.9** | Addresses marketing on exteriors of vending machines, food or beverage cups or containers, food display racks, coolers, trash and recycling containers, etc. |  |
| **4.10** | Addresses marketing on advertisements in school publications, on school radio stations, in-school television, computer screen savers, school-sponsored internet sites, or announcements on the public announcement (PA) system |  |
| **4.11** | Addresses marketing on fundraisers and corporate-sponsored programs that encourage students and their families to sell, purchase or consume products or provide funds to schools in exchange for consumer purchases of those products – including activities off campus; outside the school day; prohibiting anything not Smart Snacks; and prohibiting anything not Smart Snacks; and prohibiting posters or ordering catalogues for non-Smart Snacks, even if picked up after the school day |  |
| **Subtotal for Section 4** | **Comprehensiveness Score**: *Count the number of items rated as “1” or “2” and divide this number by 11. Multiply by 100. Do not count an item if the rating is “0.”* |  |
|  | **Strength Score**: *Count the number of items rated as “2” and divide this number by 11. Multiply by 100.* |  |

| **Section 5: Water, Sanitation and Hygiene** | | |
| --- | --- | --- |
| **Item no.** | **Item** | **Score** |
| **5.1** | Addresses the provision of clean, separate toilets for boys and girls |  |
| **5.2** | Addresses the provision of designated handwashing areas close to toilet facilities |  |
| **5.3** | Handwashing areas have running water and handwashing soap |  |
| **5.4** | Addresses provision of instructions for proper handwashing |  |
| **Subtotal for Section 5** | **Comprehensiveness Score**: *Count the number of items rated as “1” or “2” and divide this number by 4. Multiply by 100. Do not count an item if the rating is “0.”* |  |
|  | **Strength Score**: *Count the number of items rated as “2” and divide this number by 6. Multiply by 100.* |  |

| **Section 6: Implementation, Accountability, Monitoring and Evaluation, and Communication** | | |
| --- | --- | --- |
| **Item no.** | **Item** | **Score** |
| **7.1** | Establishes an ongoing implementing committee at the district level |  |
| **7.2** | District implementing committee has community-wide representation/Addresses how parents, students, representatives of the school food authority, school health professionals, the school board, school administrator, and the general public will participate in the development, implementation, and periodic review and update of the policy/programme |  |
| **7.3** | Addresses provision of implementation framework for all participating schools |  |
| **7.4** | Addresses provision of monitoring framework for all participating schools |  |
| **7.5** | Addresses provision of implementation support/tools for school administrators, principals, and/or teachers |  |
| **7.6** | Addresses provision of monitoring and evaluation support/tools for cafeteria workers, food suppliers etc. |  |
| **7.7** | Addresses provision of implementation support/tools for cafeteria workers, food suppliers etc. |  |
| **7.8** | Addresses provision of monitoring and evaluation support/tools for school administrators, principals, and/or teachers |  |
| **7.9** | Addresses designation of school officials responsible for the implementation and oversight of the local policy/programme to ensure each school’s compliance |  |
| **7.10** | Addresses the assessment of district implementation of the policy/programme at least once every three years |  |
| **7.11** | Triennial assessment results on compliance/implementation is made available to the public and will include  A. the extent to which schools under the jurisdiction of the LEA are in compliance with the local school wellness policy;  B. the extent to which the LEA’s local school wellness policy compares to model local school wellness policies;  C. description of the progress made in attaining the goals of the local school wellness policy |  |
| **7.12** | Addresses a plan for updating policy based on results of the triennial assessment |  |
| **Subtotal for Section 7** | **Comprehensiveness Score**: *Count the number of items rated as “1” or “2” and divide this number by 12. Multiply by 100. Do not count an item if the rating is “0.”* |  |
|  | **Strength Score**: *Count the number of items rated as “2” and divide this number by 12. Multiply by 100.* |  |

**SUMMARY OF SCORES**

| **SECTION** | **Comprehensiveness Score** | **Strength Score** |
| --- | --- | --- |
| 1. Nutrition Education/Nutrition Curricula |  |  |
| 1. School food and nutrition programmes and school meals |  |  |
| 1. Nutrition standards for sold and other competitive foods and beverages |  |  |
| 1. Wellness promotion and food and beverage marketing |  |  |
| 1. Water, Sanitation and Hygiene |  |  |
| 1. Implementation, Monitoring and Evaluation, and Communication |  |  |
| **OVERALL COMPREHENSIVENES =** (total number of items in ALL sections receiving a “1”or “2” /70) x 100)  **______________________** | | |
| **OVERALL STRENGTH =** (total number of items in ALL sections receiving a “2” /70) x 100)  **______________________** | | |
