# Supplementary material for: Providing Measurement, Evaluation, Accountability, and Leadership Support (MEALS) for Non-communicable Diseases Prevention in Ghana: Project Implementation Protocol
Source: Front Nutr. 2021 Aug 18;8:644320. doi: 10.3389/fnut.2021.644320 (PMC8416277; doi:10.3389/fnut.2021.644320)
Supplement: Appendix 8 — IDI guide for school heads and administrators. [file Table_8.DOCX]

**IDI GUIDE - SCHOOL HEADS AND ADMINISTRATORS**

**PROJECT TITLE: Measuring the Healthiness of Ghanaian Children's Food Environments to Prevent Obesity and Non-Communicable Diseases**

**DATA COLLECTION TOOL: School Food and Nutrition Policy (In-depth Interviews)**

Participant ID:

Date of interview |__||__|/|__||__|/|__||__||__||__|

Name of interviewer:

**School questionnaire**

**Name of School:
Person being Interviewed: School Head [ ] Assistant School Head [ ] School Administrator [ ] Other (specify):_________________**

**Name of school: _____________________________________ Date of interview:___________________________**

**Questions for School Heads/Assistant school heads/Administrators**

1. **School statistics**

| **No** | **Question** | **Answer options** |
| --- | --- | --- |
| 1.1 | In what **year** was the school established? | / / / |
| 1.2 | Levels available in school --- ***Tick relevant option(s)*** | 1. Primary [ ] 2. JHS [ ] 3. Both [ ] |
| ***Questions 1.3 to 1.7 only apply to schools that have a Primary section. Complete if applicable*** | | |
| 1.3 | Age range of pupils (**Primary**) | ___ to ___ (years) |
| 1.4 | Sex distribution of **Primary** pupils (provide percentage if exact numbers are not available) | Boys:  Girls:  Do not know [ ] |
| 1.5 | Total number of **Primary** pupils | [ ] [ ] [ ] [ ]  Do not know [ ] |
| 1.6 | Average number of pupils per class (**Primary**) | [ ] [ ]    Do not know [ ] |
| 1.7 | Number of teachers in school (**Primary**) | [ ] [ ]  Do not know [ ] |
| ***Questions 1.8 to 1.12 only apply to schools that have a JHS section. Complete if applicable*** | | |
| 1.8 | Age range of pupils (**JHS**) | ___ to ___ (years) |
| 1.9 | Sex distribution of **JHS** pupils (provide percentage if exact numbers are not available) | Boys:  Girls:  Do not know [ ] |
| 1.10 | Total number of **JHS** pupils | [ ] [ ] [ ] [ ]    Do not know [ ] |
| 1.11 | Average number of pupils/class (**JHS**) | [ ] [ ]  Do not know [ ] |
| 1.12 | Number of teachers in school (**JHS**) | [ ] [ ]  Do not know [ ] |
| 1.13 | Is there a committee responsible for health or nutrition in your school? | 1. Yes [ ] 2. No [ ] |

1. **Nutrition knowledge/awareness**

| **No** | **Question** | **Answer options** |
| --- | --- | --- |
| 2.1 | In your opinion, what do you consider as healthy food?  *(Qualitative open-ended question, RA to* ***probe for examples****)* | |
| 2.2 | On what basis/guideline is your definition for a healthy food?  *(Qualitative open-ended question, RA to probe for details)* | |
| 2.3 | In your opinion, what do you consider as unhealthy food?  *(Qualitative open-ended question, RA to* ***probe for examples****)* | |
| 2.4 | On what basis/guideline is your definition for an unhealthy food?  *(Qualitative open-ended question, RA to* ***probe for details****)* | |
| 2.5 | In your opinion, what do you consider as safe food?  *(Qualitative open-ended question, RA to* ***probe for examples****)* | |
| 2.6 | On what basis/guideline is your definition for a safe food?  *(Qualitative open-ended question, RA to* ***probe for details****)* | |
| 2.7 | How important is it to you, that your staff and pupils consume healthy foods?? (Rate from 1 to 10, 1 being least important, 10 being most important) | [ ] [ ] |

**Instructions:**

The following sections are on school food and nutrition programmes/policies regulating the provision and/or sale of foods in and around schools, and those regulating the marketing of foods and beverages in and around schools. Ask for the appropriate person to be interviewed for each section (for example, other members of the GSFP School implementing Committee). The school head may provide responses to all sections if he/she is in a position to do so.

1. **School food and nutrition policies/programmes (Provided Foods)**

Person being Interviewed:

| **No** | **Question** | **Answer options** |
| --- | --- | --- |
| 3.1 | Does your school have a school food and/or nutrition programme that has a food provision component? ***If no, skip to section 4*** | 1. Yes [ ] 2. No [ ] |
| 3.2 | What is the name of the programme? | |
| 3.3 | Is there a school-level document of the programme? ***(If yes, obtain a copy of this)*** | 1. Yes [ ] 2. No [ ] |
| 3.4 | Is this programme different from the Ghana School Feeding Programme (GSFP)? | 1. Yes [ ] 2. No [ ] |
| 3.4a | If so, how does it fit with the GSFP?  *(Qualitative open-ended question, RA to* ***probe for details****)* | |
| 3.5 | Has the programme on provided foods/drinks been/being implemented? ***If no, skip 3.3b*** | 1. Yes [ ] 2. No [ ] |
| 3.5a | If yes, when was the implementation started? | Month: ________  Year: _________ |
| 3.6 | Do you have a school implementing committee for the programme? | 1. Yes [ ] 2. No [ ] |
| 3.7 | Which classes, participate in the programme?  ***Circle the correct answer*** | Primary: 1 2 3 4 5 6 ALL  JHS: 1 2 3 ALL |
| 3.8 | Approximately, how many pupils, in total, participate in the programme?  *(Provide a percentage if exact number is not known)* | #: _________  OR  %:_________ |
| 3.9 | Where do pupils who do not participate in the programme obtain their meals from? | 1. Food vendors [ ] 2. Home [ ] 3. Other (specify)_______ 4. Do not know [ ] 5. N/A [ ] |
| 3.10 | What type of meals does the programme provide?  ***Tick all that apply*** | 1. Breakfast [ ] 2. Lunch [ ] 3. Snack [ ] 4. Other (specify)______ |
| 3.11 | Do you (respondent) have the opportunity to contribute to the decision-making process around what foods are provided to children participating in the programme?  ***If no, skip question 3.9*** | 1. Yes [ ] 2. No [ ] |
| 3.12 | Does the school have the opportunity to contribute to the decision-making process around what foods are provided to children participating in the programme?  ***If no, skip question 3.9*** | 1. Yes [ ] 2. No [ ] |
| 3.13 | Are there any key considerations in making decisions around what foods are provided to children participating in the programme?  ***If no, skip 3.13a*** | 1. Yes [ ] 2. No [ ] |
| 3.13a | What are the key considerations in making decisions on food provided to children participating in the programme?  *(Qualitative open-ended question, RA to* ***probe for details****)* | |
| 3.14 | In your opinion, are the foods/drinks provided healthy for pupils? | 1. Yes [ ] 2. No [ ] |
| 3.14a | Why do you think the foods/drinks provided are healthy (or unhealthy) for pupils?  *(Qualitative open-ended question, RA to* ***probe for details****)* | |
| 3.15 | In your opinion, are the foods/drinks provided safe for pupils? | 1. Yes [ ] 2. No [ ] |
| 3.15a | Why do you think the foods/drinks provided are safe (or unsafe) for pupils?  *(Qualitative open-ended question, RA to* ***probe for details****)* | |
| 3.16 | Are there any nutrition standards/guidelines applied to the programme? *(for example, standards for what types of foods should or should not be provided)*  ***If no, skip 3.16a to 3.17*** | 1. Yes [ ] 2. No [ ] |
| 3.16a | If yes, what are the nutrition standards/guidelines?  *(Qualitative open-ended question, RA to* ***probe for details****)* | |
| 3.16b | How frequently are these nutrition standards/guidelines updated? | |
| 3.17 | On a scale of 1-5 (1 being ‘non-compliant’ and 5 being ‘fully compliant’, how would you rate your school’s overall compliance with the nutrition standards/guidelines of the policy/programme? | 1. Non-compliant 2. Not very compliant 3. Somewhat compliant 4. Mostly compliant 5. Fully compliant 6. Do not know about the nutrition standards/guidelines 7. Do not understand what is in the nutrition standards/guidelines |
| 3.18 | What actions has the school taken, if any, to address deviations from the requirements of the school food programme?  *(Qualitative open-ended question, RA to* ***probe for details****)* | |
| 3.19 | Does your school have a monitoring plan or framework developed for the monitoring of the programme? | 1. Yes [ ] 2. No [ ] |
| 3.20 | Has the programme ever been monitored?  ***If no, skip to questions 3.20a to 3.20d*** | 1. Yes [ ] 2. No [ ] |
| 3.20a | If yes, when was the latest monitoring done? | Month: __________  Year: ___________ |
| 3.20b | Who conducted the latest monitoring? | 1. School officials 2. GSFP 3. Third party (independent of GSFP or school officials) 4. Other (specify)   ________________ |
| 3.20c | When was the latest monitoring done? | Month: _________  Year: ___________ |
| 3.20d | Do you have available, latest monitoring data or a monitoring report of the programme?  ***(Ask for a copy of this to be reviewed, if available)*** | 1. Yes [ ] 2. No [ ] |
| 3.21 | Has the implementation of the school food programme led to any **expected changes** (positive or negative) in the school canteen? (e.g. more cleaner eating area, availability of healthier food options, pupils limiting purchase of competitive foods/drinks)  *(Qualitative open-ended question, RA to* ***probe for details****)* | |
| 3.22 | Has the implementation of the school food programme led to any **unexpected changes** (positive or negative) in the school canteen? (e.g. more cleaner eating area, availability of healthier food options, pupils limiting purchase of competitive foods/drinks)  *(Qualitative open-ended question, RA to* ***probe for details****)* | |
| 3.23 | Have there been any changes to your canteen or school in the last year that may have had an impact on the implementation of the programme?  *(For example, change in catering staff; major refurbishment of canteen facility; change in school administration; received donations from individuals or organisations, provision of school kitchen)*  *(Qualitative open-ended question, RA to* ***probe for details****)* | |
| 3.24 | What do you think, in your opinion, has helped (facilitators) the implementation of the school feeding programme in your school?  *(Qualitative open-ended question, RA to* ***probe for details****)* | |
| 3.25 | What do you think has hindered (barriers) the implementation of the school feeding programme in your school?  *(Qualitative open-ended question, RA to* ***probe for details****)* | |

1. **School food and nutrition policies/programmes (Sold Foods)**

Person being Interviewed:

| **No** | **Question** | **Answer options** | |
| --- | --- | --- | --- |
| 4.1 | Do students bring their own food to school?  ***If no or do not know, skip 4.1a*** | 1. Yes [ ] 2. No [ ] 3. Do not know [ ] | |
| 4.1a | If yes, roughly how many bring food to school? (in percentage | #: __________  OR  %: __________ | |
| 4.2 | Are there ***food vendors* located** within the school compound?  ***If no, end section 4 here*** | 1. Yes [ ] 2. No [ ] | |
| 4.3 | At what times do the food vendors operate? | 1. Morning 2. Afternoon 3. The whole school day 4. Other *(specify)*_____ | |
| 4.4 | Do food vendors have permission from you (school authorities) to sell food within the school compound? | 1. Yes [ ] 2. No [ ] | |
| 4.5 | What is the process for granting this permission?  *(Qualitative open-ended question, RA* ***to probe for details)*** | | |
| 4.6 | Roughly how many pupils buy food from food vendors within the school compound? (Provide percentage if exact number is not known) | #: ____________  OR  %: ___________ | |
| 4.7 | In your opinion, are the foods/drinks sold by the vendors within the school compound **healthy** for pupils? | 1. Yes [ ] 2. No [ ] | |
| 4.7a | Why do you think the foods/drinks sold by the vendors within the school compound are **healthy** for pupils? (PROBE)  *(Qualitative open-ended question, RA* ***to probe for details)*** |  |  |
| 4.8 | In your opinion, are the foods/drinks sold by the vendors within the school compound **safe** for pupils? | 1. Yes [ ] 2. No [ ] | |
| 4.8a | Why do you think the foods/drinks sold by the vendors within the school are compound **safe** for pupils?  *(Qualitative open-ended question, RA* ***to probe for details)*** | | |
| 4.9 | Do you (respondent) have the opportunity to contribute to the decision-making process around what foods are sold to pupils within your school compound?  *If no, skip question 4.8* | 1. Yes [ ] 2. No [ ] | |
| 4.10 | Does the school have opportunity to contribute to the decision-making process regarding foods sold to pupils within your school compound? | 1. Yes [ ] 2. No [ ] | |
| 4.11 | Are there any key considerations or guidelines in making decisions around foods that are sold to children pupils within your school compound? | 1. Yes [ ] 2. No [ ] | |
| 4.11a | What are the key considerations or guidelines in making decisions around what foods are sold to children pupils within your school compound?  *(Qualitative open-ended question, RA* ***to probe for details****)* | | |
| 4.12 | Do you have any written policy/guidelines regarding foods/drinks that can be sold within the school compound? *(If yes, obtain a copy of this)* | 1. Yes [ ] 2. No [ ] | |
| 4.13 | Has the policy/guideline on foods/drinks sold on the compound been/being implemented? *If no, skip 4.13a* | 1. Yes [ ] 2. No [ ] | |
| 4.13a | When was the policy/regulation implemented? | Month: ________  Year: _________ | |
| 4.14 | Are there any nutrition standards/guidelines applied to the policy/regulation?  ***If no, skip 4.14a*** | 1. Yes [ ] 2. No [ ] | |
| 4.14a | How frequently are these nutrition standards/guidelines updated? |  | |
| 4.15 | On a scale of 1-5 (1 being ‘non-compliant’ and 5 being ‘fully compliant’, how would you rate your school’s overall compliance with the policy/regulation? | 1. Non-compliant 2. Not very compliant 3. Somewhat compliant 4. Mostly compliant 5. Fully compliant 6. Do not know of the standard 7. Do not understand/ the standard | |
| 4.16 | What actions have you taken, if any to address deviations from the requirements of the policy/regulation?  *(Qualitative open-ended question, RA to* ***probe for details****)* | | |
| 4.17 | Does your school have a monitoring plan or framework developed for the monitoring of sold foods/drinks? | 1. Yes [ ] 2. No [ ] | |
| 4.18 | Has the programme ever been monitored?  ***If no, skip 4.18a to 4.18d*** | 1. Yes [ ] 2. No [ ] | |
| 4.18a | If yes, when was the latest monitoring done? | Month: ________  Year: ________ | |
| 4.18b | Who conducted the latest monitoring? | 1. School officials 2. GSFP 3. Third party (independent of GSFP or school officials) 4. Other (specify)   _____________ | |
| 4.18c | When was the latest monitoring done? | Month: ________  Year: ________ | |
| 4.18d | Do you have available monitoring data or a monitoring report from the latest monitoring of the policy/regulation? *(Ask for a copy of this to be reviewed, preferably a third-party review report* ***of the programme****)* | 1. Yes [ ] 2. No [ ] | |
| 4.19 | Has the implementation of the policy/regulation on sold foods/drinks led to any **expected changes** (positive or negative) in the school canteen? (e.g. more cleaner eating area, availability of healthier foods/drinks)  *(Qualitative open-ended question, RA to probe for details)* | 1. Yes [ ] 2. No [ ] | |
| 4.20 | Has the implementation of the policy/regulation on sold foods/drinks led to any **unexpected changes** (positive or negative) in the school canteen? (e.g. more cleaner eating area, availability of healthier foods/drinks)  *(Qualitative open-ended question, RA to probe for details)* | 1. Yes [ ] 2. No [ ] | |
| 4.21 | Have there been any changes to your canteen or school in the last year that may have had an impact on the implementation of the sold food policy/regulation? (For example, major refurbishment of canteen facility; change in school administration; change in food vendors)  *(Qualitative open-ended question, RA to* ***probe for details****)* | 1. Yes [ ] 2. No [ ] | |
| 4.22 | What do you think, in your opinion, has helped (facilitators) the implementation of the policy/regulation on sold foods/drinks in your school?  *(Qualitative open-ended question, RA to* ***probe for details****)* | | |
| 4.23 | What do you think has hindered (barriers) the implementation of the policy/regulation on sold foods/drinks in your school?  *(Qualitative open-ended question, RA to* ***probe for details****)* | | |

1. **School food and nutrition policies/programmes (Marketing)**
   Person being Interviewed:

| **No** | **Question** | **Answer options** |
| --- | --- | --- |
| 5.1 | In your opinion, what is the extent of exposure of pupils in your school to unhealthy food marketing and advertising in and around your school? Would you say they are/have…..***(read options)*** | 1. No exposure 2. Limited exposure 3. Somewhat exposed 4. Mostly exposed 5. Highly/excessively exposed 6. Do not know |
| 5.2 | Explain your answer above  *(Qualitative open-ended question, RA to* ***probe for details****)* | |
| 5.3 | Does your school have any policy/guideline regulating food and beverage marketing (e.g. those restricting school food and beverage marketing) within your school compound? ***If yes, obtain a copy of this to be reviewed*** | 1. Yes [ ] 2. No [ ] |
| 5.4 | Has the policy/guideline been/being implemented? When was it implemented? | 1. Yes [ ] 2. No [ ] |
| 5.5 | When was the last time this policy/guideline was revised/updated? | |
| 5.6 | How do you ensure compliance to the policy/guideline?  *(Qualitative open-ended question, RA to* ***probe for details****)* | |
| 5.7 | Do you receive sponsorships from any food and beverage companies for programs, including scholarships, school events and fundraisings? *If no, skip 5.7a* | 1. Yes [ ] 2. No [ ] |
| 5.7a | If yes, which companies? | |
| 5.8 | Do pupils and/or staff use any curriculum, materials (such as textbook covers, equipment and supplies), or classroom activities created/sponsored by food or beverage companies or associated organizations?  ***If no, skip 5.8a*** | 1. Yes [ ] 2. No [ ] |
| 5.8a | If yes, which companies sponsor these? | |
| 5.9 | On a scale of 1-5 (1 being ‘non-compliant’ and 5 being ‘fully compliant’, how would you rate your school’s overall compliance with the nutrition standards/guidelines of the policy/programme? | 1. Non-compliant 2. Not very compliant 3. Somewhat compliant 4. Mostly compliant 5. Fully compliant 6. Do not understand the nutrition standards/guidelines |

**Further Comments**

| Is there anything else you would like to say? |
| --- |

***Thank you for your time***
