# Supplementary material for: Providing Measurement, Evaluation, Accountability, and Leadership Support (MEALS) for Non-communicable Diseases Prevention in Ghana: Project Implementation Protocol
Source: Front Nutr. 2021 Aug 18;8:644320. doi: 10.3389/fnut.2021.644320 (PMC8416277; doi:10.3389/fnut.2021.644320)
Supplement: Appendix 9 — IDI guide for caterers. [file Table_9.DOCX]

**School Caterer Interview**

**PROJECT TITLE: Measuring the Healthiness of Ghanaian Children's Food Environments to Prevent Obesity and Non-Communicable Diseases**

Participant ID:

Date of interview |__||__|/|__||__|/|__||__||__||__|

Name of interviewer:

**Food Provider Questionnaire**

**Name of school:_____________________________________ Date of interview:___________________________**

**Name of school/school code:**

**Person being interviewed: Caterer [ ] Head Cook [ ]**

1. **Background characteristics**

| **No** | **Question** | **Answer options** |
| --- | --- | --- |
| 1.1 | Sex of respondent | 1. Male 2. Female |
| 1.2 | Age in completed years | ______ years |
| 1.3 | Level of education | 1. No formal education 2. Primary 3. JHS 4. SHS 5. Tertiary 6. Other *(specify)* |
| 1.4 | Length of time working in food service *(in years)* | ______ years |
| 1.5 | Length of time working in food service (specifically for school food programmes) *(in years)* | ______ years |
| 1.6 | Who contracted you to provide foods for the school food programme? | 1. GSFP officials 2. School authorities 3. Other *(specify)* |
| 1.7 | Which schools do you provide foods for?  *(mention names of schools)* | School 1: ______  School 2: ______  School 3: ______ |
| 1.8 | Have you received any food certification from a regulatory body? *If no, skip questions 1.8 to 1.10* | 1. Yes 2. No |
| 1.9 | If yes, to question 1.7, indicate what type of certification you received from the regulatory body | Certification 1: ____  Certification 2: ____  Certification 3: ____ |
| 1.10 | From which regulatory body did you receive each certification? | Regulatory body (Certification 1): ______________  Regulatory body (Certification 2): ______________  Regulatory body (Certification 3): ______________ |
| 1.11 | When did you receive the certification(s) from the regulatory body? | Month/Year (Certification 1): ______________  Month/Year (Certification 2): ______________  Month/Year (Certification 3): ______________ |

1. **Nutrition knowledge**

| **No** | **Question** | | **Answer options** |
| --- | --- | --- | --- |
| 2.1 | In your opinion, what do you consider as healthy food?  *(Qualitative open-ended question,* ***RA to probe for details****)* | | |
| 2.2 | On what basis/guideline is your definition for a healthy food?  *(Qualitative open-ended question,* ***RA to probe for details****)* | | |
| 2.3 | In your opinion, what do you consider as unhealthy food?  *(Qualitative open-ended question,* ***RA to probe for details****)* | | |
| 2.4 | On what basis/guideline is your definition for an unhealthy food?  *(Qualitative open-ended question,* ***RA to probe for details****)* | | |
| 2.5 | Do you receive food/and nutrition education or training? *If no, skip questions 2.5a-2.5d* | 1. Yes 2. No | |
| 2.5a | Who provides the food and/or nutrition education or training? *(Select all that apply)* | 1. School authorities 2. GSFP officials 3. Other *(specify)* | |
| 2.5b | Is the food and/or nutrition training a one-time training or do you have regular training sessions? *If one-time, skip 2.8.* | 1. One-time 2. Routine | |
| 2.5c | If answered 2 (routine) to 2.7, how often do you receive food and/or nutrition education or training? | 1. Yearly 2. Half-yearly 3. Quarterly 4. Monthly 5. Weekly | |
| 2.5d | What topics are covered in the food and/or nutrition education or training? *(Tick all applicable)* | 1. Food groups 2. Healthy/nutritious meal preparation 3. Food safety 4. Food hygiene and sanitation 5. Food quality 6. Other *(specify)* | |

1. **School food programmes (Awareness and compliance to applied nutrition standards/guidelines)**

| **No** | **Question** | | | |
| --- | --- | --- | --- | --- |
| 3.1 | What foods did you serve to pupils on the following days? *(1-week recall of actual foods served to pupils. RA should recall from the school day preceding the survey)*  Monday  Tuesday  Wednesday  Thursday  Friday | | | |
| 3.2 | For each school meal served in the school days preceding the survey, please mention the foodstuffs/ingredients used in their preparation  Monday meal (Foodstuffs/ingredients used)  Tuesday meal (Foodstuffs/ingredients used)  Wednesday meal (Foodstuffs/ingredients used)  Thursday meal (Foodstuffs/ingredients used)  Friday meal (Foodstuffs/ingredients used) | | | |
| 3.3 | For each school meal served in the school days preceding the survey, please describe how it was prepared *(RA to* ***probe for measurements used****)*  Monday meal (how food was prepared)  Tuesday meal (how food was prepared)  Wednesday meal (how food was prepared)  Thursday meal (how food was prepared)  Friday meal (how food was prepared) | | | |
| 3.4 | Do you have a 1-week menu of the provided school meals available?  *(If yes, RA to obtain 1-week menu of the school food programme and take a photograph of the menu for review)* | | | |
| 3.5 | Are there any instances where you have been unable to provide school meals during the school day? Please provide details  *(Qualitative open-ended question, RA to* ***probe for details****)* | | | |
| 3.6 | Are you provided with any guidelines/instructions (either by the school or government) on what foodstuffs you should use for the preparation of school meals? *If no, skip 3.7-3.9* | | | 1. Yes 2. No |
| 3.7 | What do the guidelines/instructions say?  *(Qualitative open-ended question, RA to* ***probe for details****)* | | | |
| 3.8 | How easy/difficult is it to follow these guidelines/instructions? Please explain your answer  *(Qualitative open-ended question, RA to* ***probe for details****)* | | | |
| 3.9 | On a scale of 1-5 (1 being ‘non-compliant’ and 5 being ‘fully compliant’, how would you rate the catering staff’s overall compliance to the guidelines/instructions? | 1. Non-compliant 2. Not very compliant 3. Somewhat compliant 4. Mostly compliant 5. Fully compliant | | |
| 3.10 | Are you provided with any guidelines/instructions (either by the school or government) on how you should prepare school meals? *If no, skip 3.11-3.13*  *(* | 1. Yes 2. No | | |
| 3.11 | If yes, what do the guidelines/instructions say?  *Qualitative open-ended question, RA to* ***probe for details****)* | | | |
| 3.12 | How easy/difficult is it to follow these guidelines/instructions? Please explain your answer  *(Qualitative open-ended question, RA to* ***probe for details****)* | | | |
| 3.13 | On a scale of 1-5 (1 being ‘non-compliant’ and 5 being ‘fully compliant’, how would you rate the catering staff’s overall compliance to the guideline/instruction? | 1. Non-compliant 2. Not very compliant 3. Somewhat compliant 4. Mostly compliant 5. Fully compliant | | |
| 3.14 | Are there any available resources/materials to help you and the other catering staff to understand the guidelines/instructions, as well as what is required of you? *If no, skip 3.15*  *(* | 1. Yes 2. No | | |
| 3.15 | How easy/difficult is it to use these resources/materials?  *(Qualitative open-ended question, RA to* ***probe for details****)* | | | |
| 3.16 | Are you taking any steps to ensure the healthiness of the food and beverages that you provide? *If no, skip 3.17* | 1. Yes 2. No | | |
| 3.17 | What steps to ensure the healthiness of the food and beverages that you provide?  *(Qualitative open-ended question, RA to* ***probe for details****)* | | | |
| 3.18 | What is the cost of each meal you provide per child?  *(Qualitative open-ended question, RA to* ***probe for details****)* | | | |
| 3.19 | In your opinion, is the cost of the meal sufficient to meet the **nutritional needs** of each child? Please explain your answer  *(Qualitative open-ended question, RA to* ***probe for details****)* | | | |
| 3.20 | In your opinion, is the cost of the meal sufficient to provide the needed **quantity** of school meals for each child? Please explain your answer  *(Qualitative open-ended question, RA to* ***probe for details****)* | | | |
| 3.21 | Do you receive any subsidies (extra payments) for the meals you provide? *If no, skip 3.22* | | 1. Yes 2. No | |
| 3.22 | If yes, how much do you receive per child?  *(Qualitative open-ended question, RA to* ***probe for details****)* | | | |

1. **Food Safety, Water, Sanitation and Hygiene**

| **No** | **Question** | |
| --- | --- | --- |
| 4.1 | Where do you prepare the school meals?  *If answered 1 or 2, skip 4.2 and 4.3* | 1. In a kitchen facility on the school premises 2. In an open space on the school premises 3. In a professional kitchen outside school facility 4. At home 5. Other *(specify)* |
| 4.2 | 1. If food is prepared outside the school facility, how is it transported to the school canteen/designated eating area?   *(Qualitative open-ended question, RA to* ***probe for details****)* | |
| 4.3 | 1. If food is prepared outside the school facility in what container is it transported to the school canteen/designated eating area?   *(Qualitative open-ended question, RA to* ***probe for details****)* | |
| 4.4 | Do you have access to a toilet facility near the cooking facility? *If no, skip 4.5 and 4.6* | 1. 1. Yes 2. 2. No |
| 4.5 | Does the toilet facility have water available for hand washing? | 1. Yes, running water from public supply system 2. Yes, running water from plastic tanks (with or without tap) 3. Yes, water in buckets 4. No |
| 4.6 | Is there soap/hand sanitizer available at the toilet facility? | 1. Yes 2. No |
| 4.7 | Do you have access to a toilet facility near the serving/eating area? *If no, skip 4.8 and 4.9* | 1. Yes 2. No |
| 4.8 | Does the toilet facility have water available for hand washing? | 1. Yes, running water from public supply system 2. Yes, running water from plastic tanks (with or without tap) 3. Yes, water in buckets 4. No |
| 4.9 | Is there soap/hand sanitizer available at the toilet facility? | 1. Yes 2. No |
| 4.10 | How would you describe the facility where you prepare the meals *(probe for equipment available, cleanliness, etc.)*  *(Qualitative open-ended question, RA to* ***probe for details****)* | |

**Field worker independent assessment of school canteen/designated eating area**

1. Take a photograph of the eating area and provide a detailed assessment of the school canteen/designated eating area by describing the following;

| **No** | **Question** | **Answer options** |
| --- | --- | --- |
| 5.1 | Where are the school meals being served? | 1. Classroom(s) 2. Designated eating area that is completely enclosed 3. Designated eating area that is partially enclosed 4. Designated eating area in an open space |
| 5.2 | Does the serving/eating area have a roof / cover? | 1. Yes, completely roofed/covered 2. Yes, partially roofed/covered 3. No |
| 5.3 | On a scale of 1 to 10, 1 being very clean, and 10 being very dirty, how would you rate the cleanliness of the serving/eating area (observe walls and floor)?  *If no, skip 5.5* | 1 2 3 4 5 6 7 8 9 10 |
| 5.4 | Are there seats/tables available for children to sit and eat? | 1. Yes 2. No |
| 5.5 | On a scale of 1 to 10, 1 being very clean, and 10 being very dirty, how would you rate the cleanliness of the available seats and/or tables? | 1 2 3 4 5 6 7 8 9 10 |
| 5.6 | Are plates/bowls and other cutlery provided for the children? | 1. Yes, disposable cutlery 2. Yes, non-disposable cutlery 3. No cutlery provided (i.e. pupils bring their own cutlery) 4. Not applicable |
| 5.7 | Is there water for washing available at serving/eating point? | 1. Yes, running water from public supply system 2. Yes, running water from plastic tanks (with or without tap) 3. Yes, water in buckets (clean water) 4. Yes, water in buckets (dirty water) 5. No water |
| 5.8 | Is there soap/hand sanitizer available at serving/eating point? | 1. Yes 2. No 3. Not applicable |
| 5.9 | Non-disposable cutlery washed with | 1. With running water and soap 2. With running water only 3. In buckets (clean water) 4. In buckets (dirty water) 5. no washing 6. Cannot tell 7. Not applicable |
| 5.10 | Is the provided food kept less than 1 meter from the ground | 1. Yes 2. No |
| 5.11 | Is the provided food adequately protected from flies and dust? | 1. Yes 2. Partially 3. No |
| 5.12 | Is there a rubbish bin available at the serving/eating site? *If no, skip 1.13* | 1. Yes 2. No |
| 5.13 | Briefly describe the state of the rubbish bin (covered, overfilled, open and overfilled, etc.) |  |
| 5.14 | Are there any advertisements/signs/posts promoting healthy food consumption on site? | 1. Yes 2. No |
| 5.15 | Are there any food advertisements/signs/posts on site? | 1. Yes 2. No |

1. Note down every single dish and drink **provided** on day of visit. (e.g. Yam, kontonmire stew, and smoked fish provided or kenkey, ground pepper and fried fish provided)
2. Take photograph of served meal

***Thank you for your time***
