# Supplementary material for: Providing Measurement, Evaluation, Accountability, and Leadership Support (MEALS) for Non-communicable Diseases Prevention in Ghana: Project Implementation Protocol
Source: Front Nutr. 2021 Aug 18;8:644320. doi: 10.3389/fnut.2021.644320 (PMC8416277; doi:10.3389/fnut.2021.644320)
Supplement: Appendix 10 — M & E review tool. [file Table_10.DOCX]

**PROJECT TITLE: Measuring the Healthiness of Ghanaian Children's Food Environments to Prevent Obesity and Non-Communicable Diseases**

**DATA COLLECTION TOOL: Review of Available Monitoring Data**

Date of observation:|__||__|/|__||__|/|__||__||__||__| Time of observation:

Name of interviewer:

**For assessment in schools that have a monitoring framework and available monitoring data**

**Name of school: _____________________________________ Date of interview: ___________________________**

**Name of policy/programme: ______________________________________________________________________________________________**

**Date of implementation of policy/programme: ________________________________________________________________________________**

**Date of monitoring: ______/________/_________**

**Independent document review by researcher**

**Component II** will involve monitoring policy and programme implementation in publicly funded school settings in the selected primary and junior high schools.

**Instructions**

Obtain a copy of the monitoring and evaluation report conducted at least 12 months after implementation of the policy/programme. If more than two reports are available, conduct your assessment based on findings from the most recent report. Complete the overview of the monitoring data in section 1 and complete the details of the monitoring data in section 2.

In section 2, place a tick (✓) in the “yes” column if the monitoring data includes an assessment of the indicator, and note the findings in the last column. Place a tick (✓) in the “no” column if the monitoring data does not include an assessment of the indicator. Place a tick (✓) in the “I” column if the monitoring data does not include a complete or full assessment of the listed indicator.

1. **Overview of monitoring data currently available**

|  | | |
| --- | --- | --- |
| 1.1 Type of monitoring data | Self-report, e.g. reported by caterers, cooks, teachers [ ]  3^rd^ party review [ ] |  |
| 1.2 Sample description | Full sample [ ]  Subsample [ ] | Specify number and population characteristics |
| 1.3 Sanctions for non-compliance | Yes [ ] No [ ] | If yes, specify |
| 1.4 Assessment of frequency/proportion of sites that have implemented the policy/programme | Yes [ ] No [ ] | If yes, complete questions 2.1-2.4 |
| 1.4 Assessment of the nutritional quality or “healthfulness” of foods provided or sold relative to nutrition standards or guideline | Yes [ ] No [ ] | If yes, note down reference standard/guideline used here, and complete questions 2.5-2.7 |
| 1.4 Assessment of the nutritional quality or “healthfulness” of foods provided or sold **not** relative to nutrition standards or guideline | Yes [ ] No [ ] | If yes, detail here:  e.g. the assessment was conducted based on definitions of “healthy” and “unhealthy” developed by a research team; or the assessment reports on the most common foods provided or offered for sale. |

1. **Details of monitoring data currently available**

| **Indicator** | **Assessment Conducted?** | | | Write down findings from the report | |
| --- | --- | --- | --- | --- | --- |
|  | Y | N | I |  |  |
| 2.1 Does the report detail the number or proportion of schools **implementing** in the programme? |  |  |  | No. of schools surveyed: __________ No. of schools implementing: ___________  Percentage of schools Implementing: _____% | |
| 2.2 Does the report detail the number or proportion of students in schools **implementing** in the programme? |  |  |  | No. of schools surveyed: No. of students in implementing schools:  Percentage of students in implementing schools: | |
| 2.3 Does the report detail the number or proportion of schools **complying with** the programme? |  |  |  | No. of schools surveyed: No. of schools in compliance:  Percentage of schools in compliance: | |
| 2.4 Does the report detail the number or proportion of students in schools **complying with** the programme? |  |  |  | No. of schools surveyed: No. of students in complying schools:  Percentage of students in complying schools: | |
| 2.5 Does the report provide a quantitative assessment (relative to each food group-based standard/guideline)? |  |  |  | **% of foods meeting food group-based standards?** | e.g. Overall, 60% of **foods** adhered to the food-based guidelines (12 schools sampled) |
|  |  |  |  | **% of schools having or meeting food group-based standards?** | e.g. 80% of schools implementing the school food policy  89% of **schools** met the food-based standards for **snacks** (range of compliance varied between 32% and 100%) |
|  |  |  |  | **Foods or standards most or least compliant?** | e.g. Within the dairy category, yogurt parfaits were most compliant (72%); cheeses were least compliant (28%) |
| 2.6 Does the report provide a quantitative assessment (relative to each nutrient-based standard/guideline)? |  |  |  | **% of foods meeting nutrient-based standards?** | e.g. 30% of **foods** sold in primary schools met the standard for **sodium** |
|  |  |  |  | **% of schools meeting nutrient based standards?** | e.g. most primary **schools** (86%) met between 7 and 10 out of the **14** **nutrient and energy standards** |
|  |  |  |  | **Foods or standards most or least compliant?** | e.g. low sodium deli meats were the least (35%) compliant  e.g. low-fat dairy products were the most (87%) |
| 2.7 Does the report provide a qualitative assessment? |  |  |  | ***If yes, note down the findings from the qualitative assessment below;*** | |
| ***Findings from qualitative assessment*** | | | | | |
| e.g. canteen managers reported that the whole grain standards were the most difficult to meet, while low fat diary were the least difficult | | | | | |
| e.g. sodium standards were the most difficult to meet due to low availability of products meeting the standard | | | | | |
|  | | | | | |
|  | | | | | |
|  | | | | | |
|  | | | | | |
|  | | | | | |
|  | | | | | |
|  | | | | | |
|  | | | | | |
|  | | | | | |
|  | | | | | |
|  | | | | | |
|  | | | | | |

***Adapted from “Monitoring foods and beverages provided and sold in public sector settings” L’Abbe et. al., 2013***
