# Supplementary material for: Providing Measurement, Evaluation, Accountability, and Leadership Support (MEALS) for Non-communicable Diseases Prevention in Ghana: Project Implementation Protocol
Source: Front Nutr. 2021 Aug 18;8:644320. doi: 10.3389/fnut.2021.644320 (PMC8416277; doi:10.3389/fnut.2021.644320)
Supplement: Appendix 11 — Onsite observations and compliance assessment tool. [file Table_11.DOCX]

**NUTRITION STANDARDS/GUIDELINES AND NUTRITIONAL QUALITY OF PROVIDED AND SOLD FOODS**

**PROJECT TITLE: Measuring the Healthiness of Ghanaian Children's Food Environments to Prevent Obesity and Non-Communicable Diseases**

**PART 1 – ASSESSMENT OF NUTRITIONAL QUALITY OF PROVIDED FOODS**

**Desk Review - To be completed in office by trained personnel**

**Part 1A: Assessment of Nutritional Quality of Provided Foods Relative to Nutrition Standards or Guidelines Applied in School Food Programme/Policy**

**Instructions**

Obtain a copy of the food standards/guidelines applied to **provided foods** in the school food programme, if available and write these in Column 2. Obtain a 1-week school food menu, and check that the foods **provided** meet the standards/guidelines.

Place a tick (✓) in the “yes (Y)” column if the food served corresponds with the written standard. Place a tick (✓) in the “no (N)” column if the written standard is not met. Place a tick (✓) in the “not applicable (N/A)” column if the standard does not apply to the school.

1. **Food-based standards/guidelines for PROVIDED FOODS**

| **Food group** | **Standard guideline** | **Standard Met?** | | | **Proportion of Provided Foods complying with nutrition standards** |
| --- | --- | --- | --- | --- | --- |
|  |  | Y | N | N/A |  |
| **GRAIN (CEREAL) PRODUCTS**  Whole grain bread (seeded), whole brown bread, butter whole grain breakfast cereal (e.g. muesli cereals, Weetabix), maize (boiled, roasted), sorghum, local brown rice, white rice, pasta, millet, boiled corn meal, wheat porridge, sorghum porridge, millet porridge, banku, kenkey | Write here what the standard/guideline says about grain products e.g., any food **provided** in the school must be a grain product that contains 50% or more whole grains by weight or have whole grains as the first ingredient |  |  |  | e.g. 3 out of 5 menu meals met the nutrition standard for grain products |
| **MEAT AND ALTERNATIVES**  **Meat**  e.g. beef, goat, lamb, pork, bush meat, cat meat, wele (cow skin and cow feet)  **Poultry**  e.g. chicken, guinea fowl, turkey, duck.  **Fish**  e.g. tuna, tilapia, salmon, cassava fish, red fish, shellfish (Snail, clams (adodi), crab, oysters)  **Eggs, tofu, nuts and seeds and legumes/beans** | Write here what the standard/guideline says about meat and alternatives |  |  |  |  |
| **Milk and alternatives**  e.g. milk, plain yoghurt, flavoured yoghurt, cheese, waagashi cheese, burkina drink, sweetened condensed milk, powdered milk, evaporated milk, Soya milk, coconut milk, cream, sour cream, whipped cream | Write here what the standard/guideline says about milk and alternatives |  |  |  |  |
| **Fruits**  e.g. orange, tangerine, watermelon, mango, pawpaw, pineapple, banana, plum, peach, apricot, nectarine, flat peach, apple, pear, strawberries, cherries | Write here what the standard/guideline says about fruits e.g. each provided school meal should include one serving of fruit |  |  |  |  |
| **Vegetables**  e.g. green leaves, spinach, lettuce, cabbage, tomatoes, peppers, carrots, cucumber, eggplant, green beans, onions and garlic, mushrooms, | Write here what the standard/guideline says about vegetables e.g. each provided school meal should include one serving of vegetables |  |  |  |  |
| **Condiments and spices**  e.g. Ketch-up, mayonnaise, salad dressing, bouillon cubes; maggi, royco, onga | Write here what the standard/guideline says about condiments and spices e.g. ban on use of bouillon cubes in the preparation of school meals |  |  |  |  |
| **Foods and beverages high in calories, sugar, fat, or salt**  e.g. cakes, chocolates, candies, muffins, French fries, potato chips, alcohol, fruit flavoured drinks, soft drinks | Write here what the standard/guideline says about foods and beverages high in calories, sugar, fat, or salt  e.g. ban on provision of soft drinks or all foods in this category |  |  |  |  |

1. **Nutrient-based standards/guidelines for PROVIDED FOODS**

| **Nutrient** | **Standard guideline** | **Standard Met?** | | | **Proportion of food items/meals complying with nutrition standard** |
| --- | --- | --- | --- | --- | --- |
|  |  | Y | N | N/A |  |
| Energy | Write here what the standard/guideline says about Energy e.g., each provided school meal should provide no more than 700 kcal of energy; |  |  |  | e.g. 3 out of 7 menu meals met the energy requirement |
| Carbohydrate |  |  |  |  |  |
| Total fibre |  |  |  |  |  |
| Protein |  |  |  |  |  |
| Total fat |  |  |  |  |  |
| Saturated fat |  |  |  |  |  |
| Trans fats | Write here what the standard/guideline says about trans fats e.g., any food provided in the school should have 0 trans fat |  |  |  |  |
| Total sugars |  |  |  |  |  |
| Added sugars |  |  |  |  |  |
| Sodium |  |  |  |  |  |
| Potassium |  |  |  |  |  |
| Calcium |  |  |  |  |  |
| Phosphorus |  |  |  |  |  |
| Magnesium |  |  |  |  |  |
| Iron |  |  |  |  |  |
| Zinc |  |  |  |  |  |
| Thiamine (Vitamin B1) |  |  |  |  |  |
| Riboflavin (Vitamin B2) |  |  |  |  |  |
| Niacin (Vitamin B3) |  |  |  |  |  |
| Pantothenic acid (Vitamin B5) |  |  |  |  |  |
| Folate (Vitamin B9) |  |  |  |  |  |
| Vitamin C |  |  |  |  |  |
| **Caffeine** |  |  |  |  |  |
|  |  |  |  |  |  |
|  |  |  |  |  |  |

**Part 1B: Assessment of Nutritional Quality of Provided Foods Relative to Voluntary Nutrition Standards/Guidelines**

**Instructions**

Obtain a 1-week school food menu and list all provided foods in column 1.

Place a tick (✓) in the appropriate cell to indicate which of the categories of each of the three food classification systems (Core/Non-Core; NOVA Food Classification; DFC Food Classification) the **provided** food item belongs to.

|  | **Core/Non-Core** | | | **NOVA Classification** | | | | **DFC Food Classification** | | | | |
| --- | --- | --- | --- | --- | --- | --- | --- | --- | --- | --- | --- | --- |
| **Provided Food Item** | Core | Non-Core | Miscellaneous | Unprocessed or minimally processed foods | Processed culinary ingredients | Processed Foods | Ultra-processed food and drink products | Nutrient and energy density | | Food type | | |
|  |  |  |  |  |  |  |  | EDNP^[[1]](#footnote-1)^ | EDNR^[[2]](#footnote-2)^ | Fried Foods | Sweet foods | Sweetened beverages |
| **1.** |  |  |  |  |  |  |  |  |  |  |  |  |
| **2.** |  |  |  |  |  |  |  |  |  |  |  |  |
| **3.** |  |  |  |  |  |  |  |  |  |  |  |  |
| **4.** |  |  |  |  |  |  |  |  |  |  |  |  |
|  |  |  |  |  |  |  |  |  |  |  |  |  |
| **TOTAL:** |  |  |  |  |  |  |  |  |  |  |  |  |

**PART 2: ASSESSMENT OF NUTRITIONAL QUALITY OF SOLD FOODS**

**Desk Review - To be completed in office by trained personnel**

**Part 2A: Assessment of Nutritional Quality of Sold Foods Relative to Nutrition Standards or Guidelines Applied in School Food Programme/Policy**

**Instructions**

Obtain a copy of the food standards/guidelines applied to **sold foods** in the school food programme, if available and write these in Column 1. Obtain photographs of foods sold in each food outlet within the school canteen and check that the foods **sold** meet the standards/guidelines. Fill this tool separately for each food outlet where foods are sold to pupils.

Place a tick (✓) in the “yes (Y)” column if the food sold corresponds with the written standard. Place a tick (✓) in the “no (N)” column if the written standard is not met. Place a tick (✓) in the “not applicable (N/A)” column if the standard does not apply to the school.

1. **Food-based standards/guidelines for SOLD FOODS**

| **Food group** | **Standard guideline** | **Standard Met?** | | | **Proportion of SOLD FOODS complying with nutrition standards** |
| --- | --- | --- | --- | --- | --- |
|  |  | Y | N | N/A |  |
| **GRAIN (CEREAL) PRODUCTS**  Whole grain bread (seeded), whole brown bread, butter whole grain breakfast cereal (e.g. muesli cereals, Weetabix), maize (boiled, roasted), sorghum, local brown rice, white rice, pasta, millet, boiled corn meal, wheat porridge, sorghum porridge, millet porridge, banku, kenkey | Write here what the standard/guideline says about grain products e.g., any food **sold** within the school canteen, if a grain product must contain 50% or more whole grains by weight or have whole grains as the first ingredient |  |  |  |  |
| **MEAT AND ALTERNATIVES**  **Meat**  e.g. beef, goat, lamb, pork, bush meat, cat meat, wele (cow skin and cow feet)  **Poultry**  e.g. chicken, guinea fowl, turkey, duck.  **Fish**  e.g. tuna, tilapia, salmon, cassava fish, red fish, shellfish (Snail, clams (adodi), crab, oysters)  **Eggs, tofu, nuts and seeds and legumes/beans** |  |  |  |  |  |
| **Milk and alternatives**  e.g. milk, plain yoghurt, flavoured yoghurt, cheese, waagashi cheese, burkina drink, sweetened condensed milk, powdered milk, evaporated milk, Soya milk, coconut milk, cream, sour cream, whipped cream |  |  |  |  |  |
| **Fruits**  e.g. orange, tangerine, watermelon, mango, pawpaw, pineapple, banana, plum, peach, apricot, nectarine, flat peach, apple, pear, strawberries, cherries |  |  |  |  |  |
| **Vegetables**  e.g. green leaves, spinach, lettuce, cabbage, tomatoes, peppers, carrots, cucumber, eggplant, green beans, onions and garlic, mushrooms, |  |  |  |  |  |
| **Condiments and spices**  e.g. Ketch-up, mayonnaise, salad dressing, bouillon cubes; maggi, royco, onga |  |  |  |  |  |
| **Foods and beverages high in calories, sugar, fat, or salt**  e.g. cakes, chocolates, candies, muffins, French fries, potato chips, alcohol, fruit flavoured drinks, soft drinks | Write here what the standard/guideline says about foods and beverages high in calories, sugar, fat, or salt  e.g. ban on **sale** of soft drinks or all foods in this category |  |  |  |  |

1. **Nutrient-based standards/guidelines for SOLD FOODS**

| **Nutrient** | **Standard guideline** | **Standard Met?** | | | **Proportion of SOLD FOODS complying with nutrition standard** |
| --- | --- | --- | --- | --- | --- |
|  |  | Y | N | N/A |  |
| Energy | Write here what the standard/guideline says about energy e.g., each **sold** meal should provide no more than 700 kcal of energy; |  |  |  | e.g. 1 out of 2 sold meals met the energy requirement |
| Carbohydrate |  |  |  |  |  |
| Total fibre |  |  |  |  |  |
| Protein |  |  |  |  |  |
| Total fat |  |  |  |  |  |
| Saturated fat |  |  |  |  |  |
| Trans fats | Write here what the standard/guideline says about trans fats e.g., any food **sold** in the school should have 0 trans fat |  |  |  |  |
| Total sugars |  |  |  |  |  |
| Added sugars |  |  |  |  |  |
| Sodium |  |  |  |  |  |
| Potassium |  |  |  |  |  |
| Calcium |  |  |  |  |  |
| Phosphorus |  |  |  |  |  |
| Magnesium |  |  |  |  |  |
| Iron |  |  |  |  |  |
| Zinc |  |  |  |  |  |
| Thiamine (Vitamin B1) |  |  |  |  |  |
| Riboflavin (Vitamin B2) |  |  |  |  |  |
| Niacin (Vitamin B3) |  |  |  |  |  |
| Pantothenic acid (Vitamin B5) |  |  |  |  |  |
| Folate (Vitamin B9) |  |  |  |  |  |
| Vitamin C |  |  |  |  |  |
| **Caffeine** |  |  |  |  |  |
|  |  |  |  |  |  |
|  |  |  |  |  |  |

**Part 2B: Assessment of Nutritional Quality of Sold Foods Relative to Voluntary Nutrition Standards/Guidelines**

**Instructions**

Obtain a picture of the food items sold within each sold food outlet and list all sold foods in column 1.

Place a tick (✓) in the appropriate cell to indicate which of the categories of each of the three food classification systems (Core/Non-Core; NOVA Food Classification; DFC Food Classification) the **sold** food item belongs to.

|  | **Core/Non-Core** | | | **NOVA Classification** | | | | **DFC Food Classification** | | | | |
| --- | --- | --- | --- | --- | --- | --- | --- | --- | --- | --- | --- | --- |
| **Sold Food Item** | Core | Non-Core | Miscellaneous | Unprocessed or minimally processed foods | Processed culinary ingredients | Processed Foods | Ultra-processed food and drink products | Nutrient and energy density | | Food type | | |
|  |  |  |  |  |  |  |  | EDNP^[[3]](#footnote-3)^ | EDNR^[[4]](#footnote-4)^ | Fried Foods | Sweet foods | Sweetened beverages |
| **1.** |  |  |  |  |  |  |  |  |  |  |  |  |
| **2.** |  |  |  |  |  |  |  |  |  |  |  |  |
| **3.** |  |  |  |  |  |  |  |  |  |  |  |  |
| **4.** |  |  |  |  |  |  |  |  |  |  |  |  |
|  |  |  |  |  |  |  |  |  |  |  |  |  |
| **TOTAL:** |  |  |  |  |  |  |  |  |  |  |  |  |

1. ENDP: ***Energy Dense*** *(>225kcals/100g)* ***Nutrient Poor*** *(<10% for nutrient rich index score)*  [↑](#footnote-ref-1)
2. EDNR: ***Energy Dense*** *(>225 kcals/100g)* ***Nutrient Rich*** *(≥10% for nutrient rich index score)* [↑](#footnote-ref-2)
3. ENDP: ***Energy Dense*** *(>225kcals/100g)* ***Nutrient Poor*** *(<10% for nutrient rich index score)*  [↑](#footnote-ref-3)
4. EDNR: ***Energy Dense*** *(>225 kcals/100g)* ***Nutrient Rich*** *(≥10% for nutrient rich index score)* [↑](#footnote-ref-4)
