# Supplementary material for: Providing Measurement, Evaluation, Accountability, and Leadership Support (MEALS) for Non-communicable Diseases Prevention in Ghana: Project Implementation Protocol
Source: Front Nutr. 2021 Aug 18;8:644320. doi: 10.3389/fnut.2021.644320 (PMC8416277; doi:10.3389/fnut.2021.644320)
Supplement: Appendix 12 — Qualitative focus group discussion guide. [file Table_12.DOCX]

**Appendix 3XII - FGD Guide**

**PROJECT TITLE: Measuring the Healthiness of Ghanaian Children's Food Environments to Prevent Obesity and Non-Communicable Diseases**

**Date:**

**Time:**

**Location:**

**Moderator:**

**Note taker:**

**Participants’ background details here**

|  | First Name | Age (years) | Sex (M/F) | Grade: Eg classs 1, Form 1 | Name of school |
| --- | --- | --- | --- | --- | --- |
| 1 |  |  |  |  |  |
| 2 |  |  |  |  |  |
| 3 |  |  |  |  |  |
| 4 |  |  |  |  |  |
| 5 |  |  |  |  |  |
| 6 |  |  |  |  |  |
| 7 |  |  |  |  |  |
| 8 |  |  |  |  |  |
| 9 |  |  |  |  |  |
| 10 |  |  |  |  |  |

**Food Provision-related issues to discuss**

- Explore children’s understanding of **what a healthy diet is** (probe to receive elaborations on **their views on what a healthy diet is**. Is healthiness **based on food safety?** **Food hygiene?** **Issues of obesity?)**
- Explore **students’ understanding of factors that may contribute or cause malnutrition** (both undernutrition as well as obesity)
- Explore **frequent food choices (both provided and sold foods) and factors or reasons influencing food choices at school** (including food vendors, kiosks outside the school which they may access before, during and/or after school)
- Explore **children’s satisfaction with the provided and/or sold foods at school**
- Explore peer influence and interaction during lunchtime/snack time (**Does what children eat, prefer to eat influenced by what their peers eat?** If they say yes, probe to find out how? Also probe to record what measures they (children) put in place to ensure they get to eat those foods)
- Explore perceptions on time allocated to eating (e.g. How much is allocated to eating – Snack time, Lunch time. **Do you have enough time to finish eating your lunch?**)
- Explore water, sanitation and hygiene, food safety, and physical activity practices (**Ask children if they think their schools have these facilities and in good state or quality?)**

**Food Promotion-related issues to discuss**

- Assess children’s views on food adverts in general (**have they seen some –at home, at school—in their community? Which ones? what are they intended for?)**
- Assess children’s perceptions of any effect adverts might have on them and their peers (**Do children think such adverts may have an effect on them? Any effect; may probe to find if such effect will be their preferences, their choices, their intake)**
- Identify factors children find most and least appealing about food advertising (**Ask children to talk about aspects of food advertising that is most appealing to them. When this has been thoroughly discussed, engage children on aspects of food adverts that are least appealing to them**).
- Find out which groups children feel that food adverts are targeted at **(Let children discuss who they think food adverts are targeted at – adults, children, men, women, boys, girls, rich, poor etc)**
- Assess any food brands/companies they know (**Based on their previous exposure to food adverts, ask children to recall any food, food brand, or food company that they remember)**
- Explore **children’s views on marketing restrictions on certain products** (e.g. **should there be marketing restrictions on these --** trans fatty acids, non-sugar additives, sugar sweetened beverages**? If yes, if no, why not?** )
- Explore **children’s perceptions on Nutrition and Health Claims on food advertisement (do children believe in them? Or children have confidence in them? [the nutrition or health claims—moderator to give examples] if yes, why? If no, why not….?**
- Explore children’s perspectives on promoting healthy food (e.g. counter marketing; **should public health advocates, health experts or authorities [e.g. Ghana Health Service] deliberately market healthy foods to counter adverts of unhealthy foods?** ..if yes, why, if no why not)

Note to Moderator: Help every child participant to complete this table.

| **Child #1.**  **Name**  **Explore availability of the following categories of foods at home** | | | | |
| --- | --- | --- | --- | --- |
| **Available** | **Always** | **Most of the time** | **Sometimes** | **Not available at all** |
| Fruits |  |  |  |  |
| Dark green vegetables |  |  |  |  |
| Salty snacks |  |  |  |  |
| Fat-free low-fat milk |  |  |  |  |
| Soft drinks/Sugar sweetened beverages |  |  |  |  |

| **Child #2.**  **Name**  **Explore availability of the following categories of foods at home** | | | | |
| --- | --- | --- | --- | --- |
| **Available** | **Always** | **Most of the time** | **Sometimes** | **Not available at all** |
| Fruits |  |  |  |  |
| Dark green vegetables |  |  |  |  |
| Salty snacks |  |  |  |  |
| Fat-free low-fat milk |  |  |  |  |
| Soft drinks/Sugar sweetened beverages |  |  |  |  |

| **Child #3.**  **Name**  **Explore availability of the following categories of foods at home** | | | | |
| --- | --- | --- | --- | --- |
| **Available** | **Always** | **Most of the time** | **Sometimes** | **Not available at all** |
| Fruits |  |  |  |  |
| Dark green vegetables |  |  |  |  |
| Salty snacks |  |  |  |  |
| Fat-free low-fat milk |  |  |  |  |
| Soft drinks/Sugar sweetened beverages |  |  |  |  |

| **Child #4.**  **Name**  **Explore availability of the following categories of foods at home** | | | | |
| --- | --- | --- | --- | --- |
| **Available** | **Always** | **Most of the time** | **Sometimes** | **Not available at all** |
| Fruits |  |  |  |  |
| Dark green vegetables |  |  |  |  |
| Salty snacks |  |  |  |  |
| Fat-free low-fat milk |  |  |  |  |
| Soft drinks/Sugar sweetened beverages |  |  |  |  |

| **Child #5.**  **Name**  **Explore availability of the following categories of foods at home** | | | | |
| --- | --- | --- | --- | --- |
| **Available** | **Always** | **Most of the time** | **Sometimes** | **Not available at all** |
| Fruits |  |  |  |  |
| Dark green vegetables |  |  |  |  |
| Salty snacks |  |  |  |  |
| Fat-free low-fat milk |  |  |  |  |
| Soft drinks/Sugar sweetened beverages |  |  |  |  |

| **Child #6.**  **Name**  **Explore availability of the following categories of foods at home** | | | | |
| --- | --- | --- | --- | --- |
| **Available** | **Always** | **Most of the time** | **Sometimes** | **Not available at all** |
| Fruits |  |  |  |  |
| Dark green vegetables |  |  |  |  |
| Salty snacks |  |  |  |  |
| Fat-free low-fat milk |  |  |  |  |
| Soft drinks/Sugar sweetened beverages |  |  |  |  |

| **Child #7.**  **Name**  **Explore availability of the following categories of foods at home** | | | | |
| --- | --- | --- | --- | --- |
| **Available** | **Always** | **Most of the time** | **Sometimes** | **Not available at all** |
| Fruits |  |  |  |  |
| Dark green vegetables |  |  |  |  |
| Salty snacks |  |  |  |  |
| Fat-free low-fat milk |  |  |  |  |
| Soft drinks/Sugar sweetened beverages |  |  |  |  |

| **Child #8.**  **Name**  **Explore availability of the following categories of foods at home** | | | | |
| --- | --- | --- | --- | --- |
| **Available** | **Always** | **Most of the time** | **Sometimes** | **Not available at all** |
| Fruits |  |  |  |  |
| Dark green vegetables |  |  |  |  |
| Salty snacks |  |  |  |  |
| Fat-free low-fat milk |  |  |  |  |
| Soft drinks/Sugar sweetened beverages |  |  |  |  |

| **Child #9.**  **Name**  **Explore availability of the following categories of foods at home** | | | | |
| --- | --- | --- | --- | --- |
| **Available** | **Always** | **Most of the time** | **Sometimes** | **Not available at all** |
| Fruits |  |  |  |  |
| Dark green vegetables |  |  |  |  |
| Salty snacks |  |  |  |  |
| Fat-free low-fat milk |  |  |  |  |
| Soft drinks/Sugar sweetened beverages |  |  |  |  |

| **Child #10.**  **Name**  **Explore availability of the following categories of foods at home** | | | | |
| --- | --- | --- | --- | --- |
| **Available** | **Always** | **Most of the time** | **Sometimes** | **Not available at all** |
| Fruits |  |  |  |  |
| Dark green vegetables |  |  |  |  |
| Salty snacks |  |  |  |  |
| Fat-free low-fat milk |  |  |  |  |
| Soft drinks/Sugar sweetened beverages |  |  |  |  |
