# Supplementary material for: Providing Measurement, Evaluation, Accountability, and Leadership Support (MEALS) for Non-communicable Diseases Prevention in Ghana: Project Implementation Protocol
Source: Front Nutr. 2021 Aug 18;8:644320. doi: 10.3389/fnut.2021.644320 (PMC8416277; doi:10.3389/fnut.2021.644320)
Supplement: Appendix 13 — Qualitative KII guide for parents. [file Table_13.DOCX]

**Appendix 3XVI - Parent/Caregiver IDI Guide**

**PROJECT TITLE: Measuring the Healthiness of Ghanaian Children's Food Environments to Prevent Obesity and Non-Communicable Diseases**

**Date:**

**Time:**

**Location:**

**Interviewer:**

**Note taker:**

**Caregiver’s background details here**

| First Name | Area of residence | Relationship with child participant  e.g. Father  Mother  Other relative  House-help | Age of caregiver (years) | Sex of caregiver (M/F) | Name of Child FGD participant | Name of school the child participant goes to | Grade: of child participant  e.g.  Class 1, Form 1 |
| --- | --- | --- | --- | --- | --- | --- | --- |
|  |  |  |  |  |  |  |  |

**Food Provision-related Questions/issues to explore with parent**

- Explore parent/caregiver’s understanding of **what a healthy diet** is (probe to receive elaborations on their views on what a healthy diet is. Is healthiness based on food safety? Food hygiene? Issues of obesity/NCDs?)
- Explore parent’s understanding of **factors that may contribute or cause malnutrition** (both undernutrition as well as obesity)
- Explore parent’s **awareness of school nutrition policies/programmes**
- Explore parent’s **satisfaction with the provided and/or sold foods at school**

**Food Promotion-related Questions/issues to explore with parent**

- Explore parent’s **views on food adverts in general** (what are they intended for?)
- Assess **parent’s perceptions of any effect adverts might have on their child food literacy, food preferences, food choices** etc
- Find out **which groups parents feel that food adverts are targeted at** (children, the aged, celebrities, educated, illiterates, male, female etc)
- Explore their kn**owledge of advertising/marketing media, strategies or methods; techniques and their purpose** (e.g. marketing meda --- Marketing strategies/methods…….. etc)
- General recall of features of advertised product & brand recall (**Do parents recall adverts of food and non-alcoholic beverages?**. Let them give examples)
- Explore **parent’s views on marketing restrictions on certain products** (e.g. **should there be restrictgions on these? If yes, if no, why not?**  trans fatty acids, non-sugar additives, sugar sweetened beverages)
- Explore **parent’s perceptions on Nutrition and Health Claims on food advertisement (do parents believe or have confidence in them? [the claims] if yes, why? If no, why not….?**
- Explore parents perspectives on promoting healthy food (e.g. counter marketing; **should public health advocates, health experts or authorities [e.g. Ghana Health Service] deliberately market healthy foods to counter adverts of unhealthy foods?** ..if yes, why, if no why not)

**Home Food environment-related questions to explore with parent**

- Encouragement and modeling related to child eating [**do parents regularly or sometimes deliberately encourage their children to eat healthy foods, or deliberately purchase and consume such healthy foods – serving as a role-model for children**?
- Restrictive food practices **[do parents regularly or sometimes restrict what their children can buy, or eat? explore to find kinds of foods** e.g. “my children are not allowed to buy or consume sweets”**]**
- Pressure to eat [**do parents regularly or sometimes pressure their children to buy, or eat certain foods? Explore kinds of foods** e.g. “my children are not allowed to buy or consume sweets”**]**
- **Instrumental and emotional feeding** (food parenting practices in which food is used as a reward to control children’s behaviors (instrumental feeding) or emotions (emotional feeding).
- Do parents increase portion sizes of healthy foods [e.g. fruits and vegetables] or increase variety of foods, or decrease portion sizes of unhealthy foods….
- Do parents deliberately keep junk food out of home?
- Do parents provide reasoning for their food parenting actions? [food parenting practices that provide reasoning and education may be beneficial]
- Family rules related to child eating (**let each parent share examples if such rules exist at home)**
- Frequency of dinners out per week (**let each parent indicate how many times they have dinner out – per week)**
- Parent concern of healthy food costs **(let each parent indicate if they are concerned about cost of healthy foods)**

**Explore availability of the following categories of foods at home**

| **Available** | **Always** | **Most of the time** | **Sometimes** | **Not available at all** |
| --- | --- | --- | --- | --- |
| Fruits |  |  |  |  |
| Dark green vegetables |  |  |  |  |
| Salty snacks |  |  |  |  |
| Fat-free low-fat milk |  |  |  |  |
| Soft drinks/Sugar sweetened beverages |  |  |  |  |
