# Supplementary material for: Providing Measurement, Evaluation, Accountability, and Leadership Support (MEALS) for Non-communicable Diseases Prevention in Ghana: Project Implementation Protocol
Source: Front Nutr. 2021 Aug 18;8:644320. doi: 10.3389/fnut.2021.644320 (PMC8416277; doi:10.3389/fnut.2021.644320)
Supplement: Appendix 14 — CRM Key Informant tool. [file Table_14.DOCX]

**CRM Interview Guide**

**PROJECT TITLE: Measuring the Healthiness of Ghanaian Children's Food Environments to Prevent Obesity and Non-Communicable Diseases**

**Community Readiness Assessment Interview Guide**

**Demographics of respondent**

1. Gender:

□ Male □ Female

2. What is your work title? _______________________________________

3. What is your age range?

□ < = 18 yrs

□ 19-24 yrs

□ 25-34 yrs

□ 35-44 yrs

□ 45-54 yrs

□ 55-64 yrs

□ 65 yrs and above

4. Do you live in Greater Accra Region?

□ Yes □ No

If no: What area? ________________

5. How long have you lived in your area? ________________________

6. Do you work in Greater Accra Region?

□ Yes □ No

If no: What area? ________________

**Community Readiness Assessment Interview Questions**

In many questions in this survey I am going to ask you to respond to questions in relation to a scale. The scale will be from 1 to 10 and you will need to respond according to how much you agree with that particular question. For example, a question might ask: How much do you like oranges? You will need to respond on the scale from 1 to 10. If you very much like oranges, then you would respond “10”. But if you do not like oranges at all you would respond with a “1”. If you like it a little bit then you would respond with a “3” or if you somewhat like it then you would respond with “5” or “6” and if you like it you would respond “7” or “8”.

**For the following question, please answer keeping in mind your perspective of what community members believe and not what you personally believe.**

1. **Using a scale from 1-10, how much of a concern is *the issue of marketing and availability of unhealthy foods and drinks in and around child-setting institutions* in Greater Accra Region? (with 1 being “not at all” and 10 being “a very great concern”)**

1 🞎 2 🞎 3 🞎 4 🞎 5 🞎 6 🞎 7 🞎 8 🞎 9 🞎 10 🞎

Not at all Somewhat Very great

**Can you tell me why you think it’s at that level?***Interviewer: Please ensure that the respondent answers this question with regards to* ***community members*** *not themselves or what they think it should be.*

**COMMUNITY KNOWLEDGE OF EFFORTS**

I’m going to ask you about current community efforts to address marketing and availability of unhealthy foods and drinks in and around child-setting institutions in Greater Accra Region. By efforts, I mean any programmes, activities, or services in your community that address *marketing and availability of unhealthy foods and drinks in and around child-setting institutions.*

**2. Are there efforts in Greater Accra that address** **marketing and availability of unhealthy foods and drinks to children in and around child setting institutions?**

***If Yes, continue to question 3; if No or Don’t Know, skip to question 16***

**3. Can you briefly describe each of these?**

*Interviewer: Write down names of efforts so that you can refer to them in #4-5 below.*

4. How long have each of these efforts been going on? *Probe for each program/activity.*

5. Who do each of these efforts serve (e.g., a certain age group, ethnicity, etc.)?

**6. About how many community members are aware of each of the following aspects of the efforts - none, a few, some, many, or most? Can you explain why you say this?**

- **Have heard of efforts?
  □ None □ a few □ some □ many □ most** **□ don’t know □**
- **Can name efforts?**
  **□ None □ a few □ some □ many □ most □ don’t know □**
- **Know the purpose of the efforts?**
  **□ None □ a few □ some □ many □ most □ don’t know □**
- **Know who the efforts are for?**
  **□ None □ a few □ some □ many □ most □ don’t know □**
- **Know how the efforts work (e.g. activities or how they’re implemented)?**
  **□ None □ a few □ some □ many □ most □ don’t know □**
- **Know the effectiveness of the efforts?**

**□ None □ a few □ some □ many □ most □ don’t know □**

**7. Thinking back to your answers, why do you think members of your community have this amount of knowledge?**

**8. Are there misconceptions or incorrect information among community members about the current efforts?** *If yes:* What are these?

9. How do community members learn about the current efforts?

10. Do community members view current efforts as successful?

*Probe:* What do community members like about these programs? What don’t they like?

11. What are the obstacles to individuals participating in these efforts?

12. What are the strengths of these efforts?

13. What are the weaknesses of these efforts?

14. Are the evaluation results being used to make changes in efforts or to start new ones?
15. What planning for additional efforts to address marketing and availability of unhealthy foods and drinks in and around child-setting institutions is going on in *Greater Accra Region*?

*Only ask #16 if the respondent answered “No” to #2 or was unsure.*

**16. Is anyone in** *Greater Accra Region* **trying to get something started to address marketing and availability of unhealthy foods and drinks to children? Can you tell me about that?**

***LEADERSHIP***

I’m going to ask you how the leadership in *Greater Accra Region* perceives marketing and availability of unhealthy foods and drinks in and around child-setting institutions. By leadership, we are referring to those who could affect the outcome of this issue and those who have influence in the community and/or who lead the community in helping it achieve its goals.

**17. Using a scale from 1-10, how much of a concern is** marketing and availability of unhealthy foods and drinks in and around child-setting institutions **to the leadership of** *Greater Accra***, with 1 being “not a concern at all” and 10 being “a very great concern”?
 1 🞎 2 🞎 3 🞎 4 🞎 5 🞎 6 🞎 7 🞎 8 🞎 9 🞎 10 🞎**

**Not at all Somewhat Very much**

**Can you tell me why you say it’s a _____?**

**17a. How much of a priority is addressing the** marketing and availability of unhealthy foods and drinks in and around child-setting institutions **to leadership?**

**1 🞎 2 🞎 3 🞎 4 🞎 5 🞎 6 🞎 7 🞎 8 🞎 9 🞎 10 🞎**

**Not at all Somewhat Very much**

**Can you explain why you say this?**

**18. I’m going to read a list of ways that leadership might show its support or lack of support for efforts to address** marketing and availability of unhealthy foods and drinks in and around child-setting institutions

**Can you please tell me whether none, a few, some, many or most leaders would or do show support in this way? Also, feel free to explain your responses as we move through the list.**

**How many leaders…**

- **At least passively support efforts without necessarily being active in that support?**

**□ None □ a few □ some** **□ many □ most □**

- **Participate in developing, improving or implementing efforts, for example by being a member of a group that is working toward these efforts?
  □ None □ a few □ some □ many □ most □**
- **Support allocating resources to fund community efforts?
  □ None □ a few □ some □ many □ most □**
- **Play a key role as a leader or driving force in planning, developing or implementing efforts? *(prompt: How do they do that?)*
  □ None □ a few □ some □ many □ most □**
- **Play a key role in ensuring the sustainability of community efforts, for example by allocating long-term funding?
  □ None □ a few □ some □ many □ most □**

**19. Does the leadership support expanded efforts in the community to address** *marketing and availability of unhealthy foods and drinks in and around child-setting institutions***?**

*If* ***yes****:* **How do they show this support? For example, by passively supporting, by being involved in developing the efforts, or by being a driving force or key player in achieving these expanded efforts?**

20. Who are leaders that are supportive of addressing this issue in your community?

21. Are there leaders who might oppose addressing *marketing and availability of unhealthy foods and drinks in and around child-setting institutions*? How do they show their opposition?

***COMMUNITY CLIMATE***

For the following questions, again please answer keeping in mind your perspective of what community members believe and not what you personally believe.

**22. How much of a priority is addressing this issue** (*marketing and availability of unhealthy foods and drinks in and around child-setting institutions*) **to community members?**

1 🞎 2 🞎 3 🞎 4 🞎 5 🞎 6 🞎 7 🞎 8 🞎 9 🞎 10 🞎

Not at all Somewhat Very much

**Can you explain your answer?**

**23. I’m going to read a list of ways that community members might show their support or their lack of support for community efforts to address** *marketing and availability of unhealthy foods and drinks in and around child-setting institutions***.**

**Can you please tell me whether none, a few, some, many or most community members would or do show their support in this way? Also, feel free to explain your responses as we move through the list.**

**How many community members…**

 **At least passively support community efforts without being active in that support?**

**□ None □ a few □ some □ many □ most**

 **Participate in developing, improving or implementing efforts, for example by attending group meetings that are working toward these efforts?**

**□ None □ a few □ some □ many □ most**

 **Play a key role as a leader or driving force in planning, developing or implementing efforts? (prompt: How do they do that?)**

**□ None □ a few □ some □ many □ most**

 **Are willing to pay more (for example, in taxes) to help fund community efforts?**

**□ None □ a few □ some □ many □ most**

**24. About how many community members would support expanding efforts in the community to address** (marketing and availability of unhealthy foods and drinks in and around child-setting institutions)**? Would you say none, a few, some, many or most?**

*If* ***more than none:* How might they show this support? For example, by passively supporting or by being actively involved in developing the efforts?**

25. Are there community members who oppose or might oppose addressing *marketing and availability of unhealthy foods and drinks in and around child-setting institutions*? How do or will they show their opposition?

26. Are there ever any circumstances in which members of *Greater Accra Region* might think that this issue should be tolerated? Please explain.

27. Describe (*Greater Accra Region*).

***KNOWLEDGE ABOUT THE ISSUE***

28. On a scale of 1 to 10 where a 1 is no knowledge and a 10 is detailed knowledge, how much do community members know about (*issue*)?

1 🞎 2 🞎 3 🞎 4 🞎 5 🞎 6 🞎 7 🞎 8 🞎 9 🞎 10 🞎

None Somewhat A lot

Why do you say it’s a ____?

**29. Would you say that community members know nothing, a little, some or a lot about each of the following as they pertain to** *marketing and availability of unhealthy foods and drinks in and around child-setting institutions*? (*After each item, have them answer.)*

**29a. How much would you say that community members know about *marketing and availability of unhealthy foods and drinks in and around child-setting institutions*, in general (prompt as needed with “nothing, a little, some or a lot”).**

**□ Nothing □ a little □ some □ a lot □**

**29b. How much would you say that community members know about the causes of *marketing and availability of unhealthy foods and drinks in and around child-setting institutions*?**

**□ Nothing □ a little □ some □ a lot □**

**29c. How much would you say that community members know about the consequences of *marketing and availability of unhealthy foods and drinks in and around child-setting institutions*?**

**□ Nothing □ a little □ some □ a lot □**

**29d.** **How much would you say that community members know about whether** ***marketing and availability of unhealthy foods and drinks in and around child-setting institutions* occurs locally in your community?**

**□ Nothing □ a little □ some □ a lot □**

**29e. How much would you say that community members know about what can be done to prevent *marketing and availability of unhealthy foods and drinks in and around child-setting institutions***

**□ Nothing □ a little □ some □ a lot □**

**29f.** **How much would you say that community members know about the effects of** ***marketing and availability of unhealthy foods and drinks in and around child-setting institutions* on family and friends?**

**□ Nothing □ a little □ some □ a lot □**

**30. What are the misconceptions among community members about *marketing and availability of unhealthy foods and drinks in and around child-setting institutions*
 e.g. why it occurs, how much it occurs locally, or what the consequences are?**

31. What type of information is available in (*community)* about *marketing and availability of unhealthy foods and drinks in and around child-setting institutions*

(e.g. newspaper articles, brochures, posters)?

*If they list information, ask:* Do community members access and/or use this information?

***RESOURCES FOR EFFORTS*** *(time, money, people, space, etc.)*

*If there are efforts to address the issue locally, begin with question 33. If there are no efforts, go to question 33.*

**32. How are current efforts funded? Is this funding likely to continue into the future?**

**33. I’m now going to read you a list of resources that could be used to address** *(marketing and availability of unhealthy foods and drinks in and around child-setting institutions)* **in your community.**

**For each of these, please indicate whether there is none, a little, some or a lot of that resource available in your community that could be used to address** (*marketing and availability of unhealthy foods and drinks in and around child-setting institutions*)**?**

 **Volunteers?**

**□ None □ a little □ some □ a lot** **□ don’t know □**

 **Financial donations from organizations and/or businesses?**

**□ None □ a little □ some □ a lot □ don’t know □**

 **Government funding?**

**□ None □ a little □ some □ a lot □ don’t know □**

 **Experts?**

**□ None □ a little □ some □ a lot □ don’t know □**

 **Space?**

**□ None □ a little □ some □ a lot □ don’t know □**

**34. Would community members and leadership support using these resources to address** *marketing and availability of unhealthy foods and drinks in and around child-setting institutions***? Please explain.**

**35. On a scale of 1 to 5, where 1 is no effort and 5 is a great effort, how much effort are community members and/or leadership putting into doing each of the following things to increase the resources going toward addressing** *marketing and availability of unhealthy foods and drinks in and around child-setting institutions* **in your community?**

 **Seeking volunteers for current or future efforts to address** *marketing and availability of unhealthy foods and drinks in and around child-setting institutions* **in the community.**

 **Soliciting donations from businesses or other organizations to fund current or expanded community efforts.**

 **Writing grant proposals to obtain funding to address** *marketing and availability of unhealthy foods and drinks in and around child-setting institutions* **in the community.**

 **Training community members to become experts.**

 **Recruiting experts to the community.**

**36. Are you aware of any proposals or action plans that have been submitted for funding to address** *marketing and availability of unhealthy foods and drinks in and around child-setting institutions* **in** *Greater Accra Region***?**

***If Yes:* Please explain.**
