# Supplementary material for: Providing Measurement, Evaluation, Accountability, and Leadership Support (MEALS) for Non-communicable Diseases Prevention in Ghana: Project Implementation Protocol
Source: Front Nutr. 2021 Aug 18;8:644320. doi: 10.3389/fnut.2021.644320 (PMC8416277; doi:10.3389/fnut.2021.644320)
Supplement: Appendix 15 — Net-Mapping tool. [file Table_15.DOCX]

Net-Map Interview Guide

Meals4NCDs-Food Promotion

## **Key question: Who influences Food promotion and its influence on addressing Non-Communicable disease control among adolescents in Ghana?**

## Step 1 - Actor generation:

**Who is involved in policy and programs related to promoting and communicating messages on food and diets to children (9-15 years) in Ghana?**

We will start by listing all the organizations involved in policy and programs related to promoting messages on food and diets and its linkages to NCDs to children (9-15 years) in Ghana. (Prompt according to the different categories below.)

- Government………………………………………..………………**Green**
- Donor……………………………………………………….………...**Yellow**
- INGO/NGO/Civil Society……………………………….……… **Pink**
- UN…………………………………………………………………..… **Blue**
- Private sector……………………………………………………..**Orange**
- Research/Academia…………………………………………….Brown
- Media………………………………………………………………….Deep blue

*****Observer takes a photo of the poster paper***

## Step 2: Drawing links

**For each of the following links, who gives/has_________to/over who in the network?**

1. **Formal command**
2. **Funding or technical assistance**
3. **Advocacy**
4. **Dissemination of evidence-based information**

Draw the links one at a time, completing all of one type of link before moving on to the next. There may be overlap. Important to show the direction of the link.

1. Formal command: draw a link if an actor has formal oversight over the work or actions of another actor
2. Funding or technical assistance: draw a link if an actor provides funds or in-kind support (such as technical support or embedded advisors) to another actor, specifically related to food promotion
3. Advocacy or evidence: draw a link if an actor advocates for or champions issues related to food promotion. Eg calls upon decision makers give priority to food promotion
4. Dissemination of evidence-based information: draw a link if an actor is actively involved in circulating or distributing information to other actors.

*****Observer takes a photo of the poster paper***

Step 3: Opinion leaders:

**Are there any individuals that you would describe as opinion leaders of policy, programming and opinion related to diets and food promotion?**

Now focus on eliciting names of actors that are considered opinion leaders on the issue. They may be individuals embedded within some of the institutions already on the map, or they may be individuals (independent, or event retired). Add them to the map with a new color sticky note.

*****Observer takes a photo of the poster paper***

## Step 4: Attribute influence

**How influential is each actor for promoting food and diets to address NCDs in Ghana? Rate each actor’s influence on a scale of zero to five (0=not influential at all; 5=highest level of influence**

1. Define influence in this context:
   1. To what extent can the actors on the map influence or determine policies and programs to promote diets and foods linked with NCDs? Influence may be due to: control over funding flows, responsibility for implementation of key programs, being highly respected, having authority over policies or relevant decisions, etc.
2. Attributing influence: Starting with the actor with the highest influence, place between 0-5 checker pieces on each actor. Probe “why” they are influential or not for each one.
3. Review each score; compare and contrast influence levels across actors, probing further about why they are influential.

Meals4NCDs-Food Provisioning to children (9-15 years)

## **Key question: Who influences Food provisioning and its influence on addressing Non-Communicable disease control among children (9-15 years) in Ghana?**

## Step 1 - Actor generation:

**Who is involved in policy and programs related to food provisioning to children (9-15 years) in Ghana?**

We will start by listing all the organizations involved in food provisioning and its linkages to NCDs to children (9-15 years) in Ghana. (Prompt according to the different categories below.)

- Government………………………………………..………………**Green**
- Donor……………………………………………………….………...**Yellow**
- INGO/NGO/Civil Society……………………………….……… **Pink**
- UN…………………………………………………………………..… **Blue**
- Private sector……………………………………………………..**Orange**
- Research/Academia…………………………………………….Brown
- Media………………………………………………………………….Deep blue

*****Observer takes a photo of the poster paper***

## Step 2: Drawing links

**For each of the following links, who gives/has_________to/over who in the network?**

1. **Formal command**
2. **Funding or technical assistance**
3. **Advocacy**
4. **Dissemination of evidence-based information**

Draw the links one at a time, completing all of one type of link before moving on to the next. There may be overlap. Important to show the direction of the link.

1. Formal command: draw a link if an actor has formal oversight over the work or actions of another actor
2. Funding or technical assistance: draw a link if an actor provides funds or in-kind support (such as technical support or embedded advisors) to another actor, specifically related to food provisioning to children (9-15 years)
3. Advocacy or evidence: draw a link if an actor advocates for or champions issues related to food provisioning to children (9-15 years). Eg calls upon decision makers give priority to food promotion
4. Dissemination of evidence-based information: draw a link if an actor is actively involved in circulating or distributing information to other actors.

*****Observer takes a photo of the poster paper***

Step 3: Opinion leaders:

**Are there any individuals that you would describe as opinion leaders of policy, programming and opinion related to food provisioning to children (9-15 years)?**

Now focus on eliciting names of actors that are considered opinion leaders on the issue. They may be individuals embedded within some of the institutions already on the map, or they may be individuals (independent, or event retired). Add them to the map with a new color sticky note.

*****Observer takes a photo of the poster paper***

## Step 4: Attribute influence

**How influential is each actor for food provisioning to children (9-15 years) in Ghana? Rate each actor’s influence on a scale of zero to five (0=not influential at all; 5=highest level of influence**

1. Define influence in this context:
   1. To what extent can the actors on the map influence or determine policies and programs on food provisioning to children (9-15 years) in GHana? Influence may be due to: control over funding flows, responsibility for implementation of key programs, being highly respected, having authority over policies or relevant decisions, etc.
2. Attributing influence: Starting with the actor with the highest influence, place between 0-5 checker pieces on each actor. Probe “why” they are influential or not for each one.
3. Review each score; compare and contrast influence levels across actors, probing further about why they are influential.
